# Magnetic medical microrobots with memory-capable genetic circuits

**DOI:** 10.1126/sciadv.aeb2528

**Published:** 2026-05-13

**Authors:** Haotian Chen, Yujun Chen, Yingying Wang, Peng Ning, Yingze Li, Yuantai Sun, Xueyan Wei, Rui Gao, Xing Fan, Feng Tao, Zihan Guo, Weicheng Gu, Zhenguang Li, Xinjian Fan, Zhen Yin, Yu Cheng

**Affiliations:** ^1^ State Key Laboratory of Autonomous Intelligent Unmanned Systems, Shanghai Key Laboratory of Anesthesiology and Brain Functional Modulation, Clinical Research Center for Anesthesiology and Perioperative Medicine, Translational Research Institute of Brain and Brain-Like Intelligence, Collaborative Innovation Center for Brain Science, Shanghai Fourth People’s Hospital, School of Medicine, Tongji University, Shanghai 200434 China.; ^2^ Shanghai Innovation Institute, Shanghai 200030 China.; ^3^ School of Mechanical and Electric Engineering, Soochow University, Suzhou 215123 China.; ^4^ Department of Control Science and Engineering, Tongji University, Shanghai 201804 China.

## Abstract

Autonomous microrobots can reach hard-to-access regions in the human body for minimally invasive therapy. However, their microscale size limits the integration of on-board memory, making their operation dependent on external controls. Here, we develop a magnetic probiotic microrobot integrated with memory-capable genetic circuit to execute autonomous antitumor treatment. Through a one-time magnetic hyperthermia trigger, the biological thermal sensor in the microrobot perceives temperature change and activates the memory module Bxb1-ssrA-*attB*-P7-*attP*, transferring the microrobots into a therapeutic state to continuously degrade fibrin and soften the tumor microenvironment. The genetic memory remains active for at least 12 days. A synergy toward deep tumor penetration is subsequently established between the memory-encoded softening and the physical penetration through magnetically controlled wave-like locomotion of microrobots. Compared with memory-absent microrobots, the proposed microrobots achieve a 6.70-fold tumor matrix stiffness reduction and boost in vivo anticancer efficacy from 21.86 to 87.52%. Beyond oncology, the proposed system establishes a generalizable framework of memory-encoded medical microrobots.

## Teaser

Memory-encoded magnetic microrobots soften tumors and penetrate deeply for autonomous cancer therapy.

## INTRODUCTION

Medical microrobots can traverse complex and inaccessible areas within the human body to perform targeted drug delivery and complete minimally invasive procedures, positioning them at the forefront of medical research and development (*1*–*8*). Although they can be wirelessly controlled via magnetic fields, light, ultrasound, chemical gradients, and bioenergy (*9*–*18*), current microrobots lack on-board memory, leaving them unable to execute tasks once external stimuli are withdrawn. Integrating on-board memory modules is essential for medical microrobots to perform autonomous medical operations without needing consistent external inputs, but this remains as a bottleneck at the micrometer scale.

Extensive research efforts have been devoted to the development of small-scale robotic systems with memory capabilities. A typical approach is to use shape-memory materials and microelectronic components to achieve physical memory (*19*–*24*). Due to the challenges in high-throughput fabrication and microsystem integration (table S1), these memory-capable devices typically have a featured size of at least 10^0^ mm or larger, which are more than two orders higher than microrobots. On top of that, synthetic materials used in physical memory components can raise biocompatibility concerns when used inside the human body. Therefore, the development of biocompatible and economical memory-capable microrobots eventually determines the future of medical microrobots for real-world clinical applications.

Microorganisms are sophisticated machines composed of functional molecular components that have the capabilities to sense, memorize, communicate, actuate, and synthesize (*25*–*28*). Among these, probiotics have evolved to maintain a symbiosis with human beings, offering themselves as a highly biocompatible microrobotic platform (*29*, *30*). However, natural probiotics are not purposefully designed to accommodate medical tasks and are limited in controllability. By using microorganisms as templates, gene editing in synthetic biology provides an effective approach to manipulate and reconfigure biomolecular machine components (*31*–*36*), enabling the development of controllable microrobots. For instance, biosensors based on gene-edited probiotics have been developed to diagnose symptoms such as inflammation and infection (*37*, *38*). Memory-capable modules based on the recombinase Bxb1 have also been identified and could potentially be integrated into microrobotic systems (*39*–*41*). Therefore, the biosynthetic approach based on gene editing techniques offers a promising system to design and produce highly integrated memory-capable microrobots.

**Fig. 1.**
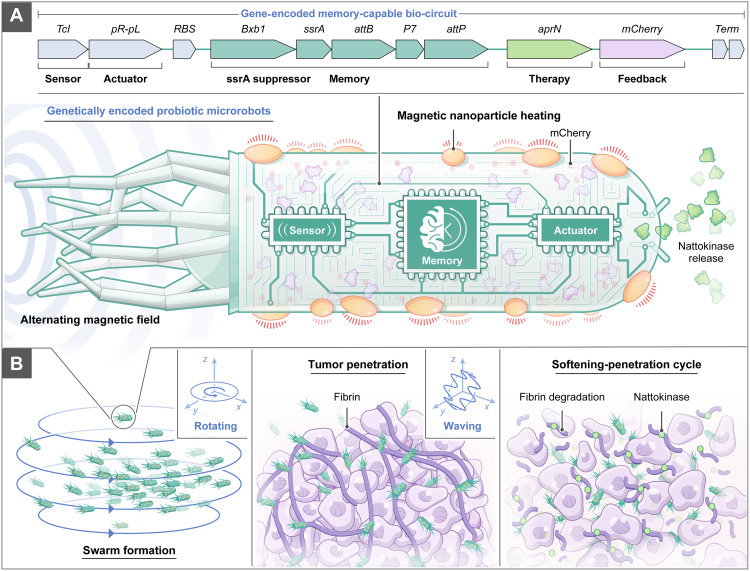
Schematic illustration of the probiotic microrobot swarm with genetic memory for amplifying antitumor efficacy. (**A**) Engineered probiotic EcN serves as the biological robotic platform, incorporating a thermal sensor, an actuator, a memory module, a memory suppressor, a biosynthetic module for NK release, and an mCherry fluorescence reporter for visual feedback. Upon sensing temperature increase by magnetic hyperthermia, the microrobots memorize the stimulus and autonomously release NK. (**B**) The effect of antitumor mechanoregulation is further boosted by the positive feedback loop established by the synergy between NK-induced tumor softening and magnetically enhanced tumor penetration.

Here, we designed a magnetic probiotic microrobot engineered with memory-capable biocircuit and magnetic controllability to achieve a synergy of tumor softening and physical penetration for autonomous antitumor treatment (Fig. 1). A genetic circuit containing five functional modules, including the thermal sensing, actuating, memory, therapy, and imaging feedback units, was encoded into probiotic *Escherichia coli* Nissle 1917 (EcN), followed by surface modification with magnetic nanoparticles (MNPs). The microrobots converted an exerted magnetic signal into heat via MNPs, activating genetically encoded thermal sensors that could detect and remember the thermal stimulation. This enabled the continuous synthesis of the fibrin degradation agent nattokinase (NK) and fluorescent mCherry for imaging feedback. In a mouse cancer model, the microrobot swarm with genetically encoded memory maintained long-term tumor softening capacity after thermal activation, without requiring continuous external magnetic input. Synergistically, the three-dimensional (3D) waving motion of the probiotic microrobot swarm enhanced tumor penetration by creating a softening-penetration positive feedback loop to tune tumor matrix stiffness and elevate antitumor efficacy in vivo. The demonstrated system platform establishes a translational framework for designing medical microrobots to autonomously treat diseases such as cancer.

## RESULTS

### Design and fabrication of gene-encoded magnetic medical microrobots

**Fig. 2.**
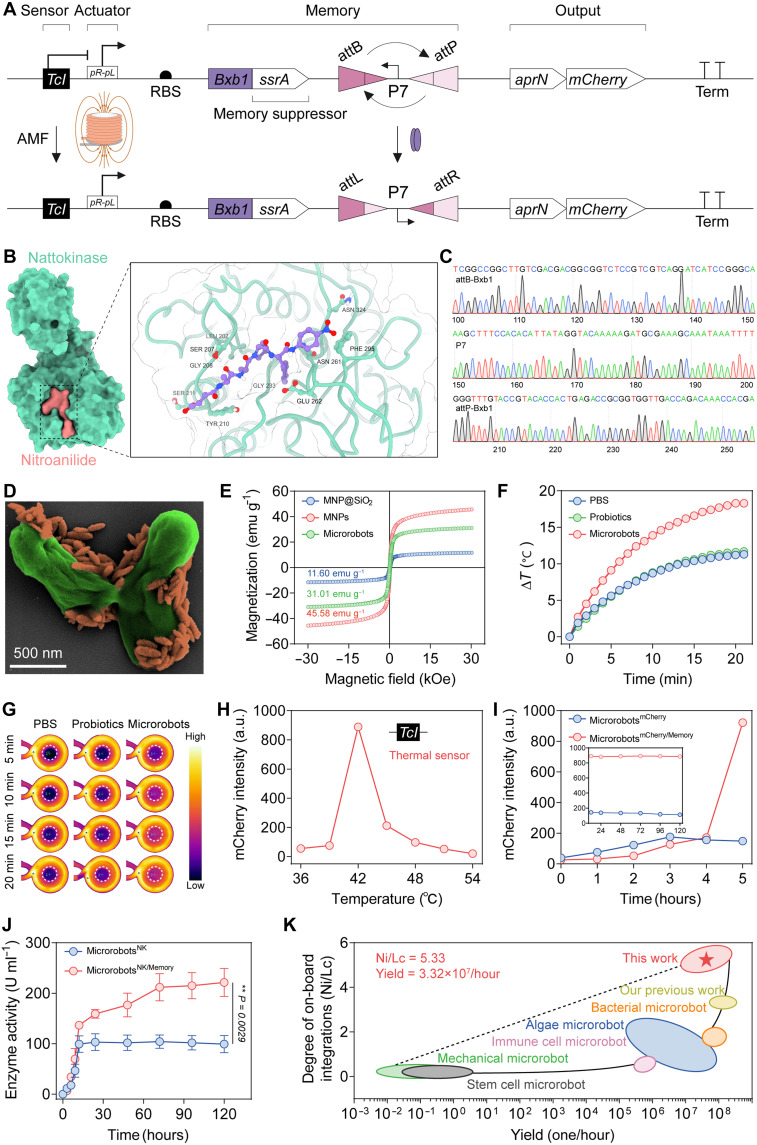
Design and characterization of gene-encoded magnetic medical microrobots with memory-capable integrated biocircuit. (**A**) Schematic illustration of the genetic circuit in probiotic microrobots. (**B**) Molecular docking analysis of modeled NK with a modeled tetrapeptide substrate (*N*-succinyl-Ala-Ala-Pro-Phe-*p*-nitroanilide) was conducted to verify the rationality of the NK gene sequence design. (**C**) The representative sequencing results of memory module gene sequences in genetic circuit, including *attB*-Bxb1, *attP*-Bxb1, and P7. (**D**) Pseudocolored SEM image of two probiotic microrobots. Green pseudocolor indicates probiotics, and brown pseudocolor indicates MNPs. (**E**) Magnetic hysteresis curves of MNP@SiO_2_, MNPs, and probiotic microrobots at ±30 kOe. (**F** and **G**) Temperature elevation and real-time thermal images of the microrobots (375 kHz, 330 Oe, 20 min). (**H**) Response of thermal sensors at different temperatures, with activation occurring at around 42°C (*n* = 3; means ± SD). (**I**) The mCherry fluorescence intensity of probiotic microrobots with or without memory devices was detected after a one-time magnetic hyperthermia intervention (*n* = 3; means ± SD). (**J**) The NK release comparison of microrobots with and without memory, showing the autonomous effect by the memory module (*n* = 3; means ± SD). (**K**) A comparative analysis of the degree of on-board integration (Ni/Lc) and yield of microrobots reported in the literature (table S3). Ni refers to the number of integrated on-board functions. Lc refers to the characteristic length of the microrobots. Statistical analysis was performed using unpaired *t* test for (J). a.u., arbitrary unit; RBS, ribosome binding sites.

The fabrication of gene-encoded magnetic medical microrobots with memory-capable integrated biocircuit mainly consisted of two steps: engineering genetically encoded memory biocircuit and coupling iron oxide nanoparticles onto the microrobots. In the first step, the biocircuit consisted of TcI (temperature-sensitive bacteriophage λ repressor cI857) as the thermal sensor, pR-pL (phage lambda thermally inducible promoters) as the actuator, Bxb1-ssrA-*attB*-P7-*attP* as the memory module, a biosynthetic module to release tumor softening agent (NK), and fluorescent mCherry for visual feedback (Fig. 2A). At body temperature, the TcI sensor suppressed the pR-pL actuator. When the environmental temperature exceeded 42°C, this suppression was lifted, enabling the pR-pL actuator to activate Bxb1 expression for *attB* (attachment site bacteria)/*attP* (attachment site phage) recombination. This recombination subsequently triggered the permanent inversion of P7, allowing the biocircuit to memorize the triggering state and maintain a self-sustained biosynthesis process. To prevent unregulated Bxb1 synthesis from imposing excessive stress to the probiotics, we fused the ssrA (small stable RNA molecule) degradation tag to Bxb1 to ensure its degradation by endogenous probiotic proteases (*42*, *43*). The full-length NK gene, known as “*aprN*,” consisting of a signal peptide (*pre*), pro-peptide (*pro*), and mature peptide (*nk*), was integrated into genetic circuit to ensure secretory output (table S2). Subsequently, molecular dynamics simulations were conducted to assess the rationality of the designed NK genetic code (Fig. 2B and fig. S1, A to D). Docking and molecular dynamics simulations revealed that the U-shaped structure of NK fit snugly within the tetrapeptide, forming a stable dimer structure (Fig. 2B). The root mean square fluctuation analysis of the tetrapeptide substrate showed that most atoms remained stable, indicating a firm binding to NK’s active sites to ensure high structural stability (fig. S1E). The calculated binding free energy between NK and the tetrapeptide substrate was −68.38 kcal mol^−1^, confirming a strong protein-ligand interaction (fig. S1F). Sanger sequencing further verified the assembly and encoding of the genetic circuits, with gene sequences matching the designed constructs (Fig. 2C and table S2). The memory circuit maintains high genetic and functional fidelity over extended propagation (120 consecutive generations) (fig. S2) (*44*).

To achieve magnetothermal conversion and controllable locomotion, we conjugated the rod-shaped magnetic Fe_3_O_4_ nanoparticles (MNPs: 230.31 ± 29.71 nm, with an aspect ratio of 3.15 ± 0.18) on the genetically engineered probiotics as the external magnetic sensor (fig. S3, A to E). The magnetization hysteresis curve showed that the synthesized MNPs exhibited ferrimagnetic properties with a 45.58 electromagnetic unit (emu) g^−1^ saturation magnetization (Fig. 2E). The temperature of MNPs steadily increased by 21.6°C under an alternating magnetic field (AMF) stimulation (375 kHz, 330 Oe, 20 min), exhibiting effective magnetothermal effect and stability (figs. S3, F to H, and S4). Subsequently, the MNPs were functionalized with amino groups (─NH_2_) and linked to the carboxyl group of *N*-acetylmuramic acid (─COOH) on the surface of probiotics via amide condensation (fig. S5, A and B). The optimal magnetic properties of microrobots were 31.01 emu g^−1^ by adjusting the concentration of modified MNPs (Fig. 2E and figs. S5C and S6). Scanning electron microscopy (SEM), bio–transmission electron microscopy, and laser scanning confocal microscopy (LSCM) imaging all confirmed that the MNPs were coupled on the microrobots (Fig. 2D and fig. S5, D and E) with 98.93 ± 0.68% conjugation efficiency (fig. S5, F and G).

Subsequently, we verified that the MNP-coated microrobots could convert external magnetic signals into heat. When exposed to an external AMF (375 kHz, 330 Oe), the temperature of the microrobots increased by 18.3°C within 20 min (Fig. 2, F and G), demonstrating effective conversion of magnetic signals into thermal signals. We then evaluated whether the thermal signals could be detected by the internal genetically encoded thermal sensor TcI, thereby triggering subsequent memory and biosynthesis. By adjusting the AMF duration, it was found that the fluorescence intensity of mCherry peaked at 42°C, confirming that the thermal sensing and memory-capable circuit was functional and precisely regulated (Fig. 2H and fig. S7). With the thermal sensing and memory capable biocircuit function validated, the performance of memory-absent and memory-equipped microrobots was further compared following a one-time magnetic hyperthermia intervention (fig. S8). Memory-equipped microrobots exhibited mCherry fluorescence nearly 7.8-fold higher than memory-absent counterparts after 120 hours (Fig. 2I). Correspondingly, the integration of memory module further improved the duration and dosage of NK release (Fig. 2J). The NK activity of memory-absent microrobots plateaued at 99.2 ± 16.4 U ml^−1^ after 12 hours, whereas the memory-equipped microrobots could continuously release NK for 72 hours, reaching 211.77 ± 26.83 U ml^−1^, which was 2.13-fold higher than the memory-absent microrobots (Fig. 2J). Overall, these microrobots exhibit superior onboard integration (Ni/Lc = 5.33; Ni refers to the number of integrated on-board functions, and Lc refers to the characteristic length of the microrobot) and high yield (3.32 × 10^7^ hours^−1^; 98.93 ± 0.68% biohybridization), enabling efficient fabrication and enhanced autonomous functionality, representing a notable improvement over conventional mechanical and hybrid microrobots (Fig. 2K and table S3) (*45*–*56*).

### Swarm formation and functional amplification

**Fig. 3.**
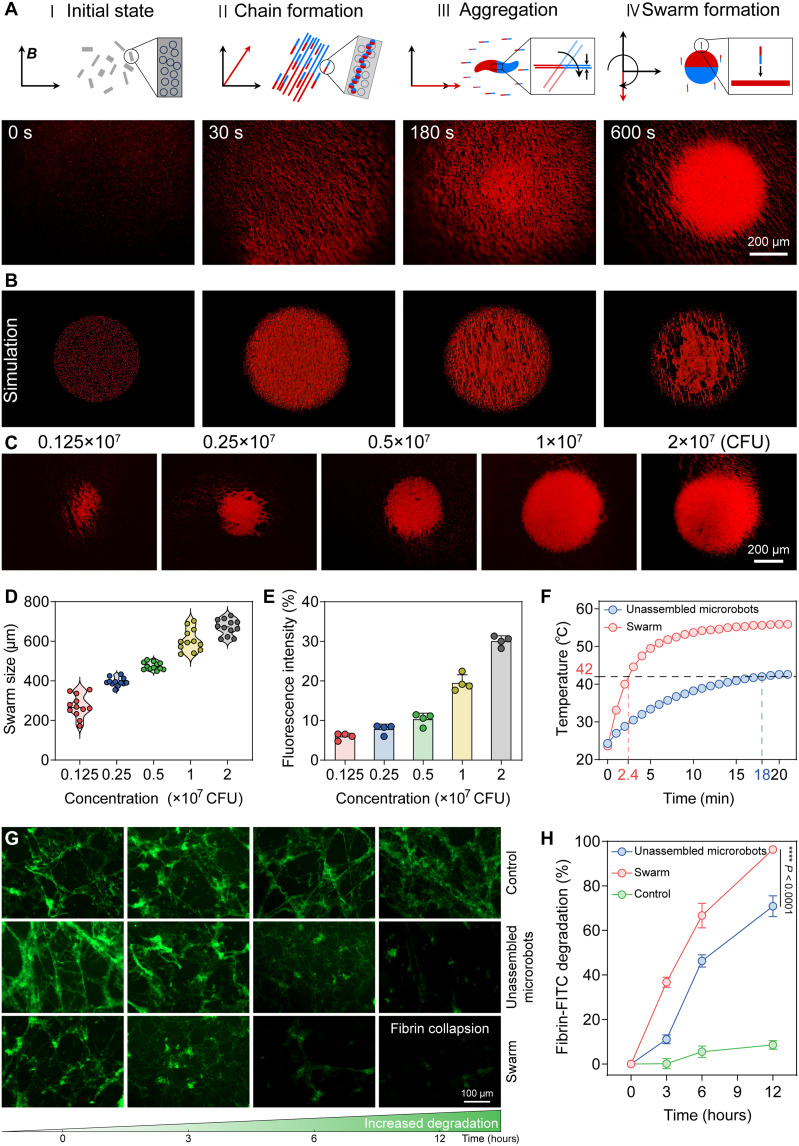
The formation of probiotic microrobot swarm and the amplification of their functions. (**A**) The schematic generation process of probiotic microrobot swarm. The rods illustrate probiotic microrobots chains, and the blue and red parts show the different magnetizations. The aggregation of the probiotic microrobot swarm under RMF was demonstrated in real experiments. (**B**) Aggregation of probiotic microrobot swarm under RMF in simulations. (**C**) Representative fluorescence imaging of the microrobot swarm with different input doses of microrobots. (**D**) Probiotic microrobot swarm size with various concentrations of microrobots under the same RMF (20 mT, 2 Hz, 10 min) (*n* = 12; means ± SD). (**E**) Quantitative fluorescence intensity of the probiotic microrobot swarm varied with input dose (*n* = 4; means ± SD). (**F**) Temperature change of unassembled microrobots and densely aggregated swarm over time under AMF (375 kHz, 330 Oe). (**G**) The quantitative comparison of fibrin degradation efficiency between unassembled microrobots and densely aggregated swarm at different time points (0, 3, 6, and 12 hours), showing amplification by dense aggregation and an autonomous effect of the memory module (*n* = 6; means ± SD). (**H**) Comparison of FITC-labeled fibrin clot degradation between unassembled microrobots and densely aggregated swarm using fluorescence imaging at 0, 3, 6, and 12 hours. Statistical analysis was performed using one-way analysis of variance (ANOVA) with Tukey’s post hoc test for (H).

To increase the concentration of tumor softening agents and locomotion performances, we applied a rotating magnetic field (RMF) to form a dense aggregation of the microrobots. Prior to applying the RMF treatment, the microrobots were self-assembled into clusters of an average diameter of 543.88 ± 11.24 μm under local magnetic interaction and surface tension (Fig. 3A). Supported by simulation results, we demonstrated that the entire aggregation process stemmed from the RMF-driven assembly of small chains (Fig. 3B and movie S1). Under the RMF, the microrobots transformed into chain formation and rotated with the external field. As the rotation continued, these chains progressively collapsed and formed a densely aggregated swarm (Fig. 3, A and B, and movie S1). In addition, the microrobot swarm size increased considerably from 271.61 ± 56.59 to 671.12 ± 40.43 μm as the dose rose from 0.125 × 10^7^ to 2 × 10^7^ colony-forming units (CFU) (Fig. 3, C and D). Similar to the microrobot swarm size, imaging area fluorescence was also continually enhanced by increasing the dose (Fig. 3, C and E). The morphology was effectively tuned by the precise control of probiotics dose, RMF treatment duration, and magnetic field frequency and intensity (figs. S9 to S11). Stronger magnetic fields also accelerated swarm formation (fig. S9). Higher RMF frequencies enhanced fluorescence intensity during 30 to 180 s but reduced swarm stability at ≥10 Hz (fig. S10).

Building on these aggregation characteristics, the probiotic microrobot swarm had a faster magnetothermal conversion capability at the same time points compared with unassembled microrobots. An ~2.6-fold increase in the specific absorption rate of probiotic microrobot swarm was obtained compared with the unassembled probiotic microrobots (fig. S12A). Under AMF stimulation at 42°C, the microrobot swarm activated the thermal sensor and memory function significantly faster than the dispersed microrobots, reducing the response time from 18 to 2.4 min (Fig. 3F and fig. S12, B and C). These results suggest that the swarm could enhance the magnetothermal conversion efficiency of probiotic microrobots.

Next, the fibrin degradation effects of microrobots in vitro were evaluated by fluorescein isothiocyanate (FITC)–labeled fibrin clots. Fluorescence imaging confirmed a stable cross-linked FITC-labeled fibrin network, with no significant intensity change observed from 0 to 12 hours. The intact fibrin skeletons were gradually degraded to filaments and showed collapsed structure with the treatment of unassembled microrobots group (Fig. 3G). These unassembled microrobots achieved a degradation efficiency of 70.69% at 12 hours, indicating a sustained effect. In contrast, the densely aggregated swarm group exhibited a 96% degradation efficiency (Fig. 3H), demonstrating efficient and sustained fibrin degradation by the swarm.

### Enhancing swarm mobility through 3D waving under the magnetic navigation

**Fig. 4.**
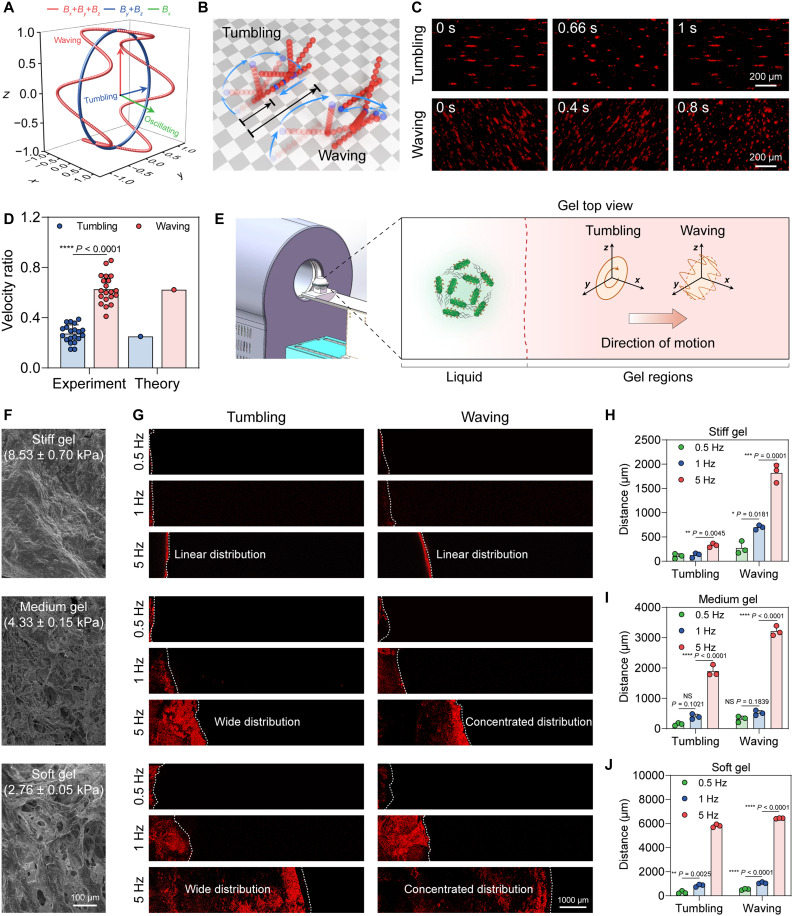
Comparison of the tumbling and waving motion capabilities of probiotic microrobot swarm. (**A**) Visualization of the superposition of the magnetic field, including an in-plane tumbling magnetic field (*B_y_* + *B_z_*) and an out-of-plane WMF (*B_y_* + *B_z_* + *B_x_*). (**B**) Schematic diagram of particle chain tumbling and waving under the applied magnetic field. To better illustrate the motion details, the schematic intentionally exaggerates the distance traveled while keeping the relative proportions unchanged. (**C**) Probiotic microrobot swarm performed tumbling and waving motion under magnetic field (8 mT, 0.5 Hz) manipulation. The complete corresponding simulation results were shown in fig. S17. (**D**) The comparison of experimental and theoretical velocity in tumbling mode, waving mode, and theoretical maximum (experiment: *n* = 20; means ± SD). (**E**) Schematic of a magnetic-field-actuated probiotic microrobot swarm penetrating collagen gels with different stiffnesses. (**F**) Characterization of the morphology of collagen gels with different stiffness by SEM imaging. (**G**) Comparison of the penetration depth of probiotic microrobot swarm in collagen gels under tumbling and waving locomotion with different frequencies (0.5, 1, and 5 Hz) through fluorescence imaging. (**H** to **J**) Quantitative comparison of the penetration depth of probiotic microrobots in collagen gels under tumbling and waving locomotion with different frequencies (0.5, 1, and 5 Hz) (*n* = 3; means ± SD). Statistical analysis was performed using one-way ANOVA with Tukey’s post hoc test for (D), (H), (I), and (J). Not significant (NS), *P* > 0.05.

Robust locomotion capability is a prerequisite for microrobots to navigate, penetrate, and perform therapeutic functions within the mechanically complex tumor microenvironment (*57*, *58*). To enable real-time visualization and actuation of probiotic microrobots, we developed a vision-based magnetic torque actuation system to achieve multimodal locomotion (figs. S13 and S14 and movies S2 to S5). The tumbling mode driven by periodic contact between the microrobots and the surface is highly inefficient, achieving only about 30% of the ideal pure-rolling speed (Fig. 4, B to D). Kinetic analysis identified slipping as the primary factor causing this reduction in velocity compared to pure rolling (fig. S15). Specifically, slipping occurs when the frictional force required to maintain pure rolling exceeds the available static frictional force (*f_r_* > μ*_s_F_n_*). Considering microscale interactions, the effective support force (*F_n_*) varies dynamically with the contact area, allowing microrobots to intermittently advance during tumbling cycles, but with greatly reduced average speed. To address this limitation, we introduced oscillations along a third axis, increasing frictional interaction by enlarging the effective contact area (Fig. 4A). This adaptation resulted in significant improvements in locomotion efficiency, with waving-mode microrobots advancing ~0.63 body lengths per cycle compared to 0.27 body lengths for tumbling-mode microrobots (Fig. 4D; fig. S16 and S17; and movies S2 and S4). The paddle-like waving mode increased frictional interactions and promoted rolling over slipping, achieving markedly higher velocities, especially in environments prone to slipping (Fig. 4, C and D, and fig. S15B).

Next, we evaluated the motility of probiotic microrobots in 3D viscoelastic and solid environments. To satisfy the application in vivo, we constructed a magnetic navigation setup with 30-cm operation space and 5-DOF (degree of freedom) manipulation (Fig. 4E and fig. S18). Collagen gels were prepared with variable stiffness to simulate tumor mechanical environment. The morphology and the porosity of collagen gels were characterized by SEM (Fig. 4F). The density and cross-link of collagen fiber were positively correlated with gel stiffness, while the porosity of collagen fiber gradually declined with gel stiffness. Mechanical characterizations showed that the compression moduli of collagen gels were 8.53 ± 0.7 kPa (stiff; consistent with tumor tissue), 4.33 ± 0.15 kPa (medium), and 2.76 ± 0.05 kPa (soft; represents tumor tissues with reduced mechanical moduli), respectively (Fig. 4F).

Subsequently, the gel penetration performance of densely aggregated swarm was investigated under magnetic navigation. An RMF in the *x*-*y* plane was applied to control the densely aggregated swarm formation, and tumbling and waving mode with different frequencies (0.5, 1, and 5 Hz) were applied for gel penetration, respectively. Regardless of whether with the tumbling or waving mode locomotion, the penetration depth of the probiotic microrobot swarm in gels with different stiffness was gradually enhanced with the increase of magnetic field frequencies, reaching the maximum depth at 5 Hz. The LSCM images displayed a limited locomotion of the microrobots in the stiff gel with tumbling motion (Fig. 4G). Fluorescence analysis showed that the microrobots were located at a position of 327.48 ± 40.93 μm in the stiff gel with a velocity of 0.02 ± 0.08 μm s^−1^ (Fig. 4H and fig. S19). By contrast, microrobots actuated with waving motion exhibited significantly improved penetration, achieving a depth of 1820.51 ± 184.89 μm, with a ~5.55 times higher velocity. Similarly, the waving motion resulted in 1.69-fold and 1.10-fold increases in penetration depth in medium and soft gels, respectively. This penetration ability was consistently enhanced as gel stiffness decreased for both tumbling and waving motions (Fig. 4, I and J). In the soft and medium gels, the waving motion of the probiotic microrobot swarm exhibited a concentrated penetration pattern compared to the dispersed distribution observed with tumbling motion (Fig. 4G). In addition, no probiotic microrobots were detected in the stiff or medium gels without magnetic fields, while only negligible penetration of the microrobots was observed in the soft gel due to its loose porosity (fig. S20). These results demonstrated that waving motion had the unique advantage in microrobot penetration in collagen gels compared to tumbling motion, particularly in 3D viscoelastic matrices.

### Sustainable antitumor mechanoregulation via the microrobot swarm

**Fig. 5.**
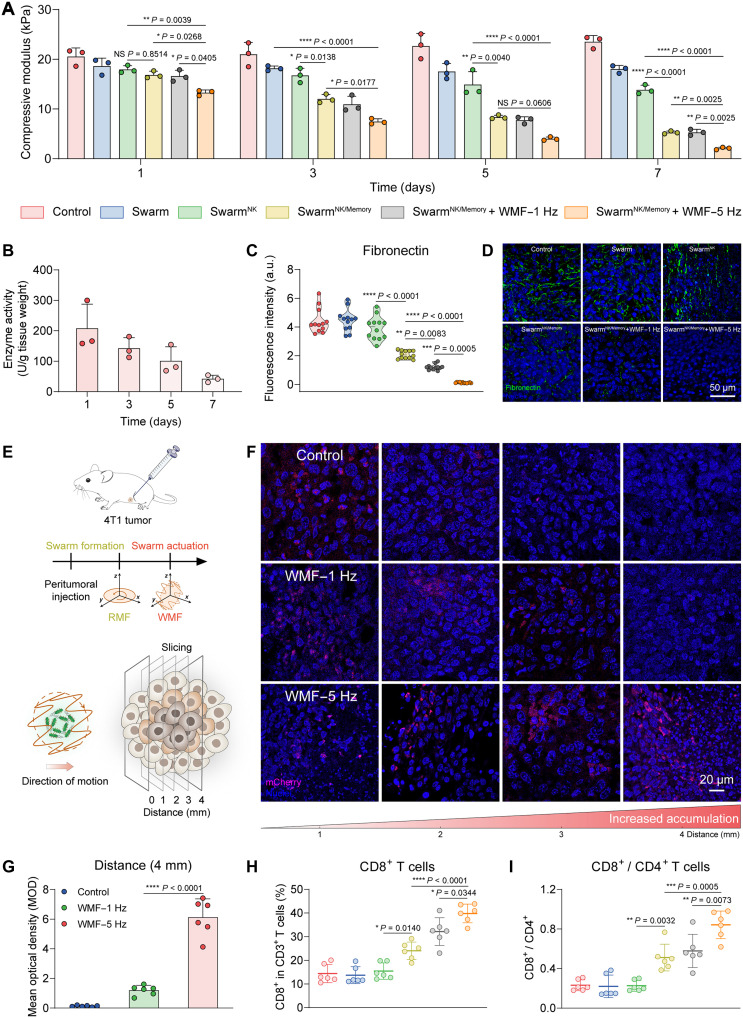
Tumor mechanical microenvironment regulation of the probiotic microrobot swarm. (**A**) Dynamic compression moduli of 4T1 tumor tissues after 1, 3, 5, and 7 consecutive days of different treatments, respectively (*n* = 3; means ± SD). (**B**) The quantification of NK activity in tumor tissues of mice at different time points after intratumoral injection of microrobots. (**C**) Quantification of fibronectin in fluorescence intensity after different treatments (*n* = 12; means ± SD). (**D**) Tumor tissues were excised after different treatments and analyzed for the related fibronectin expression level through immunofluorescence staining (green, fibronectin; blue, cell nuclei). (**E**) Schematic of the microrobot swarm tumor tissue penetration driven by WMF (74 mT, 60 min). (**F**) Average distribution of microrobots in transverse tumor sections visualized at 1, 2, 3, and 4 mm from the peritumoral injection site (red, microrobots; blue, cell nuclei). (**G**) The quantitative fluorescence analysis of microrobots at 4-mm tumoral depths (*n* = 6; means ± SEM). (**H** and **I**) The CD8^+^ cells in CD3^+^ T cells and the ratio of CD8^+^ to CD4^+^ T cells in 4T1 tumor tissues were analyzed by flow cytometry (*n* = 6; means ± SD). Statistical analysis was performed using one-way ANOVA with Tukey’s post hoc test for (A), (C), (G), (H), and (I). NS, *P* > 0.05.

Motivated by the remarkable penetration and fibrin degradation capability of the probiotic microrobot swarm in vitro, the antitumor mechanoregulation of microrobot swarm in vivo was investigated. Taking triple-negative breast cancer (TNBC) as an example, the tumor tissues have higher extracellular matrix (ECM) stiffness compared to normal breast tissues (*59*, *60*). 4T1 TNBC model was established to assess the antitumor mechanoregulation effect of probiotic microrobots. The mechanical stiffness dynamics of 4T1 tumor tissue were assessed at 1, 3, 5, and 7 days posttreatment. The compressive modulus of tumors in the Swarm^NK/Memory^ group continuously decreased over time, reaching 5.26 ± 0.3 kPa at day 7. The compressive moduli in the Swarm^NK/Memory^ + WMF (waving magnetic field)–5 Hz group significantly decreased to 2.06 ± 0.25 kPa, which was 11.38-fold lower compared to the control group (Fig. 5A). In addition, with the continuous regulation of tumor mechanics, the NK released by the probiotic microrobot swarm in situ within the tumor decreased by 79.64% from day 1 to day 7 (Fig. 5B). Immunofluorescence staining of tumor tissue sections further revealed that the relative fibronectin fluorescence intensity in the Swarm^NK/Memory^ + WMF group decreased by 97.26% compared to the control group (Fig. 5, C and D). Consistently, the α-SMA (α-smooth muscle actin) and collagen I were significantly attenuated in the Swarm^NK/Memory^ and Swarm^NK/Memory^ + WMF group (fig. S21, A to D).

The antitumor mechanoregulation mechanism of the swarm was further investigated. Peritumoral administration of probiotic microrobots was performed, followed by the application of RMF in the *x*-*y* plane to manipulate swarm formation (fig. S21E). Subsequently, the WMF was applied to drive the microrobot swarm with waving motion for tumor penetration (Fig. 5E). To assess the spatial distribution of probiotic microrobots inside tumors, we conducted longitudinal histological sectioning at 1-mm-depth intervals (Fig. 5F). At a 4-mm depth, the mean fluorescence intensity of consecutive transverse tumor sections in the WMF–5 Hz group was 5.08-fold higher than in the WMF–1 Hz group (Fig. 5G), suggesting that deep tumor penetration could be realized via the 3D waving locomotion. To further verify microrobot swarm behavior during in vivo actuation, we performed magnetic resonance imaging (MRI) immediately after injection and magnetic actuation. It showed a consistent intratumoral T2 signal, indicating that the microrobot swarm maintained its structural integrity during 3D waving locomotion. Although the heterogeneous tumor microenvironment may partially disperse the swarm into smaller subclusters, such fragmentation does not diminish their penetration capability, as the resulting clusters remain within the effective propulsion range (figs. S22 and S23E). Because tissue softening and microrobot penetration occur on different temporal scales, we combined shear wave elastography (SWE)–based stiffness mapping with MRI and in vivo fluorescence imaging to capture their coordinated evolution (figs. S23 and S24). These temporally aligned multimodal results demonstrate that localized softening facilitates deeper microrobot penetration in vivo. Together with the in vitro results, a reciprocal softening-penetration feedback loop was constructed via the microrobots to break down the tumor ECM barrier.

As the stiffness of ECM was attenuated, the penetration of cytotoxic T lymphocytes (CD8^+^ T cells) and helper T cells (CD4^+^ T cells) in tumor tissues was significantly enhanced (fig. S25). Flow cytometry analysis revealed an obvious increase in the proportions of CD8^+^ T cells in both the Swarm^NK/Memory^ and Swarm^NK/Memory^ + WMF groups relative to the Swarm^NK^ group. Notably, the Swarm^NK/Memory^ + WMF group exhibited the highest proportions of CD8^+^ in CD3^+^ T cells (39.82 ± 3.93%) and the highest CD8^+^/CD4^+^ T cell ratio (0.66 ± 0.08), which were substantially higher than those observed in the Swarm^NK^ group (15.48 ± 3.47% CD8^+^ T cells and 0.22 ± 0.05 CD8^+^/CD4^+^ T cell ratio) (Fig. 5, H and I). These results demonstrated that the microrobots with memory and waving locomotion could effectively facilitate the cytotoxic T cell intratumoral infiltration by autonomous tumor stiffness regulation, which held promise for enhancing the efficacy of tumor immunotherapy.

### Antitumor efficacy of the microrobot swarm

**Fig. 6.**
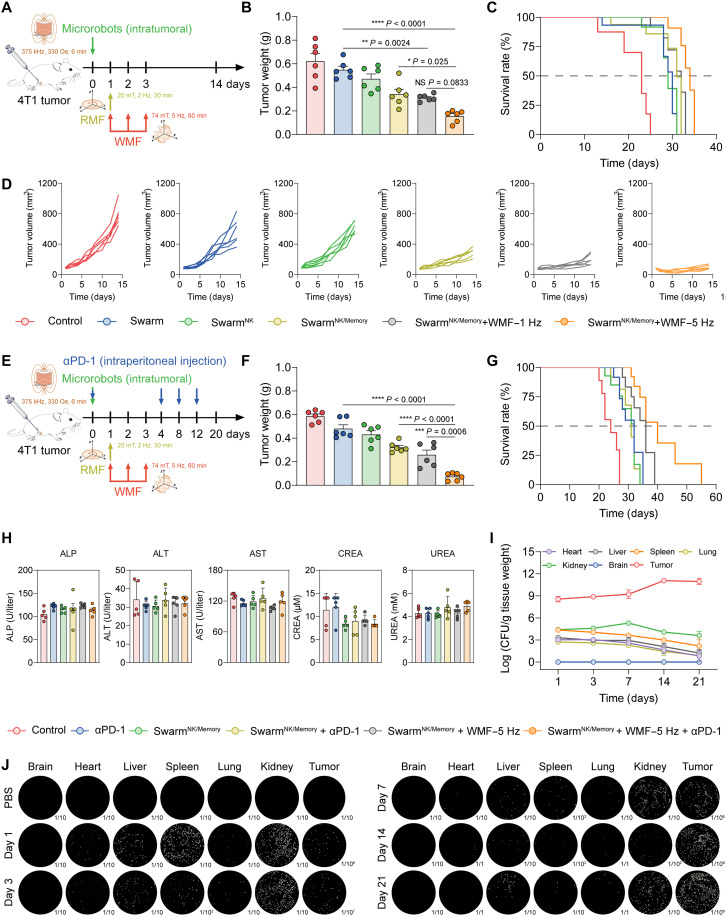
Antitumor efficacy of the memory-encoded microrobot swarm. (**A**) Timeline of probiotic microrobot swarm treatments. (**B**) Average tumor weight in different treatment groups of BALB/c mouse model (*n* = 6; means ± SEM). (**C**) Overall survival time of BALB/c mice in different treatment groups (*n* = 6). (**D**) Growth curves of tumors in each group (*n* = 6; means ± SEM). (**E**) Schematic illustration of probiotic microrobot swarm in combination with αPD-1 for 4T1 tumor therapy. The αPD-1 was injected intraperitoneally at a dose of 100 μg per mouse every 4 days, for a total of four injections. (**F**) Tumor weight of every BALB/c mouse with different treatment strategies (*n* = 6; means ± SEM). (**G**) Overall survival time of BALB/c mice in different treatment groups (*n* = 6). (**H**) The blood biochemical indices of mice were measured on day 20. The blood biochemical indices of liver function markers included ALP, ALT, and AST; kidney function markers included CREA and UREA (*n* = 5; means ± SEM). (**I**) Representative photographs of LB agar plates spread with tissue homogenate of tumors and major organs from 4T1-bearing mice at different time points after intratumoral injection of probiotic microrobots (*n* = 3; means ± SD). (**J**) The quantification of probiotics colonization in major organs and tumor tissues of mice at different time points after intratumoral injection of microrobots. Statistical analysis was performed using one-way ANOVA with Tukey’s post hoc test for (B) and (F). NS, *P* > 0.05.

The antitumor efficacy of the microrobot swarm was further studied as illustrated in Fig. 6A. Compared to the control groups, the Swarm^NK/Memory^ under the WMF treatment demonstrated an efficient antitumor effect (Fig. 6, B and D, and fig. S26, C and D). Microrobot swarms actuated at WMF–5 Hz achieved 87.52% tumor growth inhibition, which was higher compared to those at WMF–1 Hz (74.67% tumor growth inhibition). Consistently, the tumor-bearing mice in the Swarm^NK/Memory^ group showed the longest median survival time (Fig. 6C). Beyond comparing magnetic actuation modes, we further benchmarked the therapeutic efficacy against conventional chemotherapy.WMF-actuated microrobot swarms achieved enhanced tumor inhibition compared with static microrobots and better efficacy comparable to free doxorubicin (Dox), while their combination with Dox produced the strongest antitumor response (fig. S27). Subsequently, the anti-PD-1 antibody (αPD-1) for robust T cell responses was used together with probiotic microrobots (Fig. 6E). As expected, αPD-1 monotherapy only exhibited a modest inhibitory effect on tumor growth. The combination of Swarm^NK/Memory^ + WMF therapy with αPD-1 demonstrated the most potent antitumor efficacy by day 20 (Fig. 6F and fig. S26, G to I). These findings indicate that the ECM stiffness regulation strategy of probiotic microrobots synergized with immune checkpoint blockade therapy, enhancing tumor suppression. Notably, the Swarm^NK/Memory^ + WMF combination with αPD-1 markedly extended the median survival time of mice over 36 days, which achieved the best therapy compared to other treatment groups (Fig. 6G). Accordingly, we extended our study to a pancreatic cancer (Panc02) model, which features a dense and fibrotic ECM environment. Notably, the microrobot swarm with WMF actuation therapy effectively reduced the tumor elastic modulus and suppressed tumor growth (fig. S28). Collectively, these results proved that microrobot swarm could elicit long-term antitumor effects through its memory function and 3D waving motion of microrobots.

Last, the safety profile of the probiotic microrobots therapy platform was evaluated. The body weights of mice in each group remained stable throughout the treatment period (fig. S26, B and F). After administration for 20 days, the blood chemistry analysis of liver and kidney function markers was carried out. Alanine aminotransferase (ALT), aspartate aminotransferase (AST), alkaline phosphatase (ALP), creatinine (CREA), and urea nitrogen (UREA) remained at normal levels postinjection of microrobots (Fig. 6H and figs. S29 and S31). In further assessing the in vivo fate and clearance of magnetic components of the microrobots, iron quantification on day 21 revealed that MNPs remained predominantly within tumors after intratumoral administration, with minimal accumulation in major organs or blood. A notable increase in splenic iron indicated that the spleen served as the primary site for nanoparticle processing and clearance (figs. S30 and S31). In parallel, we examined the biodistribution and clearance of the probiotic component of the microrobots. Probiotics proliferation quantification showed that probiotics were gradually cleared from major organs, including the heart, liver, spleen, lung, and kidney (Fig. 6, I and J). In sharp contrast, the CFU in tumors increased exponentially over time, indicating that the probiotics preferentially colonized the tumor without imposing a burden on major organs. We further monitored the persistence of the engineered genetic reporter in vivo. In vivo fluorescence imaging demonstrated that mCherry signals remained detectable in tumors for at least 12 days and were confirmed by ex vivo analysis, with only minor liver and kidney enrichment and no significant accumulation in other organs (fig. S24). Hematoxylin and eosin analysis revealed no distinct pathological abnormalities in major organs (figs. S32 and S34). Prussian blue (PB) staining showed that microrobots barely accumulated in the other organs except for the spleen (figs. S33 and S35). All these findings demonstrated the favorable biosafety profile of the probiotic microrobots therapy platform.

## DISCUSSION

Patients with solid tumors require long-term treatment that could take weeks or even months. With conventional therapies, frequent clinical visits are inevitable, which brings substantial inconvenience to the patients. Developing microrobots that autonomously operate for sustained treatment offers a promising avenue to alleviate this dependency on continuous hospital-based care. Despite recent advancements in microrobotics, state-of-the-art systems largely rely on persistent external control signals, primarily due to an intrinsic lack of integrated sensing and memory capabilities. While a variety of physical memory materials (Ag/SiO_2_/Au, gold nanoparticles/hydrogel, NiTi/NiTiCu, liquid crystal elastomers, etc.) are available (table S1), they inherently struggle to meet the combined requirements of biocompatibility, size, memory, and stability for antitumor treatment. In contrast, genetically encoded memory-capable biocircuits offer distinct advantages on biocompatibility, heritability, dynamic responsiveness, and programmability, rendering them ideal for complex biomedical applications. The key lies in synergizing the memory-capable module with other functional components in the biological robotic circuit to have a precise control over the therapeutic process, combining efficacy, autonomy, and biosafety. In this work, we engineered genetic memory into magnetic probiotic microrobots for autonomous antitumor mechanoregulation. The memory circuit Bxb1-ssrA-*attB*-P7-*attP* was integrated with the thermal sensor (TcI) and the actuator (pR-pL) to control the autonomous NK release and achieve sustained mechanoregulation. It circumvented the problem that the TcI alone lacked the ability to retain transient signals and therefore compromised the therapeutic effect.

In addition to the engineered biocircuit, we also used magnetically controlled 3D locomotion to synergistically enhance the tumor tissue penetration. Conjugated MNPs served dual roles as sensors and transducers, converting magnetic signals into thermal and mechanical energy. Through precise manipulation of actuating magnetic fields, diverse locomotion modes including tumbling, oscillating, and waving were achieved. While commonly used for moving in specific direction, its efficiency in tumbling is notably limited by significant slipping in fluid environments, resulting in low velocities (~0.27 body lengths per second). To overcome this limitation, we introduced a 3D waving mode. Previous studies have demonstrated that WMFs can effectively induce complex locomotion behaviors on uneven surfaces, such as the disassembly and dispersion of MNP clusters (*61*). Consistently, our results revealed that the implementation of waving mode nearly doubled microrobot velocity, reaching ~0.62 body lengths per second. This substantial improvement arises from enhanced frictional interactions, favoring rolling over slipping, particularly during critical high velocity periods. Moreover, stiff and densely cross-linked collagen gels, structurally analogous to tumor tissues, provide increased opportunities for interactions during 3D locomotion, further enhancing the microrobots’ mobility in unstructured environments. Such advantages underscore the potential of waving-mode microrobots for deep tissue infiltration, ultimately facilitating more effective therapeutic interventions. To translate these locomotion advantages into in vivo applications, it is essential to ensure that sufficient magnetic torque can be delivered through biologically relevant tissue thicknesses (*62*–*66*). Although magnetic field attenuation limits the penetration depth of actuation in biological tissues (table S4), our 5-DOF magnetic control system effectively propels our probiotic microrobots. By integrating permanent and electromagnetic actuation, the system maintained stable torque transmission through the 4.46-cm porcine tissue, comparable to the typical human abdominal thickness. This result suggests that the approach could hold promise for noninvasive magnetic control of microrobots in deep-seated organs, providing a scalable foundation for future translational studies.

The genetically encoded memory biocircuit is activated by a one-time magnetic hyperthermia, triggering sustained NK release for fibrin degradation and ECM softening. Simultaneously, magnetically controlled 3D wave-like locomotion enhances the deep tumor penetration of microrobots. With the design of genetic biocircuit and a magnetic field–driven 3D waving mode, our microrobots achieved a softening-penetration positive feedback loop. The memory-encoded ECM softening significantly enhances microrobot propulsion under a 3D oscillatory magnetic field. This reduction in stiffness, in turn, facilitates deeper tissue penetration, forming a self-reinforcing cycle. Such a positive feedback mechanism overcomes the limitations of conventional passive drug delivery in solid tumor treatment, enabling deeper antitumor modulation and enhanced immune activation. By breaching the ECM barrier, the tumor stiffness could be attenuated to 2.06 kPa in the TNBC model, facilitating the immune cell and drug penetration into deep tumor tissues. CD8^+^ T cells and PD-1 infiltration within the tumor microenvironment were boosted, thereby augmenting immune responses against solid tumors. Notably, recent studies showed that increased ECM stiffness in solid tumors could exacerbate CD8^+^ T cell exhaustion within the tumor microenvironment (*67*). The enhanced antitumor effect in our study strongly suggests that reshaping the ECM could offer a distinctive strategy for modulating the immunosuppressive tumor microenvironment. In addition, biosafety evaluations showed that the probiotic microrobots had no significant effects on body weight or blood chemistry in mice, confirming their favorable safety profile. Beyond general biosafety profiles, potential concerns regarding bacterial horizontal gene transfer must also be considered for translational applications. The ratio of bacteria to human somatic cells in the body is ~1:0.76, and bacteria are ubiquitous in the human environment (*68*). Despite this ubiquity, whether bacteria could induce horizontal gene transfer to human somatic cells remains unclear, largely due to limitations in detection technologies (*69*). To achieve such transfer, bacterial genetic fragments must first overcome multiple barriers, including the cell membrane, nuclear envelope, and nucleases. Even if these barriers are bypassed and the fragments reach the nuclear region, additional challenges remain. Bacterial genes would need to acquire appropriate regulatory elements such as introns, promoters, and terminators (*70*). In addition, they need to establish productive interactions with existing host genes before functioning within eukaryotic cells. In this study, the injected bacterial dose was ~0.1% of the total bacterial load in the animal body, which was a negligible fraction relative to the endogenous microbiota. Together, the horizontal gene transfer induced by the administered bacteria could be minimized by controlling the quantity of bacteria introduced in vivo.

In addition to biosafety considerations, successful clinical translation also requires selecting appropriate administration routes for different tumor types and anatomical contexts (*71*–*78*). When scaling to human-relevant tumor volumes, the choice of administration route must be tailored to the tumor type and clinical context. Larger and more heterogeneous tumors in patients present additional barriers such as poor vascularization, dense ECM, and limited penetration depth, which complicate uniform distribution of therapeutic microrobots. To address these issues, alternative administration routes can be adapted to specific tumor types (table S5). For colorectal or gastric cancers, oral administration enables gastrointestinal colonization, thereby eliciting sustained local and systemic effects. However, bacterial viability is challenged by harsh pH conditions and competition with commensal microbiota, which necessitates protective encapsulation or other engineering strategies. For deep-seated or inaccessible tumors, intravenous injection allows rapid tumor access via the leaky tumor vasculature, which has to face the challenges such as rapid clearance by the mononuclear phagocytic system and systemic toxicity, requiring genetic modifications to balance safety and efficacy. For solid tumors (e.g., breast cancer and melanoma), intratumoral injection achieves high local bacterial concentrations and bypasses vascular and systemic barriers, but it requires precise containment and image-guided procedures (*29*, *30*).

Looking ahead, the genetically encoded biocircuit provides a general template for medical microrobot design. Microrobots incorporating NK therapeutic modules exhibit potential not only for antitumor treatments but also for addressing fibrotic conditions such as thrombosis, atherosclerosis, pulmonary fibrosis, and hepatic fibrosis. Future research should systematically optimize memory biocircuit, including enhancing storage capacity and encoding diversified disease-specific information. Such advancements will be instrumental in broadening the therapeutic applicability of microrobotic platforms, paving the way for the development of autonomous treatments.

## MATERIALS AND METHODS

### Materials

EcN was obtained from Beijing Biobw Bioscience and Technology Co., Ltd. TNBC (4T1; American Type Culture Collection, CRL-2539) was purchased from Procell Life Science and Technology Co., Ltd. All gene sequences were synthesized by Jinweizhi Biotechnology Co., Ltd. (Suzhou, China). Iron (III) perchlorate sodium dihydrogen phosphate [Fe(ClO_4_)_3_·6H_2_O], tetraethyl orthosilicate (TEOS), sodium dihydrogen phosphate (NaH_2_PO_4_), urea (NH_2_CONH_2_), cetyltrimethylammonium bromide (CTAB), sodium salicylate (NaSal), triethanolamine (TEA), fibrinogen from human plasma, succinyl-Ala-Ala-Pro-Phe-*p*-nitroanilide, *p*-nitroaniline, and thrombin from human plasma (250 U) were all purchased from Sigma-Aldrich. Collagen type I, rat tail was obtained from Corning. Fibrinogen from human plasma was acquired from Thermo Fisher Scientific. Anti-mouse PD-1 (CD279), clone J43 was acquired from BioXCell. Anti-fibronectin antibody (Abcam, 268020), goat anti-rabbit immunoglobulin G (IgG) H&L Alexa Fluor 488 (Abcam, 150077), and goat anti-rabbit IgG H&L Alexa Fluor 647 (Abcam, 150079) were all purchased from Abcam. Cell Carrier Spheroid ULA 96-well microplates were purchased from PerkinElmer. μ-Slide 8 well uncoated was acquired from ibidi.

### Preparation of MNPs, MNPs@APTES, and MNPs@FITC

For the synthesis of spindle cores (α-Fe_2_O_3_), Fe(ClO_4_)_3_·6H_2_O (6.5 mmol), NaH_2_PO_4_ (0.15 mmol), and NH_2_CONH_2_ (5 mmol) were well dispersed in deionized water (50 ml of ddH_2_O). The solution was treated in an oil bath (100°C, 24 hours) in a triangular flask. The α-Fe_2_O_3_ was separated from the dispersion by centrifugation (9500 rpm) after the solution cooled down naturally (*79*). The α-Fe_2_O_3_@SiO_2_ was prepared by first dissolving TEA (68 mg), α-Fe_2_O_3_ (320 mg), CTAB (380 mg), and NaSal (126 mg) in 25 ml of deionized water with vigorous stirring at 80°C for 3 hours. TEOS (3.6 ml) was then added, and the reaction continued at 80°C for 45 min. The products were isolated by centrifugation (9500 rpm, 15 min) and washed six times with ethanol. For the synthesis of Fe_3_O_4_@SiO_2_, residual surfactant in α-Fe_2_O_3_@SiO_2_ was removed by calcination in air at 550°C for 5 hours. Then, α-Fe_2_O_3_@SiO_2_ was annealed in H_2_/Ar atmosphere (450°C, 3 hours) to obtain Fe_3_O_4_@SiO_2_. For the synthesis of Fe_3_O_4_ (MNPs), Fe_3_O_4_@SiO_2_ (20 mg) was added to NaOH (0.1 mol, 50 ml) with sonication for 5 hours to remove the silica shell. For the synthesis of MNPs@APTES (3-aminopropyltriethoxysilane), MNPs (10 mg) were well dispersed in 20 ml of isopropanol. Aqueous ammonia (500 μl) and APTES (200 μl) were added to the solution and vigorously stirred in Ar atmosphere under 35°C for 3 hours. Then, the solution was vigorously stirred in Ar atmosphere (80°C, 8 hours). MNPs@APTES were collected by magnetic separation and washed with ddH_2_O for three times. For the synthesis of MNPs@FITC, FITC (1 mg) was added to 1 ml of ethanol solution. Then, the FITC solution was well dispersed in solution containing MNPs (1.5 mg), 1-ethyl-3-(3-dimethylaminopropyl)carbodiimide (EDC; 100 mg), *N*-hydroxysuccinimide (NHS; 66 mg), and 10 ml of phosphate-buffered saline (PBS) (pH 5.5) with sonication for 2 hours. MNPs@FITC were collected by magnetic separation and washed with ethanol for three times to remove residual FITC.

### Fabrication of the microrobots

For the probiotics culture, EcN was cultured in lysogeny broth (LB) medium containing tryptone (1 g), NaCl (1 g), and 0.5 g of yeast extract powder per liter of ddH_2_O. For the preparation of highly efficient competent cells, EcN stored in an ultralow-temperature freezer was streaked on an LB plate and incubated at 37°C for 12 hours. A single colony was selected, inoculated into 5 ml of LB medium, and incubated in a shaker (37°C, 220 rpm, 12 hours).

The activated probiotics solution (500 μl) was inoculated into LB (50 ml) and oscillated (37°C, 220 rpm) until the solution reached an optical density at 600 nm of 0.4. The competent cells were collected by centrifugation (4000 rpm, 10 min) and washed three times with glycerol. For plasmid construction and molecular biology, the gene sequences of sensor module (TcI), actuator module (pR-pL), and memory module (Bxb1-ssrA-*attB*-P7-*attP*) were synthesized by Jinweizhi Biotechnology Co., Ltd. (Suzhou, China) and integrated into plasmid pBV220 to construct engineered genetic circuits. The NK gene *aprN* (*pre-pro-nk*, National Center for Biotechnology Information GenBank: S51909) was obtained from the *Bacillus subtilis* var. Codon optimization strategy was applied to improve the expression of heterologous protein. Subsequently, the gene sequences of mCherry and *aprN* were integrated into engineered genetic circuits. Sanger sequencing and double digests were applied to verify the engineered genetic circuits. For the preparation of microrobots, engineered genetic circuits were transformed into EcN to construct engineered EcN. Amino-functionalized MNPs (MNPs@APTES) were linked on the surface of EcN by amide condensation. Engineered EcN was dispersed in PBS solution with the concentration of 10^8^ CFU ml^−1^. MNPs@APTES (200 μg) were added to the engineered EcN suspension together with the EDC (0.55 mg) and NHS (0.65 mg). Then, the solution was stirred at room temperature (100 rpm, 3 hours). The microrobots were obtained by differential centrifugation (3000 rpm, 5 min) and further separated by magnetic separation.

### Characterization of microrobots

SEM imaging, fluorescence imaging, flow cytometry, and vibrating sample magnetometer were used to characterize the microrobots. SEM (ZEISS GeminiSEM 300, Germany) was used to visualize the morphology of the microrobots. For visualization purposes, SEM images were pseudocolored with Adobe Photoshop software. Vibrating sample magnetometer (LakeShore7404, USA) was used to obtain the magnetic hysteresis curve of microrobots. To confirm the binding of MNPs to the surface of microrobots, the MNPs were labeled with FITC. Fluorescence images were captured by fluorescence microscopy (ZEISS LSM 880, Germany) with two fluorescence channels, green fluorescent protein and Cy5, which corresponded to the autofluorescence of FITC-labeled MNPs and probiotics. BD FACSAria III flow cytometer was applied to validate quantitatively the attachment of MNPs on microrobots.

### Magnetic control platform

The AMF (SPG-06A-II, Shenzhen Shuangping Power Technology Co., Ltd.) was used to measure magnetothermal effect. The temperature of probiotic microrobots was measured with a fiber optic temperature converter.

For the magnetic torque actuation and visualization system, a laboratory PC (personal computer) worked as the host computer for the high-level human-computer interface and visualization of data. A joystick was invoked as input equipment for manual trajectory control. LabVIEW (National Instruments, Inc.) was used to realize the preparation of relevant algorithms and control programs. Driving signals were generated by a high-speed DAQ (data acquisition) card and then amplified by the voltage amplifier before entering the coils. An optical microscope (Nikon, ECLIPSE Ts2R) was used to observe the real-time position and state of the microrobots to feedback for controllers. The motion behavior of microrobots or swarm was analyzed with ImageJ software.

The primates-scale magnetic control setup consisted of four main components including an array of permanent magnets, electromagnetic coils, water chiller, and control system. An array of permanent magnets was used to generate the RMF, and a set of electromagnetic coils were applied to produce axial oscillation.

### Magnetic particle simulation

A discrete element method combined with a hybrid potential function was used to accurately model the dynamic behavior of magnetic microrobots. Each microrobot was represented as a discrete particle with specific mass and shape characteristics. Contact forces and mechanical interactions between particles adopt *lj* potential to simulate particle behaviors in physiological conditions. The interactions between particles and boundary walls were modeled using Hertzian contact theory to realistically capture collisions, friction, and rebounding behaviors. In addition, viscous forces were included to simulate fluid resistance effects. The motion of a single magnetic particle subjected to external forces is calculated by

$$F_{B}=\left(m\cdot \nabla \right)B$$ (1)

where **F***_B_* denotes the magnetic force, **m***_d_* is the dipole moment, and **B** represents the magnetic field. When multiple particles approach each other, dipole-dipole interactions become significant, introducing additional forces (**F***_ij_*) and torques (**τ***_ij_*), described by

$$\begin{array}{c} F_{\mathrm{ij}}=\frac{3\mu_{0}}{4\pi r^{5}}\left[\left(m_{i}\cdot r_{\mathrm{ij}}\right)m_{j}+\left(m_{j}\cdot r_{\mathrm{ij}}\right)m_{i}+\left(m_{i}\cdot m_{j}\right)r_{\mathrm{ij}}-\frac{5\left(m_{i}\cdot r_{\mathrm{ij}}\right)\left(m_{j}\cdot r_{\mathrm{ij}}\right)}{r^{2}}r_{\mathrm{ij}}\right]\\ \tau_{\mathrm{ij}}=\frac{\mu_{0}}{4\pi r^{3}}\left[-\left(m_{i}\times m_{j}\right)+\frac{3}{r^{2}}\left(m_{j}\cdot r_{\mathrm{ij}}\right)\left(m_{i}\times r_{\mathrm{ij}}\right)\right] \end{array}$$ (2)

where **m***_i_* and **m***_j_* represent the dipole moment vectors of the interacting particles, and the vector **r***_ij_* = **R***_i_* − **R***_j_* denotes the separation vector between particle centers.

Dimensionless units used in the simulations are defined as follows: σ (particle radius) as the length unit, *m* (particle mass) as the mass unit, τ as the time unit, and ε as the energy unit. We fixed the length σ = 1, the mass *m* = 1, and the energy ε = 1. The simulations were carried out using the parameters *m_d_* = 1.02 and *B* = 55.4. The elastic constant is 10,000 for normal contact *K_n_* and 2857 for tangential contact *K_t_*. The velocity-Verlet algorithm was used for numerical time integration, with a time step of Δ*t* = 0.01τ to ensure enough time step to catch particle behaviors. Simulations were performed under constant number-volume-energy conditions, updating positions and velocities consistently over a total simulation duration of 1500,000τ. These computational simulations were conducted using the Large-Scale Atomic/Molecular Massively Parallel Simulator (LAMMPS) package and visualized using OVITO (*80*, *81*).

### Microrobots viability study

CFU assay was used to evaluate the viability of the microrobots. The microrobots were collected by magnetic separation and resuspended in PBS (1 ml) after fabrication. Then, probiotic microrobots were diluted 1:10^6^ (v/v) and 1:10^7^ (v/v) and seeded on agar plates, and the number of colonies formed was counted.

### Formation of the probiotic microrobot swarm

Probiotic microrobots (10^7^ CFU) were well dispersed in simulated body fluid (100 μl). Then, the solution was added to CellCarrier Spheroid ULA 96-well Microplates and assembled with the application of RMF in the *x*-*y* plane.

### Penetration of collagen gels with probiotic microrobots

To prepare collagen gels, collagen I rat tail (3.54 mg ml^−1^, 3.531 ml) was mixed with 500 μl of PBS (10×), and 969 μl of ddH_2_O was added. The stiffness of collagen gels was modulated by adjusting the pH of the collagen mixtures during polymerization (*18*). Then, the collagen mixtures were kept on ice, and 1 M NaOH or HCl was used to adjust the pH. The pH values of collagen mixtures were adjusted to 11.5 (stiff), 9.5 (medium), and 7.5 (soft), respectively. For the measurement of collagen gel stiffness, subsequently, the collagen mixtures (1 ml per well) were added into Φ 6.5 mm dish and incubated in a temperature-controlled incubator (37°C, 30 min) for polymerization. An electronic universal testing machine (UTM2502, Shenzhen Suns Technology Co., Ltd.) was used to measure the compressive modulus properties of collagen gels. For the compressive test, the tumor tissues were tested at a speed of 5 mm min^−1^ with 50% strain. For the penetration of collagen gels, subsequently, the collagen mixtures (200 μl per well) were added into chambered coverslip (μ-Slide 8 Well, uncoated, ibidi) and incubated in a temperature-controlled incubator (37°C, 30 min) for polymerization. The collagen mixtures took up two-thirds of the dish, and the rest of the dish was filled with PBS (1×, pH 7.4). The probiotic microrobots were added to the PBS solution, and a magnetic field was performed to actuate the microrobot swarm for collagen gel penetration with multimodal motion.

### Fibrinolytic activity and ability assay

The fibrinolytic activity of NK was evaluated using the conventional fibrin plate assay, using urokinase as the reference standard. Agarose (1 g) was dissolved in 50 ml of PBS by heating. Once the temperature decreased to 42°C, thrombin solution (4 μl, 1000 U ml^−1^) and bovine fibrinogen solution (50 mg) were added to the mixture. Then, the mixed solution (10 ml per well) was added to Φ 100 mm dishes. The microrobots (10^7^ CFU) were preprocessed under AMF (375 kHz, 20 min) and seeded on the surface of fibrin plate. Then, the lytic area was measured after incubation (37°C, 12 hours). Based on the urokinase standard curve, fibrinolytic activity was computed by measuring the lytic area.

For the fibrinolytic ability assay, fibrinogen (1 mg ml^−1^, 3.6 ml), FITC-labeled fibrinogen (1 mg ml^−1^, 400 μl), and thrombin (250 U, 1 μl) were thoroughly mixed. Subsequently, the mixed solution (200 μl per well) was added to Φ 25 mm dishes and incubated at 4°C for 12 hours. The microrobots were added to the surface of the FITC-labeled fibrin clots. Images of the FITC-labeled fibrin clots were captured via a live cell imaging system at four time points (0, 6, 9, and 12 hours) following different treatments. The fibrinolytic ability with different treatments was evaluated by the analysis of fluorescence intensity of fibrin clots with ImageJ software.

### Animal models

Female BALB/c mice (6 weeks old) and male C57 mice (6 weeks old) were obtained from GemPharmatech Co., Ltd. (Nanjing, China) and housed under specific pathogen-free conditions at the Laboratory Animal Center of Tongji University. All procedures complied with the institutional ethical guidelines for animal research (approval nos. TJAA07323106 and TJAA07324107; Laboratory Animal Research Center, Tongji University). For the orthotopic 4T1 tumor model, a suspension of 10^6^ 4T1 cells in PBS was injected into the right back of each mouse.

### Tumor mechanics analysis

When 4T1 tumor volumes reached ~200 mm^3^, mice were randomized into six groups (Control, Swarm, Swarm^NK^, Swarm^NK/Memory^, Swarm^NK/Memory^ + WMF–1 Hz, and Swarm^NK/Memory^ + WMF–5 Hz). Probiotic microrobots (10^7^ CFU, 100 μl) were triggered by AMF (375 kHz, 330 Oe, 6 min) prior to intratumoral administration. Tumor-bearing mice were placed in the workspace of the RMF (20 mT, 2 Hz, 30 min) to induce the probiotic microrobot swarm formation. Subsequently, tumor-bearing mice were placed in the primates-scale magnetic control setup to control the locomotion of the probiotic microrobot swarm. The tumors were excised at day 1, 3, 5, and 7 after different treatments and washed with PBS. The compressive modulus of tumor tissues was determined using an electronic universal testing machine (UTM2502, Shenzhen Suns Technology Co., Ltd.). The compression tests were performed at a constant rate of 5 mm min^−1^ until reaching 50% strain.

### Antitumor effect of microrobots in vivo

Upon reaching tumor volumes of ~50 mm^3^, 4T1 or Panc02 tumor-bearing mice were randomly allocated to six experimental groups: Control, Swarm, Swarm^NK^, Swarm^NK/Memory^, Swarm^NK/Memory^ + WMF–1 Hz, and Swarm^NK/Memory^ + WMF–5 Hz. The 4T1 tumor-bearing mice were randomly divided into five groups (Control, Dox, Static microrobots, Swarm^NK/Memory^ + WMF–5 Hz, and Swarm^NK/Memory^ + WMF–5 Hz + Dox) when the tumor volume reached about 50 mm^3^. Mice in the Dox treatment group received intratumoral injections of Dox (3 mg kg^−1^ per mouse) on day 0, 3, and 6 (*82*). Static probiotic nanoparticle carriers were prepared by removing bacterial flagella using 0.5 M acetic acid (*55*, *56*), followed by MNP conjugation. The probiotic microrobots (10^7^ CFU, 100 μl) were preprocessed under AMF (375 kHz, 330 Oe, 6 min) and intratumorally injected into the mice. Tumor-bearing mice were placed in the workspace of the RMF (20 mT, 2 Hz, 30 min) to manipulate the probiotic microrobot swarm formation. Subsequently, tumor-bearing mice were placed in the primates-scale magnetic control setup to control the locomotion of probiotic microrobot swarm (74 mT, 5 Hz, 60 min) on day 1, 2, and 3. The following equation was used to calculate the tumor volume

$$V=\frac{\pi {\mathrm{LW}}^{2}}{6}$$ (3)

where *L* and *W* denote the longest and shortest tumor diameters, respectively. The survival duration of tumor-bearing mice was monitored daily until either natural death occurred or tumors exceeded 1500 mm^3^ in volume, at which point survival curves were terminated.

### Antitumor effect of microrobots combined with PD-1 inhibitor in vivo

The 4T1 tumor-bearing mice were randomly divided into six groups (Control, αPD-1, Swarm^NK/Memory^, Swarm^NK/Memory^ + αPD-1, Swarm^NK/Memory^ + WMF–5 Hz, and Swarm^NK/Memory^ + WMF–5 Hz + αPD-1) when the tumor volume reached about 50 mm^3^. The Robot^NK/Memory^ (10^7^ CFU, 100 μl) was preprocessed under an AMF (375 kHz, 330 Oe, 6 min) and subsequently injected intratumorally into the mice. Tumor-bearing mice were placed in the workspace of the RMF (20 mT, 2 Hz, 30 min) to induce the probiotic microrobot swarm formation. Subsequently, the mice were placed in the primates-scale magnetic control setup to control the locomotion of the probiotic microrobot swarm (74 mT, 5 Hz, 60 min) on day 1, 2, and 3. Mice in the αPD-1 treatment group received intraperitoneal injections of anti-mouse PD-1 antibody (100 μg per mouse) on day 0, 4, 8, and 12. Tumor volumes and body weights were measured every 48 hours, while survival status was assessed daily. The study endpoint was defined as either natural death or tumor volume exceeding 1500 mm^3^, upon which survival curves were terminated.

### In vivo imaging

The 4T1 or Panc02 tumor-bearing mice were randomly divided into three groups (Control, Swarm^NK/Memory^, and Swarm^NK/Memory^ + WMF–5 Hz) when the tumor volume reached about 200 mm^3^. The Robot^NK/Memory^ (10^7^ CFU, 100 μl) was preprocessed under an AMF (375 kHz, 330 Oe, 6 min) and subsequently injected intratumorally into the mice. Tumor-bearing mice were placed in the workspace of the RMF (20 mT, 2 Hz, 30 min) to induce the probiotic microrobot swarm formation. Subsequently, the mice were placed in the primates-scale magnetic control setup to control the locomotion of probiotic microrobot swarm (74 mT, 5 Hz, 60 min) on day 1, 2, and 3. To assess differences in tumor stiffness among treatment groups, ultrasound SWE (Mindray Nuewa R9 Pro, China; AIXPLORER Multiwave, France) and strain elastography (STE) imaging (Mindray Nuewa R9 Pro, China) were performed on day 0, 3, and 7. All ultrasound examinations were conducted by a specialized sonographer with over 3 years of clinical experience. MRI (uMR 9.4 T, United Imaging Life Science Instrument Co., Ltd., Wuhan, China) was used to monitor the microrobot swarm penetration within tumor tissues. In vivo fluorescence imaging was performed using an IVIS Spectrum imaging system (PerkinElmer), and the data were analyzed with Living Image software (PerkinElmer, USA).

### Flow cytometry analysis of tumor tissues

Tumor-infiltrating T cell populations were quantitatively assessed by flow cytometry on day 7 posttreatment. Tumors were dissected into small fragments and subsequently digested with a mixture of enzymes: hyaluronidase (0.025 mg ml^−1^), collagenase I (0.05 mg ml^−1^), deoxyribonuclease I (0.01 mg ml^−1^), collagenase IV (0.05 mg ml^−1^), and trypsin inhibitor (0.05 mg ml^−1^). These enzymes were sourced from Yuanye Bio-Technology Co., Ltd. (China). The tissue digestion protocol consisted of two sequential 15-min incubations at 37°C, with subsequent centrifugation (2300 rpm, 4 min). Following centrifugation, the supernatant was harvested and incubated with erythrocyte lysis buffer for 5 min at room temperature to remove contaminating red blood cells. This reaction was terminated using flow cytometry buffer (2.5% fetal bovine serum in PBS). Following collection, cells were stained with Fixable Viability Stain 510 (Live/Dead) and fluorescently labeled antibodies against CD45, CD3, CD4, and CD8 for phenotypic characterization. Samples were acquired on a Beckman CytoFLEX LX flow cytometer.

### Histological analysis

Following treatment and observation, murine major organs and tumor specimens were harvested and fixed in 4% paraformaldehyde. After paraffin embedding and sectioning, cellular iron deposition was assessed through PB staining. Collagen I and fibronectin immunofluorescent staining was used to characterize the ECM of tumors. The effect on cancer-associated fibroblasts was assessed by immunofluorescent staining with antibody specific for α-SMA.

### Biosafety assessment

The therapeutic safety profile was evaluated through comprehensive blood biochemistry and histopathological examination. Blood samples were collected in lithium heparin vacutainers, and plasma was obtained by centrifugation (3240 rpm, 4°C, 15 min). Biochemical parameters were quantified using a Cobas6000 autoanalyzer (Safety Evaluation Research Center, Shanghai Institute of Materia Medica, Chinese Academy of Sciences).

### Statistical analysis

Statistical analysis was performed using GraphPad Prism 8.4.2 and Origin Pro 9.1. All numerical data were expressed as mean ± SD in vitro and mean ± SEM in vivo of at least three replicates, unless otherwise stated. Statistical analysis between two groups was performed using an unpaired Student’s *t* test. For comparison of multiple groups, one-way analysis of variance (ANOVA) or two-way ANOVA was used, followed by Tukey’s honest significant difference post hoc test or Bonferroni’s multiple comparisons posttest. *P* < 0.05 was considered statistically significant, and all *P* values are indicated in the figures.
