# Supplementary material for: Magnetic medical microrobots with memory-capable genetic circuits
Source: Sci Adv. 2026 May 13;12(20):eaeb2528. doi: 10.1126/sciadv.aeb2528 (PMC13170677; doi:10.1126/sciadv.aeb2528)
Supplement: Supplementary file 1 — Text S1 to S3 Figs. S1 to S35 Tables S1 to S5 Legends for movies S1 to S6 [file sciadv.aeb2528_sm.pdf]

Science Advances
AAAS

Supplementary Materials for

Magnetic medical microrobots with memory-capable genetic circuits

Haotian Chen *et al.*

Corresponding author: Zhen Yin, zhenyin@tongji.edu.cn; Yu Cheng, yucheng@tongji.edu.cn

Sci. Adv. **12**, eaeb2528 (2026)
DOI: 10.1126/sciadv.aeb2528

The PDF file includes:

Text S1 to S3
Figs. S1 to S35
Tables S1 to S5
Legends for movies S1 to S6

Other Supplementary Material for this manuscript includes the following:

Movies S1 to S6

Supplementary Text

Text S1. *Molecular docking and molecular dynamics simulation*

Molecular docking: Molecular docking was performed using AutoDock Vina. The target protein was preprocessed using the predicted model, which included steps such as hydrogen removal, amino acid modification, energy optimization, and adjustment of force field parameters to achieve low-energy conformations for the ligand structure. Subsequently, molecular docking between the prepared target structure and the active compound was carried out using the Vina module integrated within Pyrx software. The affinity value (kcal mol^{-1}) obtained from the docking represents the binding capacity between the ligand and receptor, where lower values indicate more stable ligand-receptor binding. For visualization, the 3D structural analysis was conducted using PyMOL, while 2D interaction diagrams were generated using LigPlus.

Molecular dynamics simulation: To further explore the binding modes of the protein-small molecule complex, we conducted detailed molecular dynamics (MD) simulations using the Desmond program. During the simulation, the OPLS2005 force field was employed to parameterize the interactions between the protein and small molecules, while the TIP3P model was applied to simulate water molecules. The protein-small molecule complex was placed in a cubic water box, and sufficient sodium and chloride ions were added to neutralize the system charge. Before initiating the simulation, the system's energy was minimized using the steepest descent method for 50,000 steps to ensure a stable initial state. Two equilibration phases followed: the first was an NVT equilibration for 50,000 steps, and the second was an NPT equilibration for the same number of steps. During these phases, the positions of heavy atoms were restrained to help the system gradually adapt to the simulation environment. After the equilibration phases, a 100 ns unconstrained simulation was performed to observe the dynamic behavior of the protein-small molecule complex in its free state. Trajectory energy and coordinate data were recorded every 10 ps throughout the simulation. The system temperature was maintained at 300 K, and the pressure was kept at 1 bar to simulate physiological conditions.

Text S2. *Microrobot locomotion analysis*

To analyze the behavior of the tumbling magnetic microrobot, the model was confined to a cross-sectional view of the microrobot in the *y*-*z* plane, as shown in fig. S15A:

$$\begin{cases} m\ddot{y} = F_{my} + f_y - F_{\mathrm{d}} \\ m\ddot{z} = N - F_{mz} - G + L \\ I\ddot{\theta} = T + N\sin\theta - f_y\cos\theta - \tau_{\mathrm{d}} \end{cases} \quad (1)$$

where $F_{\mathrm{mag},y}$ and $F_{\mathrm{mag},z}$ denote the magnetic force along the y-direction and z-direction. f represents the frictional force obtained by the rotational dynamic equation. I denotes the moment of inertia, R is the contact radius, and θ is the angle between the chain and surface. F_{d} and τ_{d} present the force and torque of the dipole interactions. N is the normal force and L is the buoyancy force exerted on the microrobot. If the tumbling motion were ideal, the microrobots would move forward by approximately two body lengths. However, in the experiment shown in Fig. 4D and fig. S15, the microrobots moved much slower than the theoretical maximum velocity. This phenomenon indicates that tumbling motion coexisted with slipping. During the tumbling process, the frictional force is insufficient to support the microrobot's rolling for most of the time. Moreover, the conventional friction law, which states that frictional force is proportional to the normal force, does not apply here. Instead, the contact area plays a more significant role, and a larger contact area can significantly enhance the frictional force.

In the contact area, the surface profile can be approximated (via a second-order Taylor expansion) as:

$$y(x) \approx y(0) + \frac{1}{2} f''(0) \quad (2)$$

where the local effective radius of curvature is given by:

$$R = \frac{1}{f''(0)} \quad (3)$$

Since the curvature changes with the rotation angle, we model its variation with a cosine function. Let $R_{\max}$ be the effective curvature radius when the object's "fatter" (mid) section is in contact (i.e. the lowest local curvature), $R_{\min}$ be the effective curvature radius when the sharper tip is in contact (i.e. the highest curvature). A simple periodic model is:

$$R(t) = \frac{R_{\max} + R_{\min}}{2} + \frac{R_{\max} - R_{\min}}{2} \cos(\omega t) \quad (4)$$

where ω is the angular velocity and t is time. In Hertz theory for a sphere (or locally spherical surface) pressed against a flat surface, the contact is confined to a circular area. The radius a of this contact circle is:

$$a(t) = \left(\frac{3FR(t)}{4E^*}\right)^{1/3} \quad (5)$$

where F is the applied normal load, $E^*$is the effective (or composite) elastic modulus of the contacting materials given by $E^* = \frac{E}{1-v^2}$. The contact area in tumbling is:

$$A(t) = \pi a(t)^2 = \pi(\frac{3FR(t)}{4E^*})^{\frac{2}{3}} = \pi(\frac{3F}{4E^*})^{\frac{2}{3}} \left[\frac{R_{\max} + R_{\min}}{2} + \frac{R_{\max} - R_{\min}}{2} \cos(\omega t) \right]^{\frac{2}{3}} \quad (6)$$

Extra oscillating introduces extra periodic area, and the contact area in waving mode is:

$$A(t) = \pi(\frac{3FR(t)}{4E^*})^{\frac{2}{3}} = \pi(\frac{3F}{4E^*})^{\frac{2}{3}} R_{\text{avg}}^{\frac{2}{3}} \left[1 + A\cos(\omega t)\right]^{\frac{2}{3}} [1 + B\sin(\omega t)]^{\frac{2}{3}} \quad (7)$$

We take the parameters into the function, where a = 2×10^{-5} m, b = 2×10^{-6} m, Elastic coefficient E = 50 MPa, Poisson's ratio v = 0.4. In fig. S15B, compared with the tumbling mode, locomotion in waving mode has higher contact area.

For low load interface at microscale, the frictional force can be expressed as:

$$F_f(t) \approx \tau_f A(t) \quad (8)$$

During the tumbling process in our swarm, the interfacial shear strength τ_f does not vary significantly and can be considered constant. Therefore, the frictional force primarily depends on the contact area. In other words, the slipping behavior indicates that the contact area is insufficient to allow the microrobot to roll.

For any convex body, one may define its support function $h(n)$ as the distance (from the centroid) to the supporting line that is perpendicular to the unit vector n. Because the ellipse is rotated by an angle with respect to the world coordinates, the outward (upward) normal of the ellipse at the contact is obtained by "rotating" the vertical. In the body frame of the ellipse the supporting line in the direction has a distance (the support function) given by:

$$h(\varphi) = \sqrt{a^2 \sin^2 \varphi + b^2 \cos^2 \varphi} \quad (9)$$

The parameters a and b are the semi-axes; typically one chooses the vertical semi-axis smaller than a if the ellipse is "flattened" in the vertical direction. Thus, the horizontal displacement of the center during the small rotation is given by the x-component of:

$$dx_C = r_y d\varphi = \sqrt{a^2 \sin^2 \varphi + b^2 \cos^2 \varphi} d\varphi \quad (10)$$

Thus, the instantaneous horizontal (x-direction) velocity of the centroid is:

$$v_x = \frac{dx_C}{dt} = \frac{d\varphi}{dt} \sqrt{a^2 \sin^2 \varphi + b^2 \cos^2 \varphi} \quad (11)$$

If we denote $\omega = \frac{d\varphi}{dt}$, then

$$v_x(t) = \omega\sqrt{a^2 \sin^2 \varphi(t) + b^2 \cos^2 \varphi(t)} \tag{12}$$

We know that $a > b$, thus $v_x(t)$ gets maximum value:

$$v_{x,\max} = \omega a, \text{attained when } \omega t = \pi/2,\ 3\pi/2, ... \tag{13}$$

$v_x(t)$ gets minimum:

$$v_{x,\min} = \omega b, \text{attained when } \omega t = 0, \pi,\ 2\pi, ... \tag{14}$$

The results in fig. S15C indicate that if the slipping occurs, the highest velocity period will be missing, which would lead to a huge loss of locomotion capabilities. We can obtain that the average velocity in the tumbling mode is 0.285 L s^{-1}, while the velocity in the waving mode is 0.603 L s^{-1}, which is in concert with our experimental result in Fig.4D.

Text S3. Theoretical prediction of microrobot locomotion velocity as a function of matrix stiffness

At low speeds, the robot's velocity is well-approximated by a static balance

$$v \approx \frac{F}{D(E,c)}, \qquad D(E,c) = A\alpha E\frac{m}{a} + \frac{A\mu}{k(c)} + C(c). \tag{15}$$

Here F is the effective driving force produced by the waving actuation. The denominator $D(E,c)$ is the total resistance. Its first term, $A\ \alpha E\ \frac{m}{a}$, represents a yield/friction contribution that scales with the local effective stiffness E of the matrix; A is the frontal area set by the robot radius a, α is an effective yield strain, and m/a sets the regularization scale of the yield law. The second term, $A\mu/k(c)$, is the poro-viscous resistance to interstitial flow through the porous extracellular matrix. The third term, $C(c)$, captures additional fibrous/viscous drag from the matrix network.

Secretion elevates the local softening level c in front of the robot, which reduces the effective stiffness E (and, in practice, also increases k and decreases C). Consequently the total resistance $D(E,c)$ becomes smaller and the speed $v = F/D$ becomes larger. Because forward motion transports the secreted softener toward the front, the softening level ahead (c_{front}) increases further, which lowers D even more and again raises the speed. In words,

$$v\uparrow \Rightarrow c_{\text{front}}\uparrow \Rightarrow D\downarrow \Rightarrow v\uparrow \tag{16}$$

so penetration promotes softening, and softening in turn facilitates faster penetration.

This mechanism is consistent with our measurements: when the effective stiffness is reduced from 20 to 5 kPa by secretion (a four-fold softening), the yield-dominated resistance in D falls by roughly the same factor, producing an observed $\sim 4\times$ increase in steady velocity and penetrated distance (fig S23, F and G).

Supplementary Figures

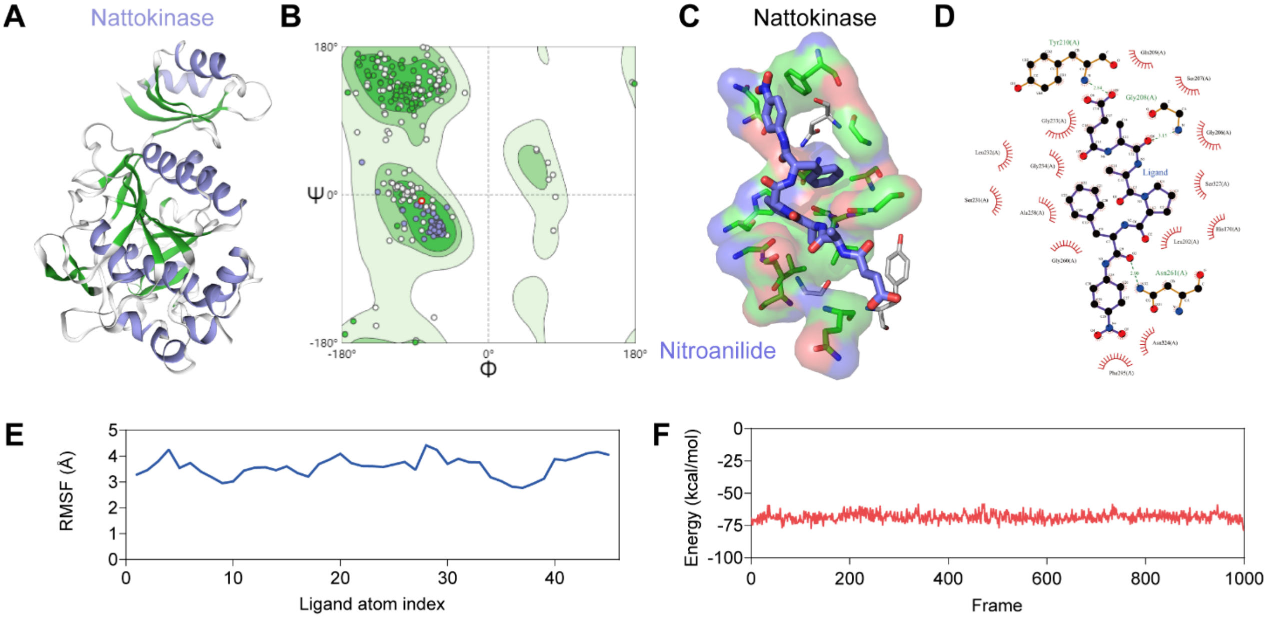

Fig. S1. Simulation structure of NK and molecular dynamics simulations. (**A**) Three-dimensional simulation structure of NK. (**B**) Evaluation of the simulation structure of NK based on Ramachandran plots. (**C**) Molecular dynamics simulation results for the binding of tetrapeptide substrate (N-Succinyl-Ala-Ala-Pro-Phe-p-Nitroanilide) with NK. (**D**) Top view of the 17 active-site residues. The red and green indicate hydrophobic interactions and hydrogen bonds, respectively. (**E**) Root mean square fluctuation (RMSF) analysis diagram of tetrapeptide substrate during a 20-100 ns molecular dynamics simulation. (**F**) Molecular mechanics generalized born surface area (MM/GBSA) analysis of tetrapeptide substrate and NK complexes.

The online simulation server SWISS-MODEL (https://swissmodel.expasy.org/) was used to construct the simulated crystal structure of NK (fig. S1A). The Ramachandran diagram showed 94.2% of the Φ and Ψ angles of the residues in the NK model fell in the optimal conformational region, and only 0.29% were in the outliers (fig. S1B). The global model quality estimation was 0.87 and the QMEANDisCo global value was 0.86 ± 0.05. Model evaluation indicated that the model was a satisfactory match with the template. The tetrapeptide substrate formed hydrophobic interactions with 17 active-site residues near the active pocket of NK, including Tyr^{210}, Gly^{208}, Asn^{261}, Gln^{209}, Ser^{207}, Gly^{206}, Ser^{327}, His^{170}, Leu^{202}, Asn^{324}, Phe^{295}, Gly^{260}, Ala^{258}, Ser^{231}, Leu^{232}, Gly^{233}, Gly^{234} (fig. S1, C and D). This hydrophobic interaction usually enhances the affinity between ligands and proteins, thus helping to stabilize the binding between them. Simultaneously,

three hydrogen bonds were formed between tetrapeptide substrate and active-site residues of NK, including Tyr^{210}, Gly^{208}, and Asn^{261}. Hydrogen bonds are usually one of the main sources of binding force in the interaction between protein and ligand.

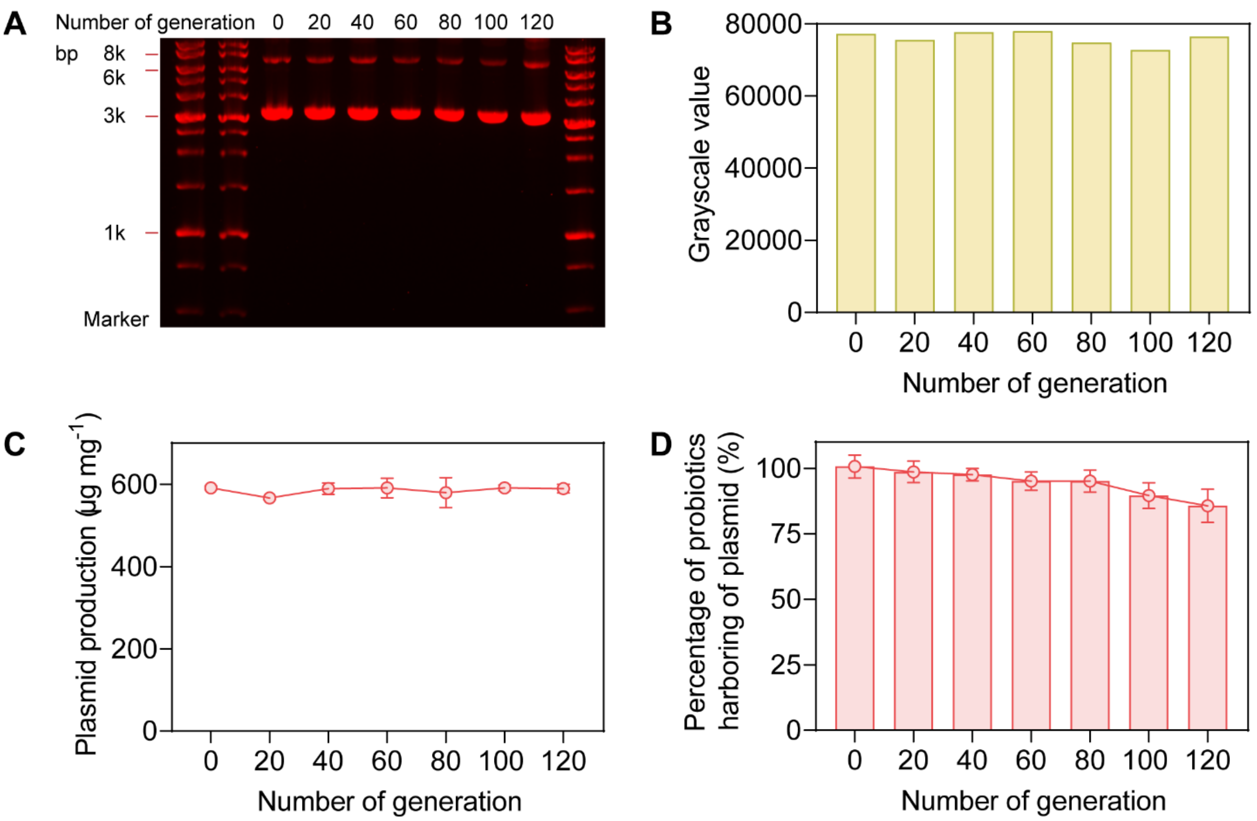

Fig. S2. Long-term stability and fidelity of genetic memory. (**A**) Plasmids extracted from engineered *E. coli* Nissle 1917 at different generations (0, 20, 40, 60, 80, 100 and 120) were analyzed by agarose gel electrophoresis. (**B**) Grayscale value of the plasmid bands at different generations. (**C**) The plasmid productions at different generations with the same bacterial weight (n = 3; means ± SD). (**D**) The percentage of probiotics harboring the plasmid at different generations (0, 20, 40, 60, 80, 100 and 120). Plasmid retention rate = (number of colonies on antibiotic-containing medium / number of colonies on antibiotic-free medium) × 100%; (n = 3; means ± SD).

To assess the long-term stability and fidelity of the genetic memory, we performed stability tests on engineered *E. coli* Nissle 1917 over 120 consecutive generations. Plasmids extracted from different generations were compared with the original, and agarose gel electrophoresis showed

identical band patterns and intensities, confirming structural stability (fig. S2, A and B). The plasmid yield was approximately 600 μg per gram of wet bacterial mass, indicating that the plasmid quantity remained essentially constant during passaging (fig. S2, C). To evaluate plasmid retention, engineered strains from every 20th generation were plated on LB agar with or without ampicillin. The retention rate remained above 95.14 ± 4.22% within the first 80 generations and slightly decreased thereafter, yet approximately 85.48 ± 8.08% of cells still retained the genetic memory plasmid after 120 generations, demonstrating durable inheritance and stability (fig. S2, D).

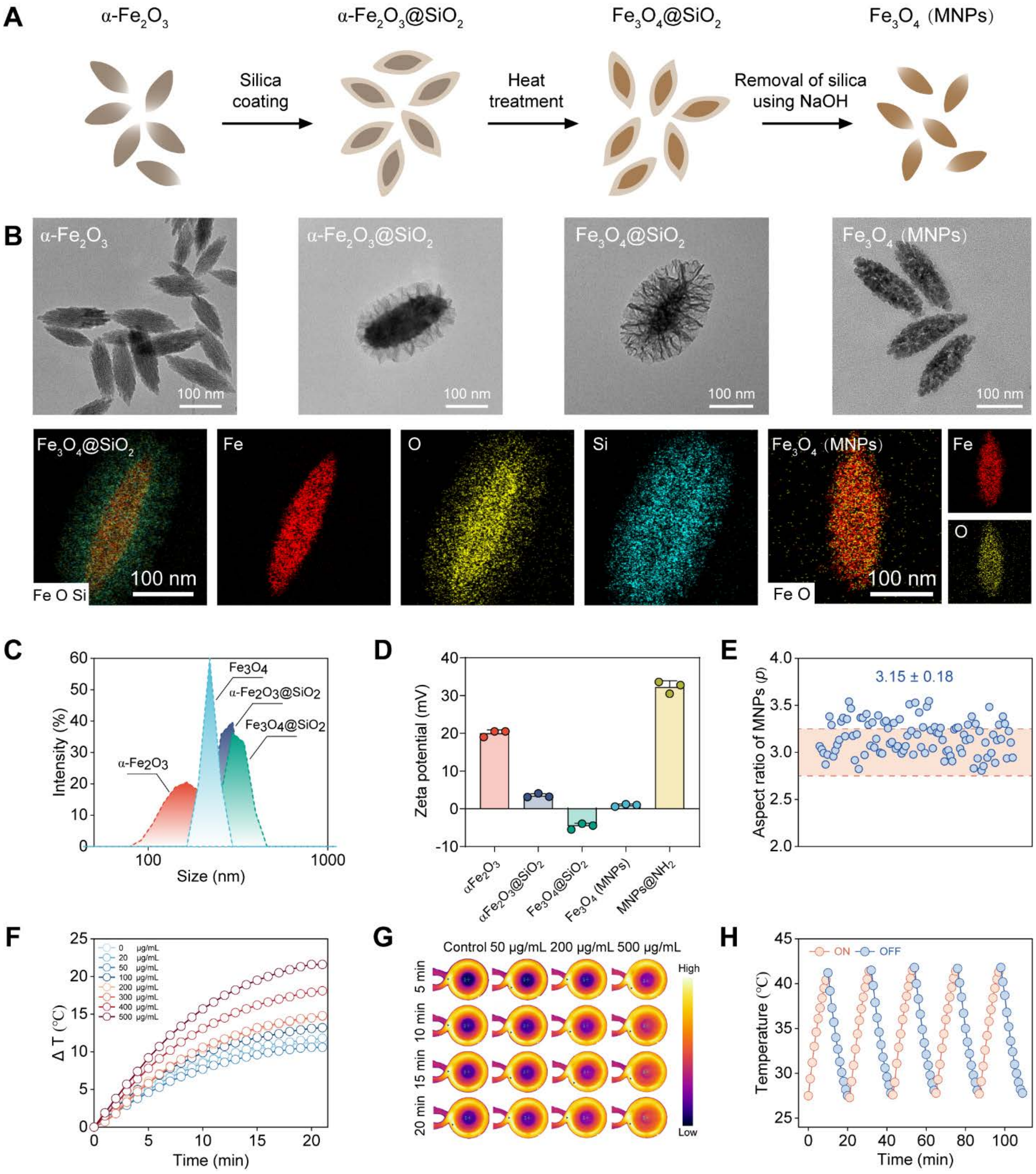

Fig. S3. Synthesis and characterization of magnetic nanoparticles (MNPs). (**A**) Schematic illustration of the procedure for the synthesis of uniform MNPs. (**B**) TEM images of α-Fe_2O_3, α-Fe_2O_3@SiO_2, Fe_3O_4@SiO_2 and Fe_3O_4 (MNPs); and elemental mapping images of Fe_3O_4@SiO_2 and Fe_3O_4 (MNPs). (**C**) The hydrodynamic size of α-Fe_2O_3, α-Fe_2O_3@SiO_2, Fe_3O_4@SiO_2 and Fe_3O_4 (MNPs). (**D**) Zeta potential of α-Fe_2O_3, α-Fe_2O_3@SiO_2, Fe_3O_4@SiO_2, Fe_3O_4 (MNPs) and

MNPs@NH_2 (n = 3; means ± SD). (**E**) Analysis of the average aspect ratio of MNPs (n = 100; means ± SD). (**F**) Temperature elevation of MNPs at different concentrations. (**G**) Real-time thermal images of the MNPs (375 kHz, 330 Oe, 20 min). (**H**) Temperature changes of MNPs during five cycles of AMF treatment (ON; 375 kHz, 330 Oe) and natural cooling-down (OFF).

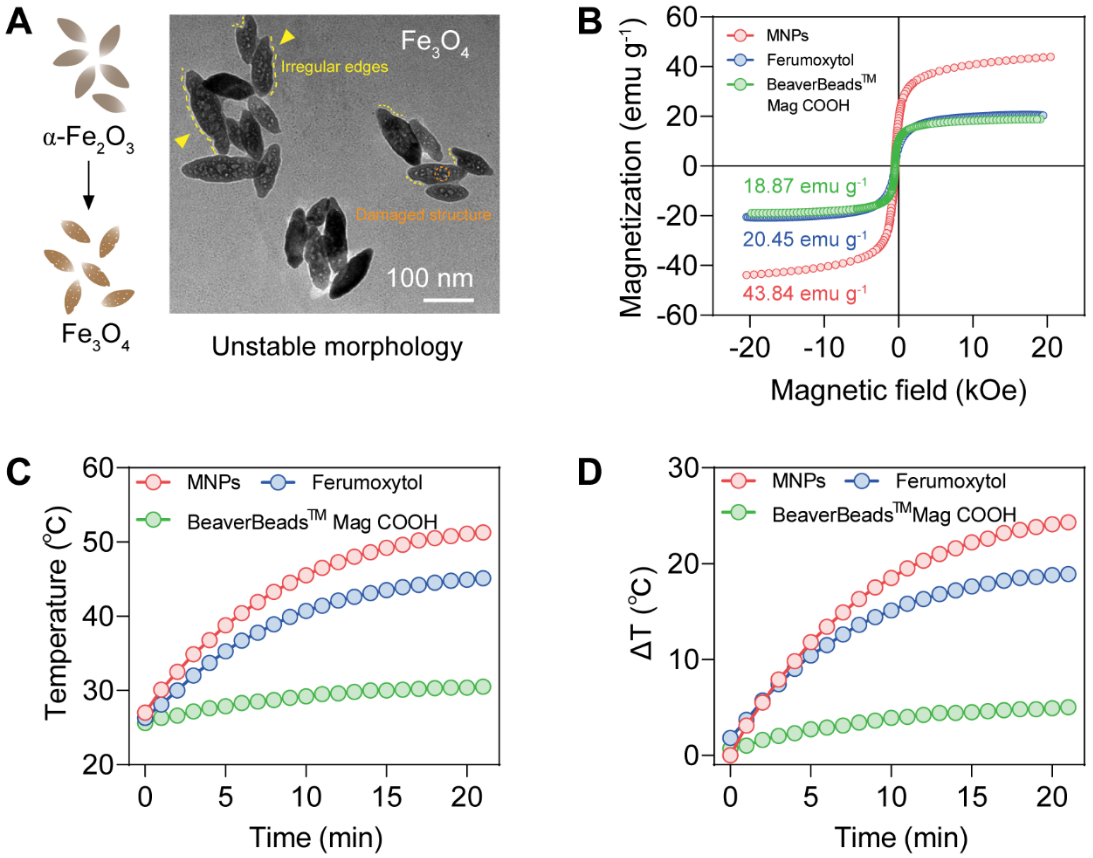

Fig. S4. Comparison of saturation magnetization and magnetothermal effect of MNPs, Ferumoxytol (FDA-approved clinical ferrite nanomaterial), and BeaverBeads^{TM} Mag COOH (commercial magnetic beads with carboxyl groups). (**A**) TEM image of α-Fe_2O_3 directly reduced to Fe_3O_4. (**B**) Magnetic hysteresis curves of MNPs, Ferumoxytol (FDA-approved clinical ferrite nanomaterial), and BeaverBeads^{TM} Mag COOH measured at ± 20 kOe. (**C**) and (**D**) Temperature profiles of MNPs, Ferumoxytol, and BeaverBeads^{TM} Mag COOH over time under AMF stimulation (375 kHz, 330 Oe).

The TEM images revealed irregular edges and structural damage, indicating that direct reduction caused morphological instability (fig. S4A). To obtain Fe_3O_4 nanoparticles with stable morphology,

a silica coating was introduced as a protective layer. To further validate the quality of the synthesized MNPs, we compared them with FDA-approved clinical ferrite nanomaterial Ferumoxytol and commercial magnetic beads with carboxyl groups (BeaverBeads™ Mag COOH, 1 μm). The synthesized MNPs exhibited a saturation magnetization of 43.84 emu g^{-1}, which was more than twice that of Ferumoxytol (20.45 emu g^{-1}) and BeaverBeads™ Mag COOH (18.87 emu g^{-1}) (fig. S4B). The temperature of the synthesized MNPs steadily increased by 24.3°C under alternating magnetic field (AMF) stimulation (375 kHz, 330 Oe, 20 min), significantly higher than Ferumoxytol (18.9°C) and BeaverBeads™ Mag COOH (5.0°C) (fig. S4, C and D).

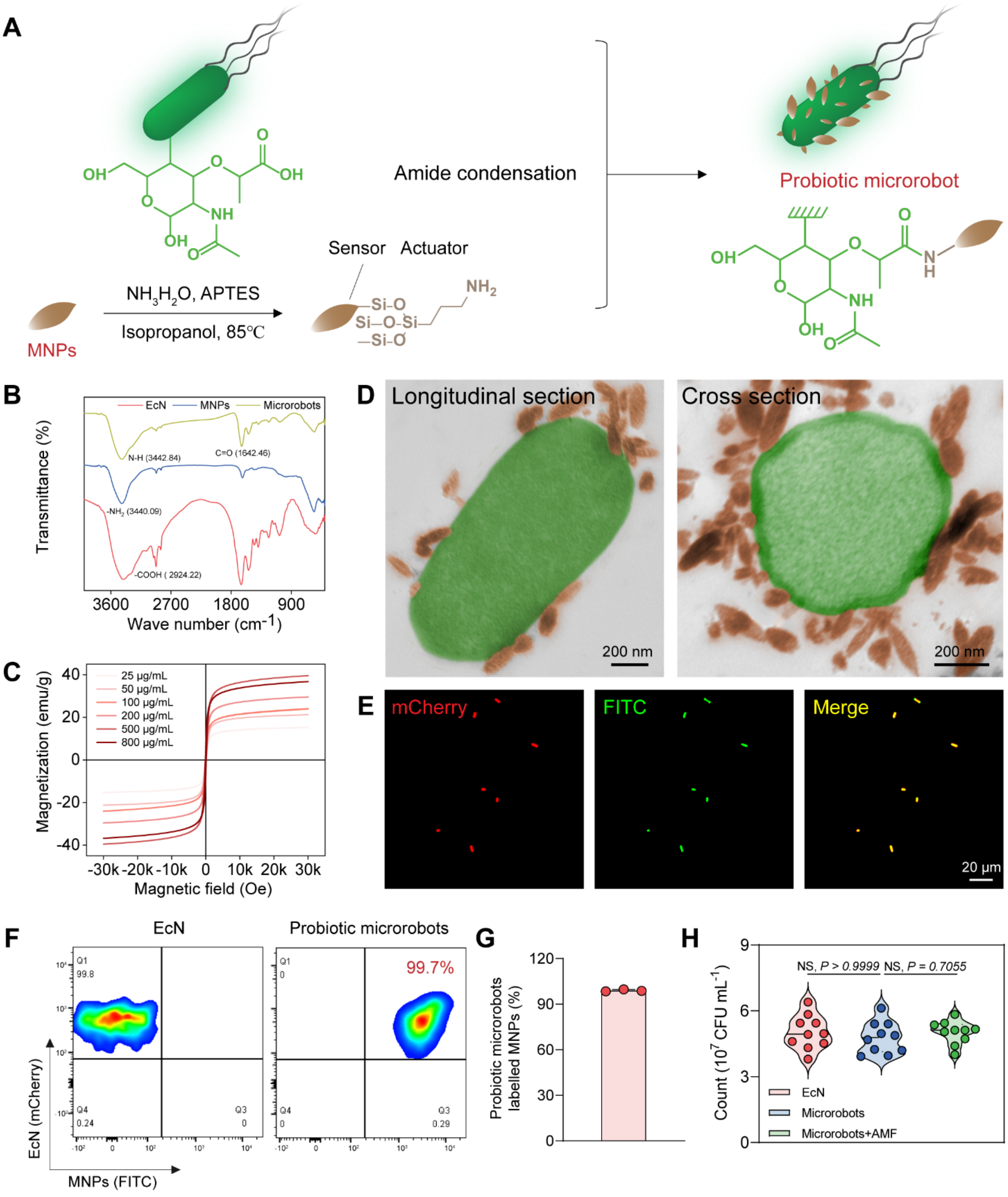

Fig. S5. Characterization of probiotic microrobots. (**A**) Schematic modification of probiotics with magnetic nanoparticles through amide condensation. (**B**) Fourier transform infrared spectroscopy (FTIR) of EcN, MNPs, and Microrobots. (**C**) Magnetic hysteresis curves of probiotic microrobots modified with different concentrations of MNPs. (**D**) Bio-TEM images of probiotic

microrobots. Images were pseudo-colored (Green, brown colors represent probiotics and magnetic control units, respectively). (**E**) LSCM images of MNPs@FITC conjugated microrobots. The mCherry and FITC indicate probiotics and MNPs@FITC, respectively. (**F**) and (**G**) Flow cytometry analysis of probiotic microrobots before (left) and after (right) conjugation with FITC-labelled MNPs (n = 3; means ± SD). (**H**) Viabilities of probiotic microrobots after conjugation with MNPs and under AMF (375 kHz, 330 Oe, 20 min) (n = 10; means ± SD).

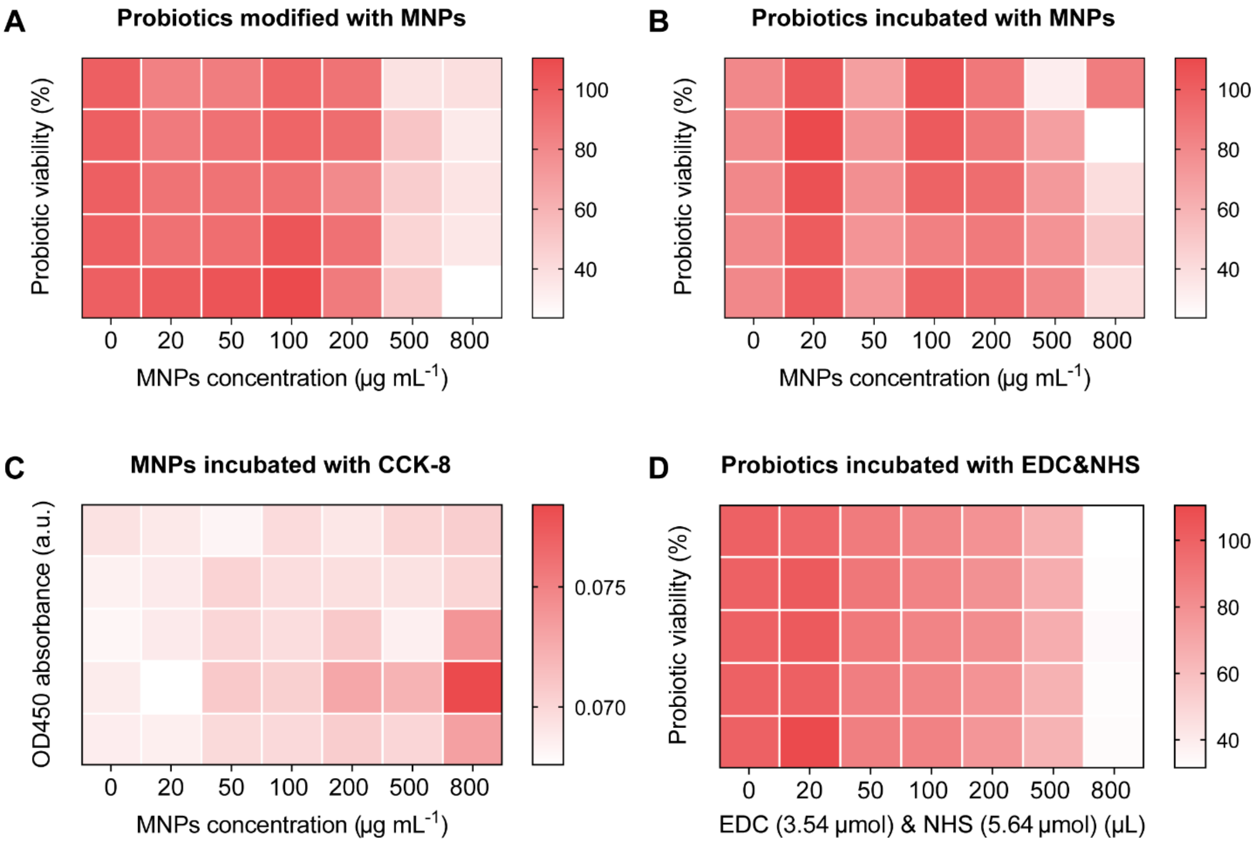

Fig. S6. Viability of probiotics after modification with different concentrations of MNPs. (**A**) Probiotic viability assessed by CCK-8 after modification with different concentrations of MNPs (n = 5). (**B**) Probiotic viability assessed by CCK-8 after incubation with different concentrations of MNPs (n = 5). (**C**) The absorbance at OD450 was tested after CCK-8 incubation with different concentrations of MNPs (n = 5). (**D**) Probiotic viability assessed by CCK-8 after incubation with different concentrations of EDC and NHS (n = 5).

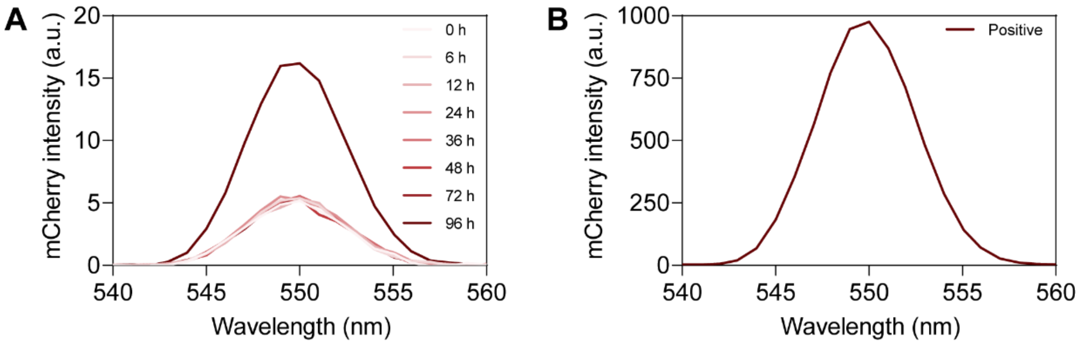

Fig. S7. The mCherry fluorescence intensity of microrobots under prolonged exposure at 39.5°C. (**A**) The mCherry fluorescence intensity of microrobots at different time points (0, 6, 12, 24, 36, 48, 72 and 96 h) were detected after exposure to 39.5°C. (**B**) The mCherry fluorescence intensity of microrobots was detected after exposure to 42°C.

To evaluate the stability of the untriggered state, we tested the response of the microrobots at a prolonged, fever-range temperature of 39.5°C. No activation of the memory circuit was observed during a 72-hour period (fig. S7A). Compared with the positive control group, approximately 1.11% mCherry fluorescence expression was observed after 96 h, a level comparable to background expression in the negative control group (fig. S7, A and B). Because temperature elevations associated with febrile illness or localized inflammation are typically transient and rarely exceed 40 °C for such durations, these results indicate that the genetic circuit remains stably untriggered under physiologically relevant stress. Collectively, the data confirm that the memory system is resistant to inadvertent activation within normal fever-range conditions.

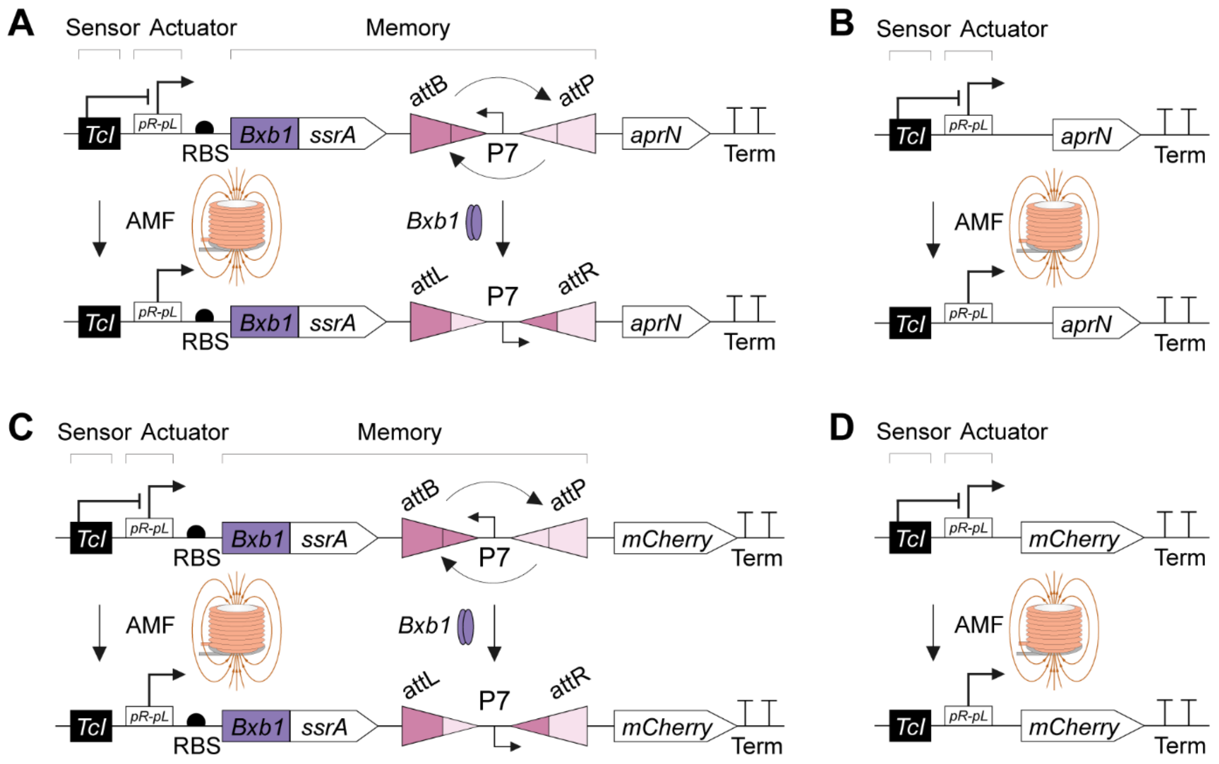

Fig. S8. Genetic circuit illustration of probiotic microrobots. (**A**) Illustration of the genetic circuit of probiotic microrobots with memory device (signal output modules: NK). (**B**) Illustration of the genetic circuit of probiotic microrobots without memory device (signal output modules: NK). (**C**) Illustration of the genetic circuit of probiotic microrobots with memory device (signal output modules: mCherry). (**D**) Illustration of the genetic circuit of probiotic microrobots without memory device (signal output modules: mCherry).

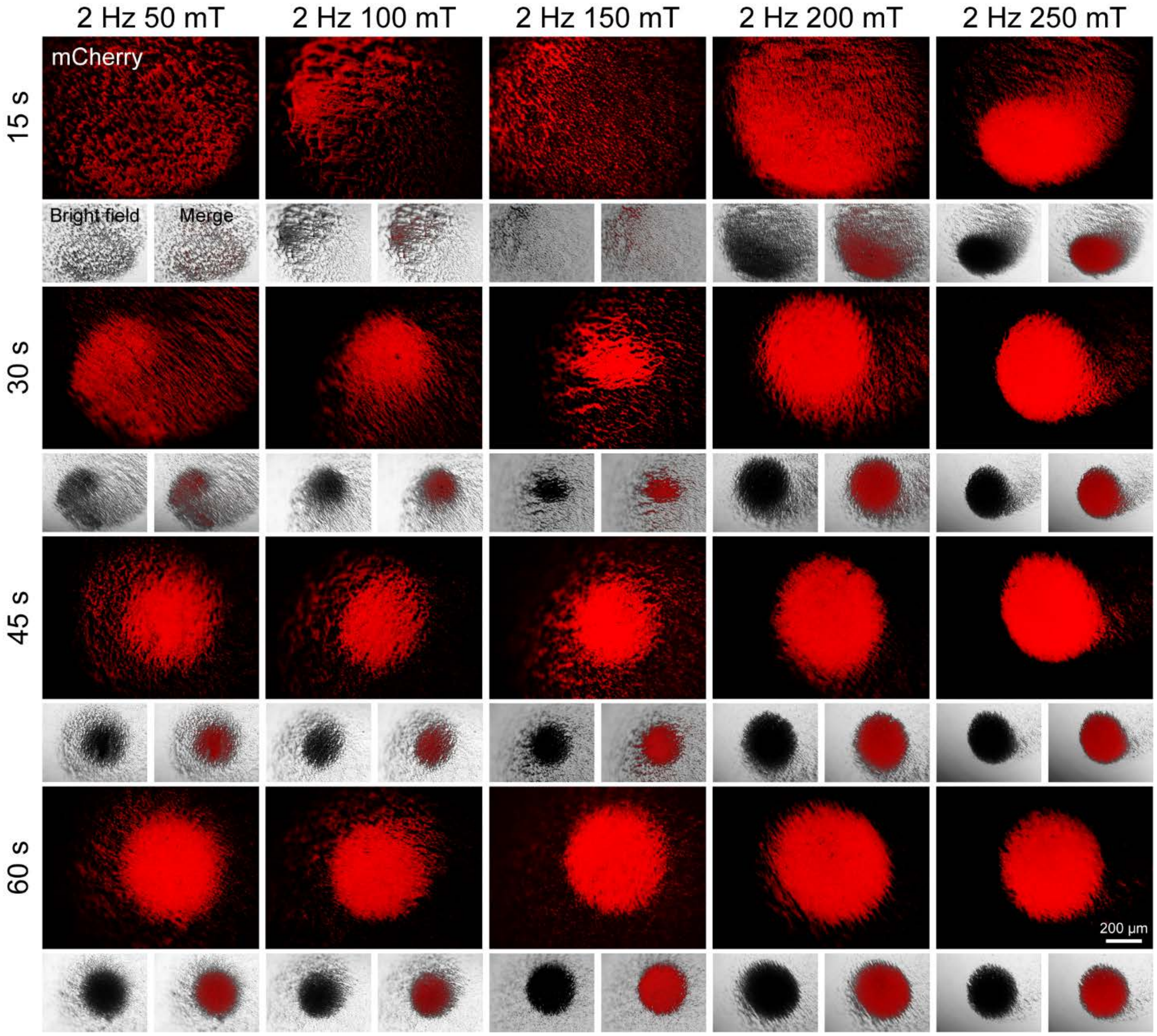

Fig. S9. Probiotic microrobot swarm formation with different magnetic field intensities (50 mT, 100 mT, 150 mT, 200 mT, and 250 mT) and time (15 s, 30 s, 45 s, and 60 s). mCherry indicates probiotic microrobots.

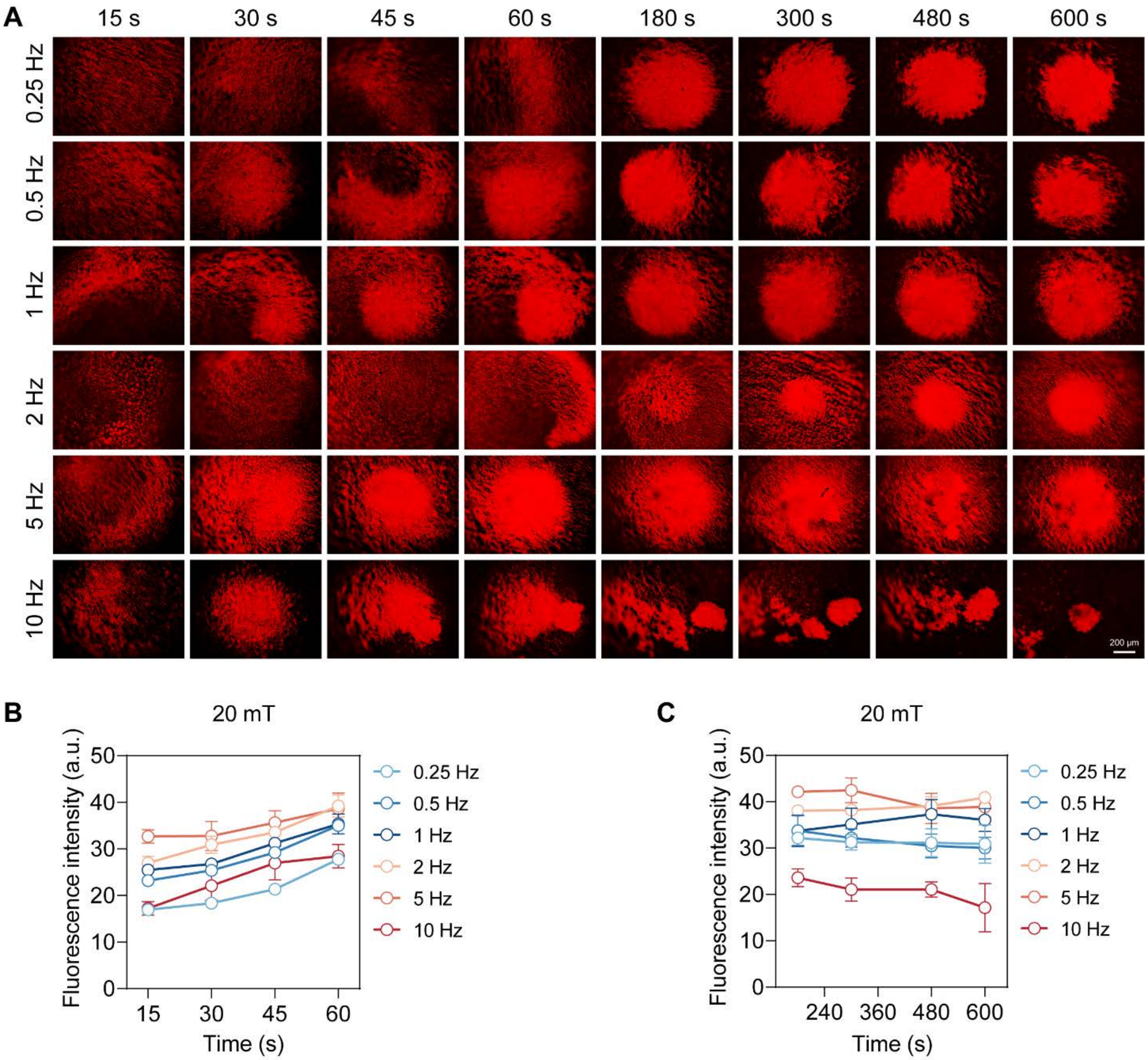

Fig. S10. Probiotic microrobot swarm formation with different magnetic field frequencies and time. (**A**) Representative fluorescence imaging of the probiotic microrobot swarm with different input frequencies and time (20 mT). (**B**) and (**C**) Quantitative fluorescence intensity of the probiotic microrobot swarm varies with input frequencies and time (n = 3; means ± SD).

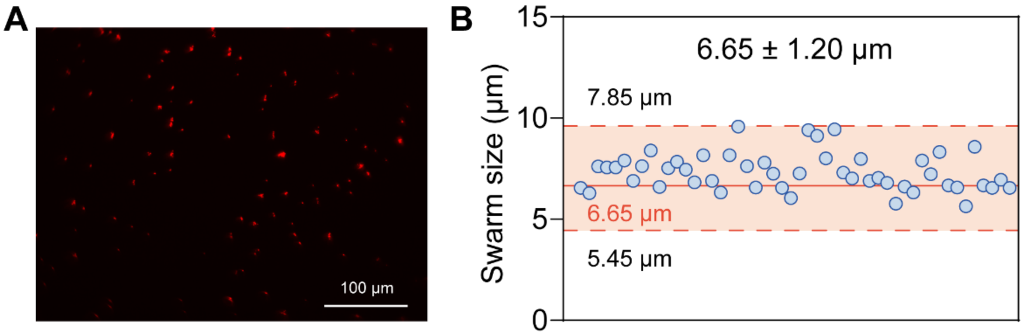

Fig. S11. The size of probiotic microrobot swarm under RMF (20 mT, 2 Hz, 10 min). (**A**) Representative fluorescence imaging of the microrobot swarm with the input doses of 10^3 CFU under RMF (20 mT, 2 Hz, 10 min). (**B**) Probiotic microrobot swarm size with the input doses of 10^3 CFU under the same RMF (20 mT, 2 Hz, 10 min) (n = 50).

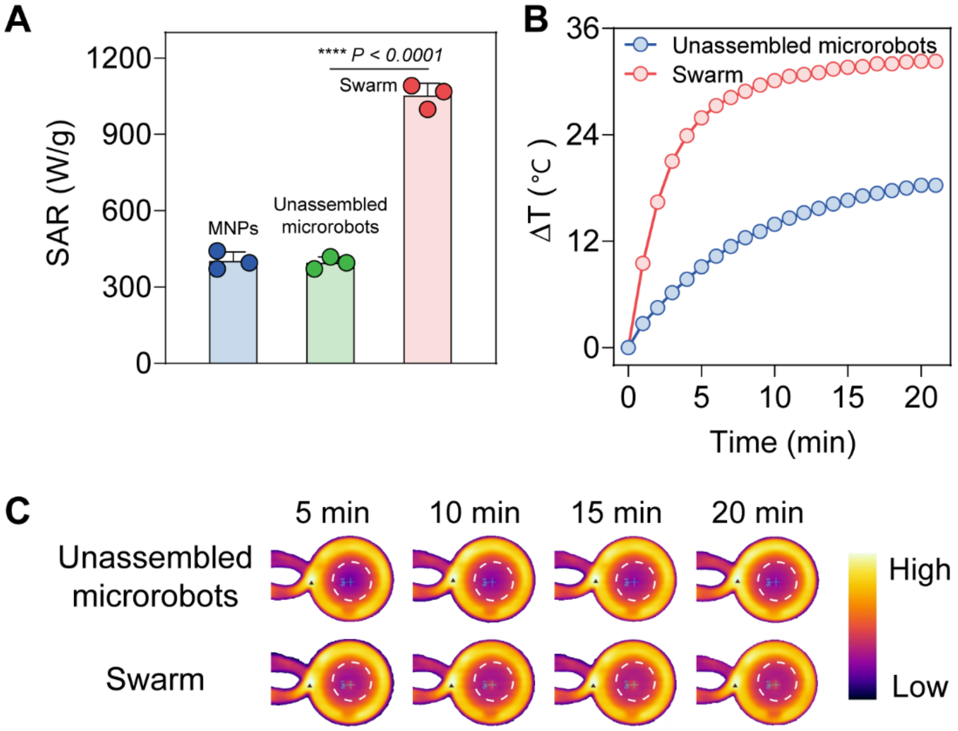

Fig. S12. The thermal effects of probiotic microrobot swarm. (**A**) Specific absorption rate (SAR) of unassembled microrobots and densely aggregated swarm under AMF (n = 3; means ± SD). (**B**) Temperature profiles of unassembled microrobots and densely aggregated swarm under AMF. (**C**) Real-time thermal images of the unassembled microrobots and densely aggregated swarm.

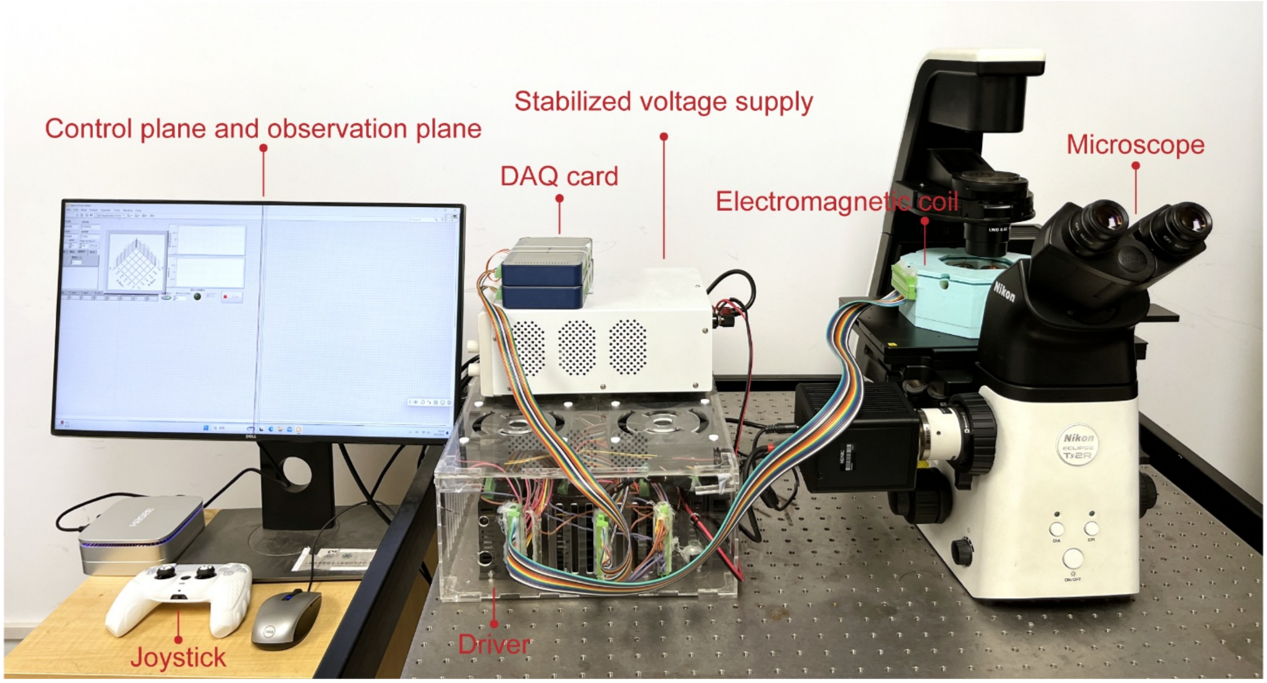

Fig. S13. Magnetic torque actuation and visualization system. A lab PC works as the host computer for the high-level human-computer interface and visualization of data. A joystick is invoked as input equipment for manual trajectory control. LabVIEW (National Instruments, Inc.) is employed to realize the preparation of relevant algorithms and control programs. Driving signals are generated by a high-speed DAQ (data acquisition) card and then amplified by the voltage amplifier before entering the coils. An optical microscope (Nikon, ECLIPSE Ts2R) is utilized to observe the real-time position and state of the microrobots and provide feedback to the controllers.

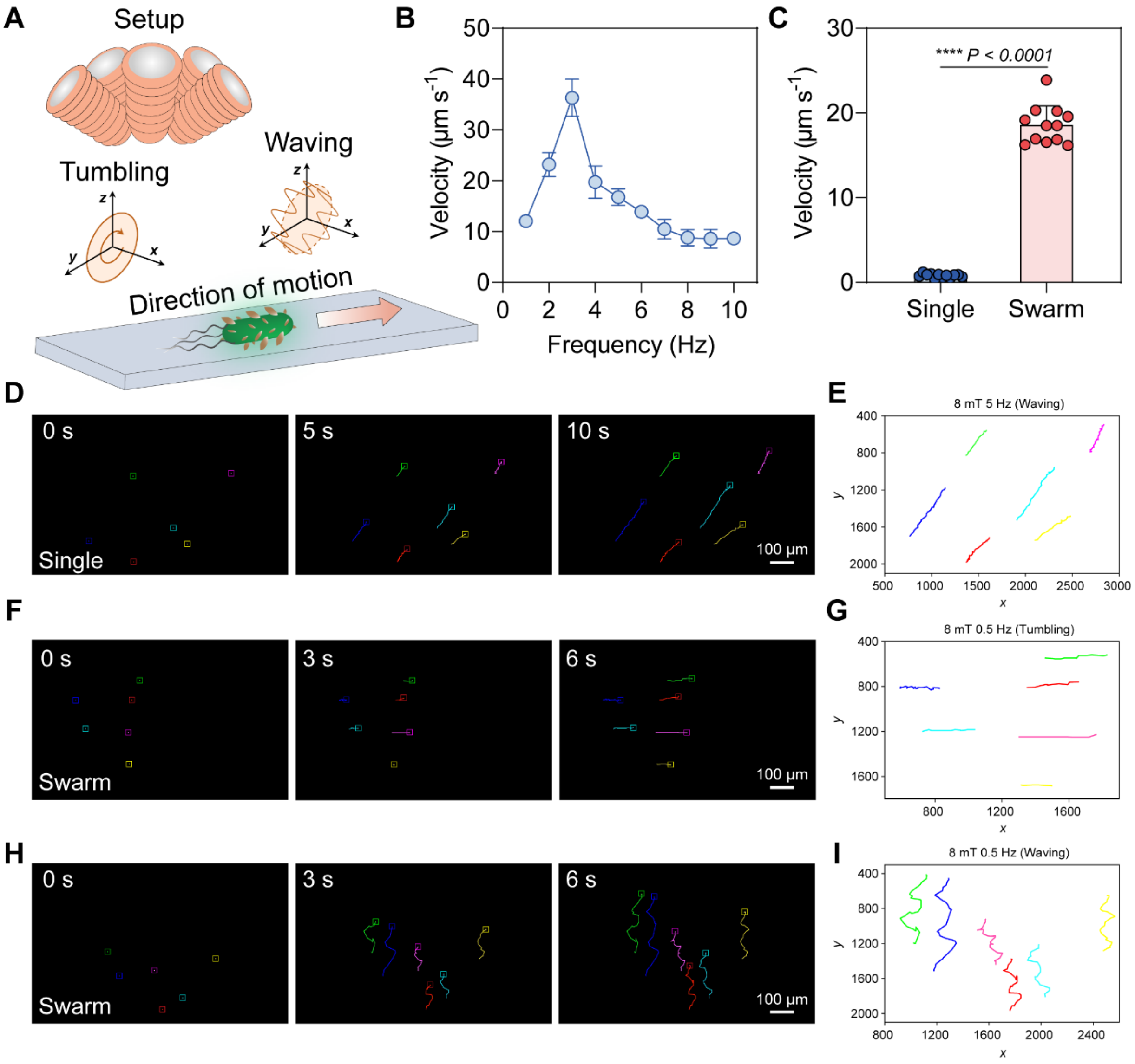

Fig. S14. Actuation of individual microrobot and microrobot swarm. (**A**) Schematic design of probiotic microrobot tumbling and waving motion under the magnetic field. (**B**) Velocity of the individual probiotic microrobot versus the frequency of the waving magnetic field (8 mT, Waving) (n = 10; means ± SD). (**C**) The velocities of the individual microrobot and microrobot swarm under the application of waving magnetic field (8 mT, 1 Hz) (n = 12; means ± SD). (**D**) and (**E**) Time-lapse image and trajectories illustrating the controllable magnetic propulsion of individual microrobot (8 mT, 5 Hz). (**F**) and (**G**) Time-lapse image and tumbling trajectories illustrating controllable magnetic propulsion of microrobot swarm (8 mT, 0.5 Hz). (**H**) and (**I**) Time-lapse image and waving trajectories illustrating controllable magnetic propulsion of microrobot swarm (8 mT, 0.5 Hz).

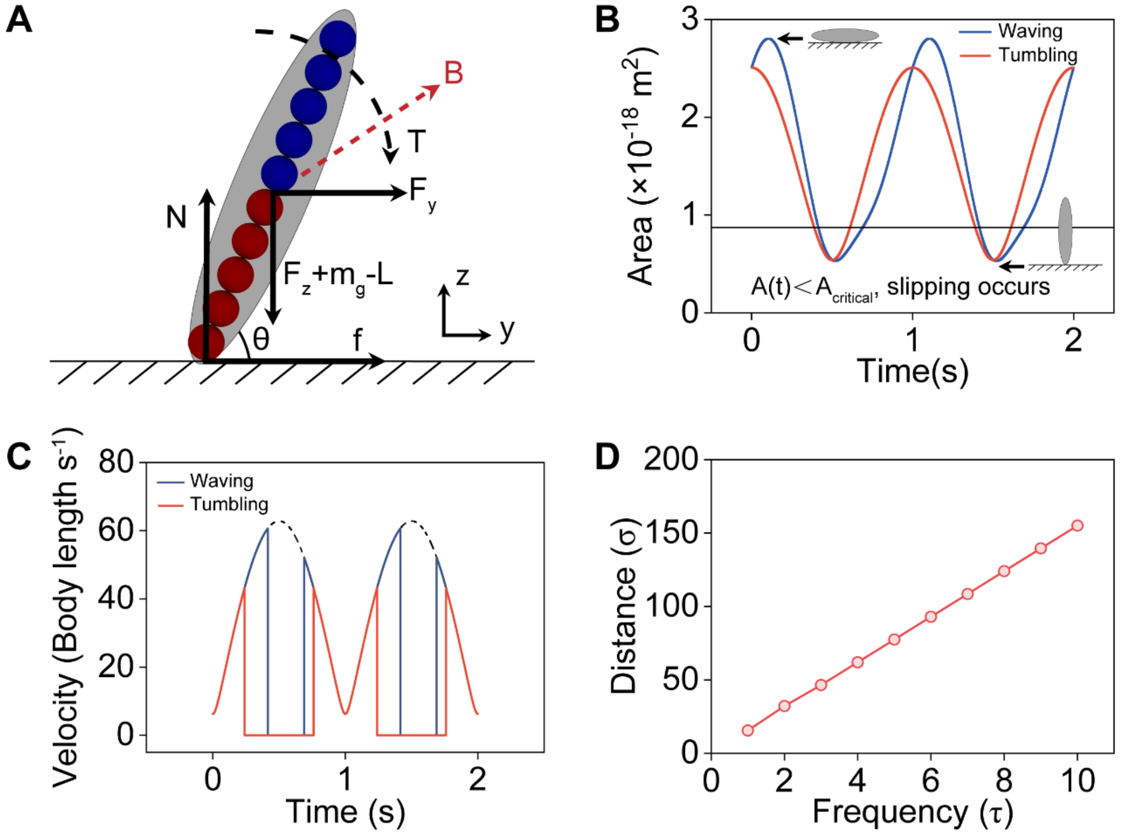

Fig. S15. Theoretical analysis of microrobot swarm locomotion. (**A**) Schematic illustration of a particle chain undergoing tumbling motion under an applied magnetic field. (**B**) Comparison of the contact areas between the surface and the microrobot swarm in waving mode and tumbling modes, respectively. (**C**) Mixed slipping-rolling velocities in waving and tumbling modes. (**D**) Comparison of swarm motion distances at various magnetic field frequencies.

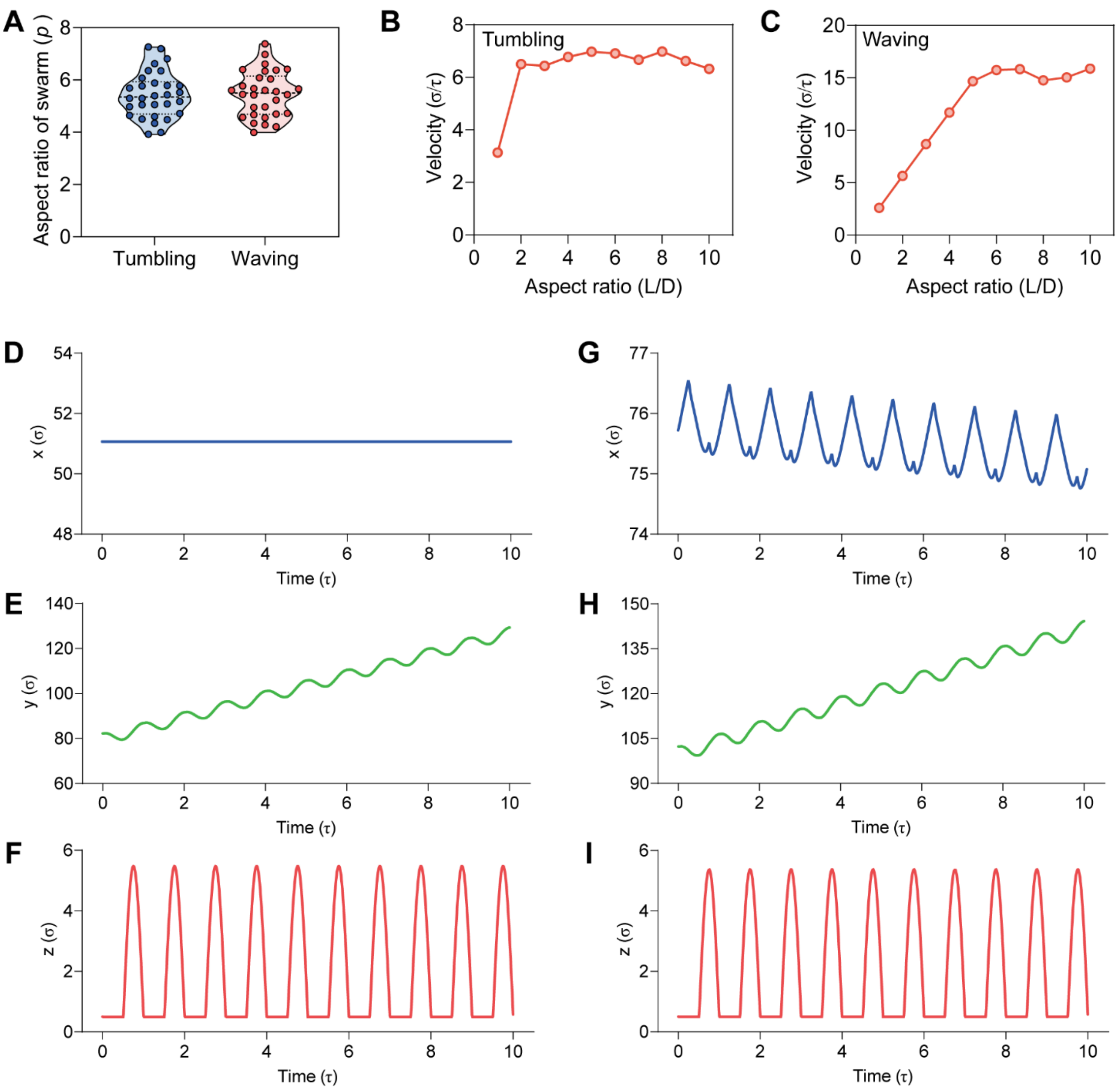

Fig. S16. Microrobot swarm motion under tumbling and waving modes. (**A**) The distribution of aspect ratios within a swarm after assembly ($n = 30$; means ± SD). (**B**) The relationship between aspect ratio and velocity in the tumbling mode. (**C**) The relationship between aspect ratio and velocity in waving mode. (**D**) to (**F**) The coordinate variations of an individual microrobot with an aspect ratio of 5 in tumbling mode along the *x*, *y*, and *z* directions respectively. (**G**) to (**I**) The coordinate variations of an individual microrobot with an aspect ratio of 5 in waving mode along the *x*, *y*, and *z* directions respectively.

To compare the influence of shape on microrobot locomotion, we constructed micro-chains with aspect ratios ranging from 1 to 10 and applied a rotating magnetic field with a frequency of 1 τ^{-1}. The rotating field simulated the tumbling mode, while an additional oscillating field perpendicular to the main rotation plane was introduced to simulate the waving mode. As the aspect ratio increased, the velocity of microrobots initially increased due to the enlarged rotational radius (fig. S16B). However, a larger radius was not always advantageous. Under the combined effects of gravity, flow fields, and friction, longer chains tended to fold spontaneously into configurations with lower effective aspect ratios, achieving higher velocities (fig. S16C). For instance, a chain with an initial aspect ratio of 10 would spontaneously fold to an aspect ratio of approximately 7 after only three rotational cycles. Additionally, within a swarm, smaller chains and isolated particles were frequently attracted to and integrated into neighboring larger chains, which aligns well with experimental observations showing that the aspect ratios within a swarm typically varied between 4 and 8 (fig. S16A).

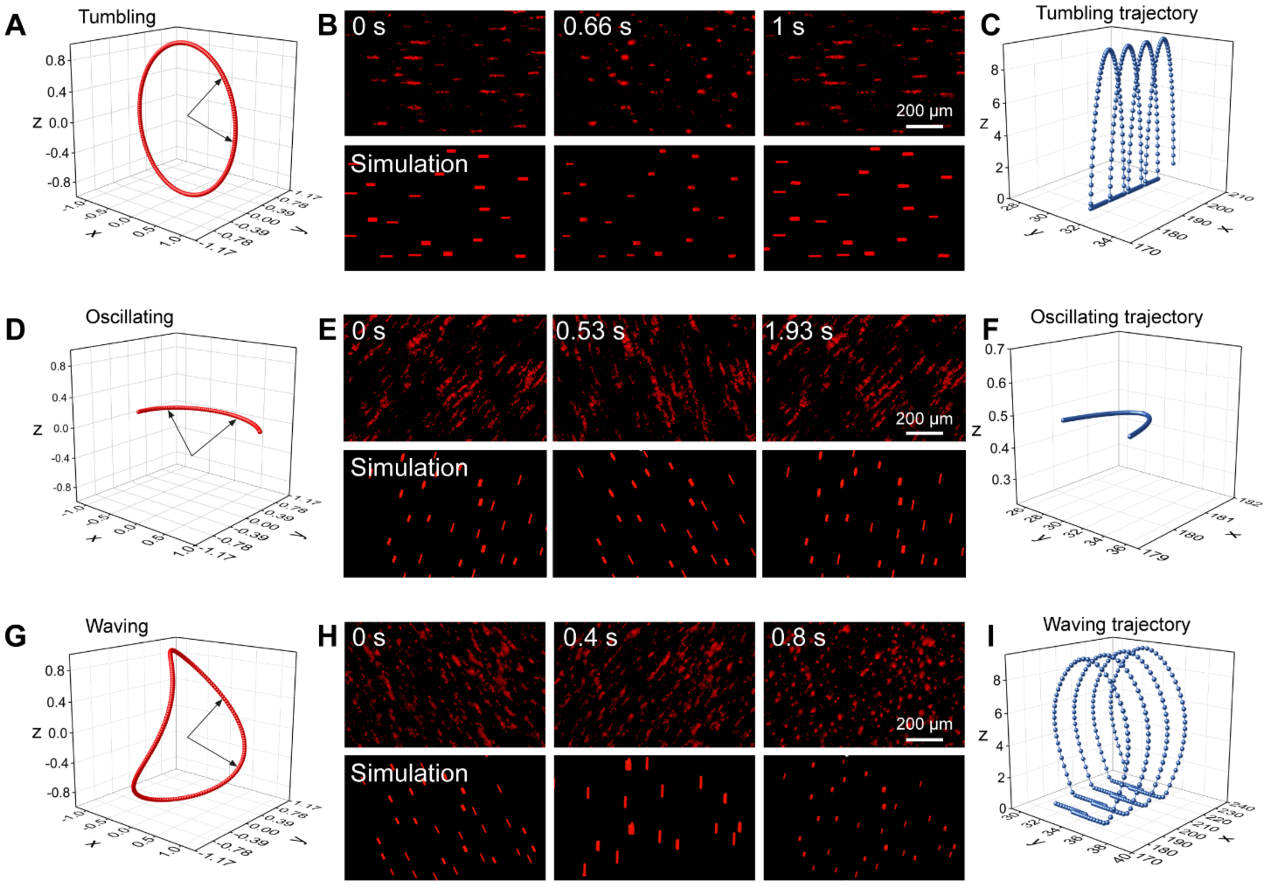

Fig. S17. Locomotion of probiotic microrobot swarm. (**A**), (**D**) and (**G**) The evolution diagram of the input magnetic field in one cycle period. (**B**), (**E**) and (**H**) Probiotic microrobot swarm performs tumbling, oscillating and waving motion under magnetic field (8 mT, 0.5 Hz) manipulation. (**C**), (**F**) and (**I**) Theoretical simulation trajectory of microrobot swarm with tumbling, oscillating and waving mode.

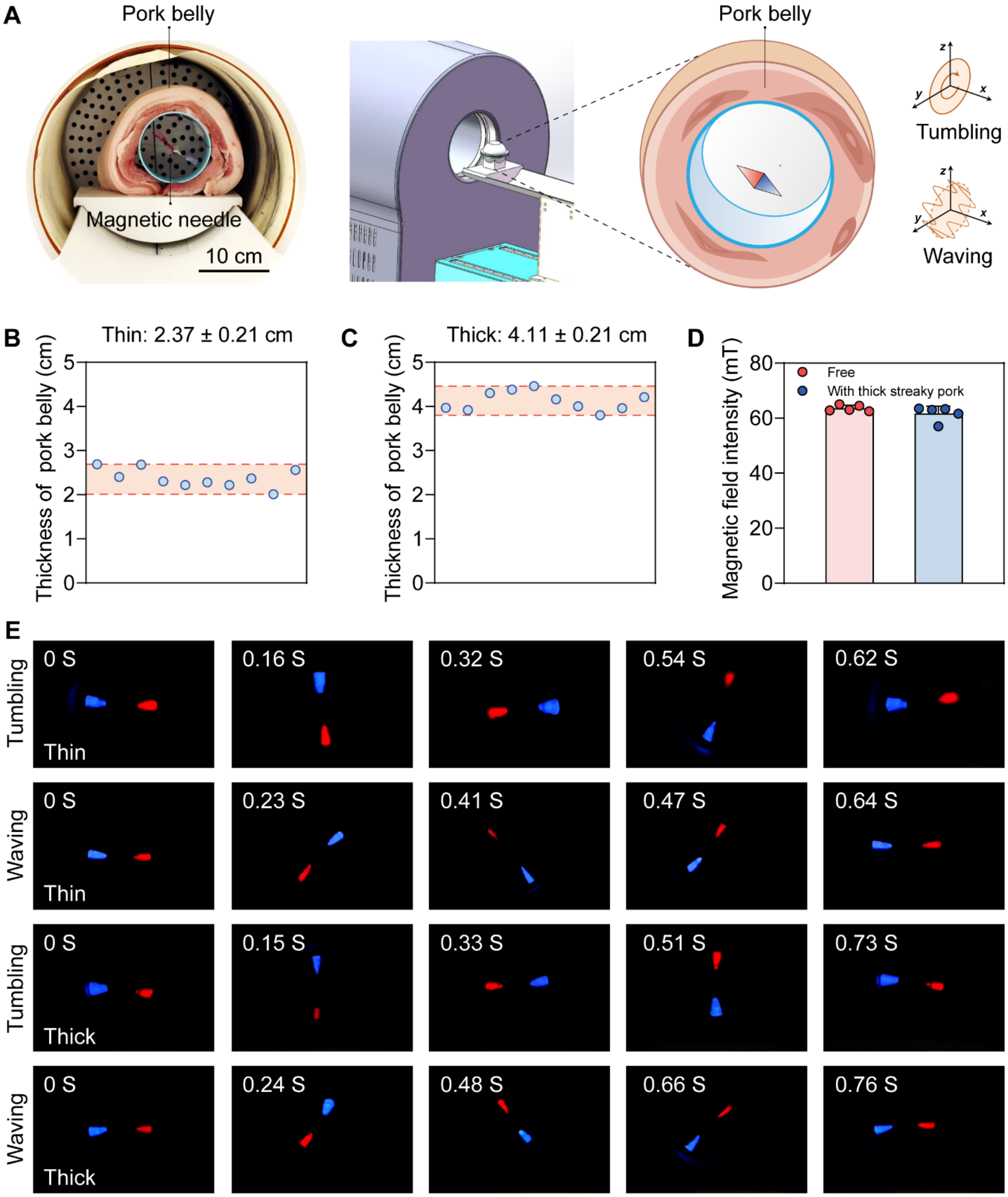

Fig. S18. Characterization and validation of the hybrid magnetic control system for deep-tissue actuation. (**A**) Photograph and schematic illustration of the multi-mode magnetic control platform integrating both permanent and electromagnetic fields. (**B**) Thickness measurements of thin porcine tissue models at ten different locations. (**C**) Thickness measurements of thick porcine

tissue models at ten random locations. (**D**) Magnetic field intensity measured at five identical positions beneath thin and thick tissue layers, respectively. (**E**) Locomotion of a magnetic needle (with red and blue markers at both ends) inside thin and thick tissue models under tumbling and waving magnetic fields (79 mT, 1 Hz), respectively.

We compiled and compared representative studies reporting magnetic actuation depths in soft-tissue or tissue-mimicking media (table S4). The comparison shows that the effective penetration depth of dynamic magnetic actuation is typically limited to about 4 cm. This highlights the intrinsic limitation faced by most existing magnetic control systems. To address this challenge, we developed a 5 DOFs (degrees of freedom) magnetic control platform that integrates both permanent and electromagnetic components, enabling the generation of programmable three-dimensional magnetic fields with enhanced depth penetration and spatial uniformity (fig. S18 and movie S6). We experimentally validated its performance by comparing the motion of a small magnetic needle embedded in two layers of porcine tissue models: a thin-layer group (2.01-2.69 cm) and a thick-layer group (3.80-4.46 cm) (fig. S18, A to E). The results demonstrated that the needles could still achieve stable tumbling and waving modes through tissue thicknesses up to 4.46 cm, which is comparable to the average abdominal wall thickness in humans. These findings suggest that our system provides sufficient field strength for magnetic actuation at clinically relevant depths, thereby expanding the applicability of swarm-based microrobots to deep-seated tumors such as those in the liver or pancreas.

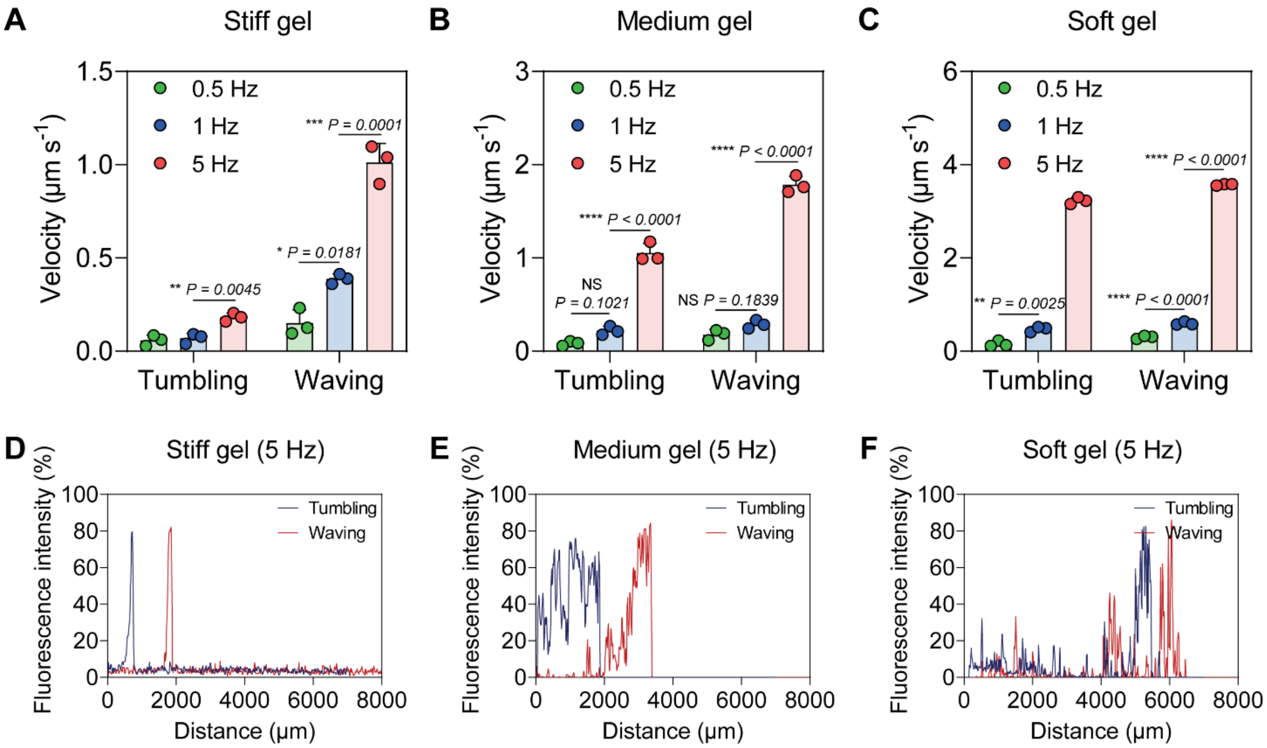

Fig. S19. Collagen gels penetration of the probiotic microrobot swarm. (**A**) to (**C**) The velocity of probiotic microrobot swarm in collagen gels with different stiffnesses (soft, medium and stiff) in the tumbling and waving mode, respectively (n = 3; means ± SD). (**D**) to (**F**) Fluorescence analysis of the distribution of probiotic microrobot swarm in collagen gels in the tumbling and waving mode, respectively.

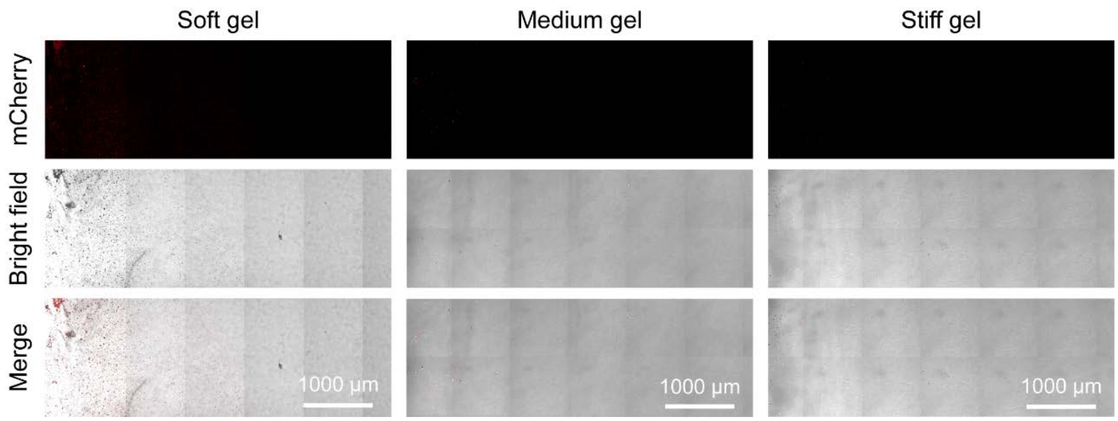

Fig. S20. Collagen gels penetration of the probiotic microrobot swarm without magnetic field. Fluorescence images and bright-field optical microscopy images of collagen gel regions after

incubation (30 min) with probiotic microrobot swarm without magnetic field. mCherry indicates probiotic microrobots.

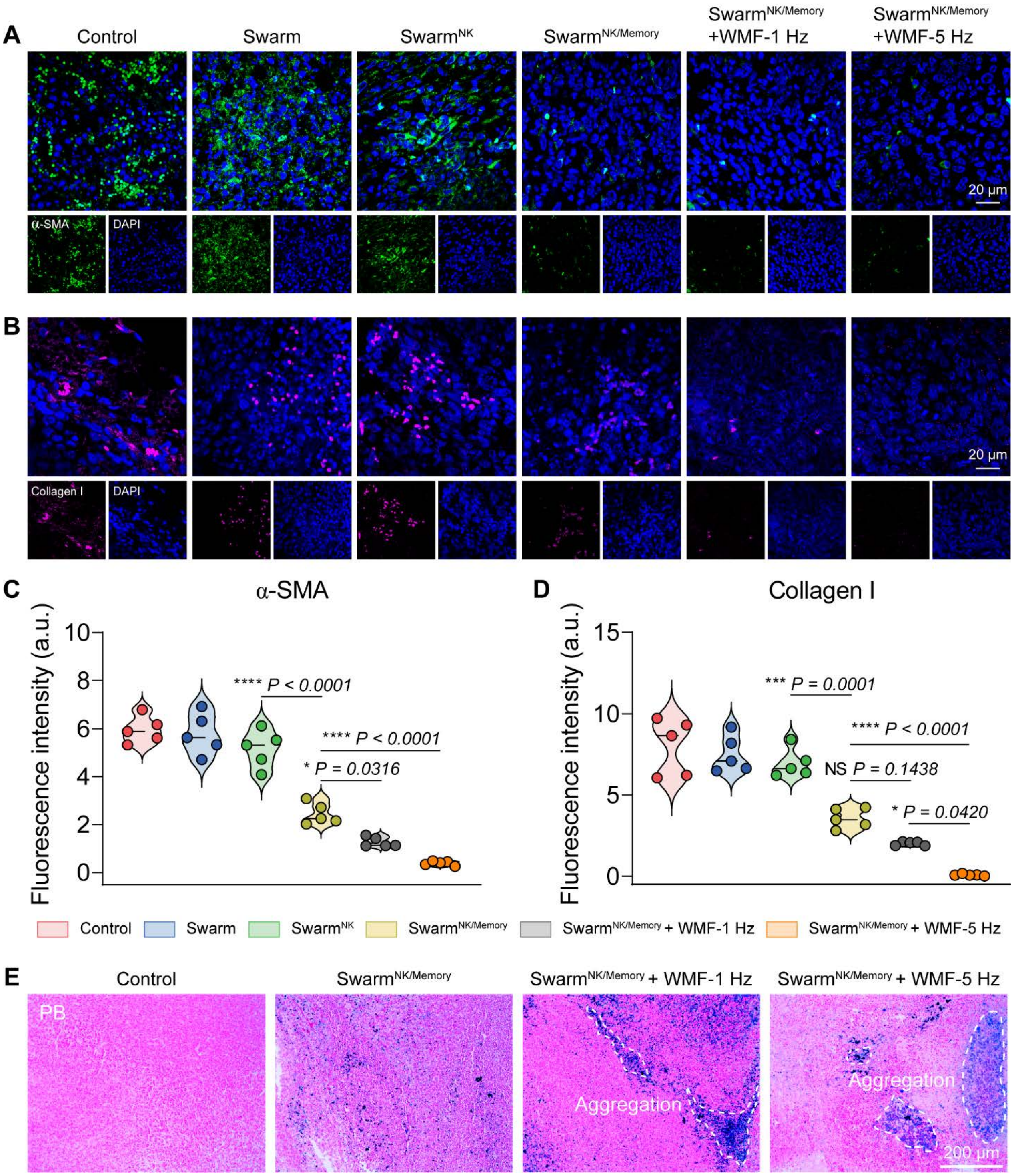

Fig. S21. Fluorescence images of tumor sections in different treatment groups. (**A**) Immunofluorescence staining of α-SMA in tumor sections after treatment. (**B**) Immunofluorescence staining of Collagen I in tumor sections after treatment. α-SMA (green), and Collagen I (purple) were stained with the related antibodies. Nucleus was stained with DAPI (blue). (**C**) and (**D**) Quantitative α-SMA and Collagen I fluorescence in tumors of 4T1 tumor-bearing mice after different treatments (n = 5; means ± SD). (**E**) Prussian blue (PB) staining in tumor sections after different treatments.

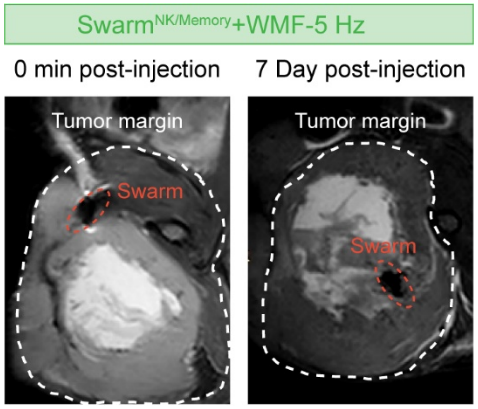

Fig. S22. The stability of probiotic microrobot swarm assessed by MRI. MRI was performed on 4T1 tumor-bearing mice after peritumoral injection of probiotic microrobots and again after WMF actuation (74 mT, 5 Hz, 60 min) following injection. The elliptical region denotes the T2 signal corresponding to the microrobot swarm.

To assess whether the swarm structure remained intact during 3D waving locomotion, we performed MRI immediately after peritumoral injection of the probiotic microrobots and again on day 7 after magnetic actuation (74 mT, 5 Hz, 60 min) in 4T1 tumor-bearing mice. A consistent, well-defined T2 signal corresponding to the microrobot swarm was observed at both time points, indicating that the swarm retained its overall structural integrity during actuation (fig. S22).

That said, the heterogeneous and dynamic nature of the tumor microenvironment, including local variations in tissue density, interstitial flow, and immune activity, may lead to partial disaggregation of the swarm into smaller sub-clusters during locomotion. Importantly, such fragmentation does not impair the microrobots' ability to penetrate the tumor tissue, as the resultant

sub-clusters remain well within the effective propulsion and penetration range of the system (fig. S23E).

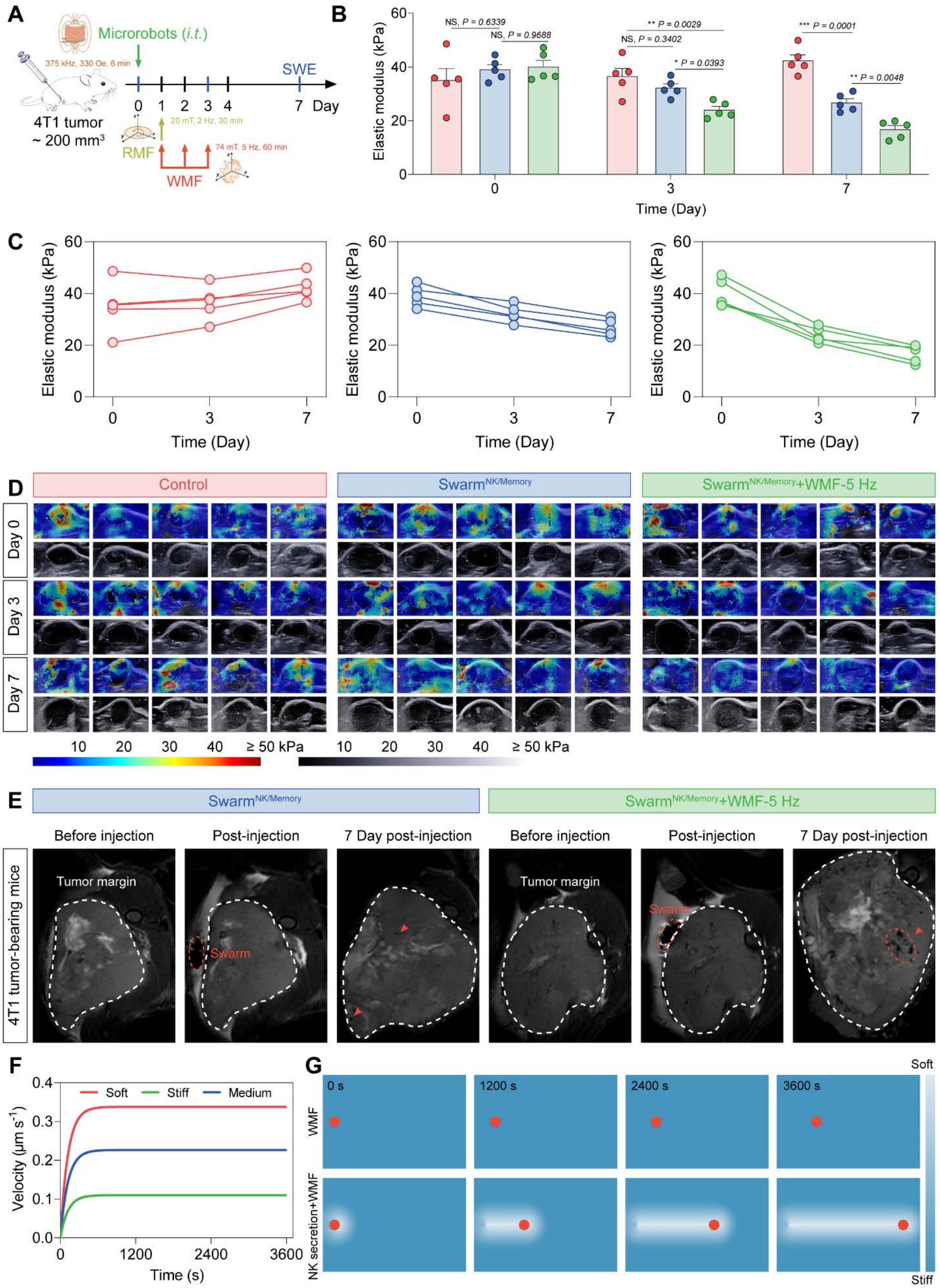
A
Microrobots (i.t.)
375 kHz, 330 Oe, 6 min
4T1 tumor
~ 200 mm3
SWE
0 1 2 3 4 7 Day
20 mT, 2 Hz, 30 min
RMF
74 mT, 5 Hz, 60 min
WMF
B
Elastic modulus (kPa)
NS, P = 0.6339
NS, P = 0.9688
** P = 0.0029
NS, P = 0.3402
* P = 0.0393
*** P = 0.0001
** P = 0.0048
Time (Day)
C
Elastic modulus (kPa)
Time (Day)
D
Control
SwarmNK/Memory
SwarmNK/Memory+WMF-5 Hz
Day 0
Day 3
Day 7
10 20 30 40 ≥ 50 kPa
10 20 30 40 ≥ 50 kPa
E
SwarmNK/Memory
SwarmNK/Memory+WMF-5 Hz
Before injection
Post-injection
7 Day post-injection
4T1 tumor-bearing mice
Tumor margin
Swarm
F
Soft
Stiff
Medium
Velocity (μm s-1)
Time (s)
G
0 s
1200 s
2400 s
3600 s
WMF
NK secretion+WMF
Soft
Stiff

Fig. S23. Dynamic ultrasound shear wave elastography (SWE) after probiotic microrobot swarm therapy and tumor tissue penetration assessed by magnetic resonance imaging (MRI). (**A**) Schematic illustration of SWE examinations performed at different time points (0, 3, and 7 days) after probiotic microrobot swarm therapy. (**B**) and (**C**) Dynamic elastic moduli of 4T1 tumor tissues measured at 0, 3, and 7 days following different treatments (n = 5, mean ± SEM). (**D**) Representative SWE images of the 4T1 tumors from different treatment groups (n = 5). The dashed-line coil region represents the tumor target area detected by ultrasound elastography. (**E**) Tumor tissue penetration of the probiotic microrobot swarm assessed by MRI. MRI was performed on 4T1 tumor-bearing mice before peritumoral injection of probiotic microrobots and again after WMF actuation (74 mT, 5 Hz, 60 min) following injection. The elliptical region denotes the T2 signal corresponding to the microrobot swarm. (**F**) Theoretical prediction of microrobot locomotion velocity as a function of matrix stiffness under WMF actuation. Simulations were performed in soft, medium, and stiff tumor-like environments. A higher matrix stiffness results in a lower terminal velocity, illustrating the strong dependence of magnetic propulsion efficiency on local mechanical resistance. (**G**) Time-lapse simulation of microrobot penetration in heterogeneous tumor matrices. Top row: WMF alone, where the microrobot experiences a constant stiff microenvironment and shows minimal forward displacement over 3600 s. Bottom row: NK-secretion combined with WMF, where enzymatic remodeling produces a trailing softening gradient (white region) that progressively expands and facilitates deeper microrobot penetration. The color bar represents local stiffness (white represents soft, dark blue represents stiff). Statistical analysis was performed using One-way ANOVA with Tukey's post hoc test for (**B**). NS: $P > 0.05$.

Because tissue softening is a biologically regulated process occurring on a multi-day timescale, whereas mechanical penetration by microrobots takes place immediately after magnetic actuation, the two processes inherently occur in different temporal windows. To capture their dynamic evolution, we used ultrasound SWE to quantify stiffness changes (fig. S23A) and MRI/*in vivo* fluorescence imaging to track microrobot penetration across matched time points. SWE revealed that the elastic modulus of 4T1 tumors progressively increased in the control group but continuously decreased in the $\text{Swarm}^{\text{NK/Memory}}$ group, with a 60.44% reduction in the $\text{Swarm}^{\text{NK/Memory}}$ + WMF-5 Hz group on day 7 compared with control (fig. S23, B to D). To evaluate the subsequent penetration behavior, MRI scans obtained before injection and on day 7

demonstrated that microrobots in the Swarm$^{NK/Memory}$ + WMF-5 Hz group migrated substantially deeper into the tumor compared with the Swarm$^{NK/Memory}$ group (fig. S23E). Complementary *in vivo* fluorescence imaging further confirmed progressive intratumoral accumulation of mCherry-labeled microrobots, and *ex vivo* fluorescence analysis validated their final spatial distribution (fig. S24).

In addition to experimental evidence, our biophysical simulations further support the proposed softening-penetration feedback loop. As shown in fig. S23F, microrobot locomotion speed is strongly dependent on the local mechanical stiffness of the matrix: motion is fastest in soft regions, slower in medium-stiffness regions, and significantly impeded in stiff regions. These stiffness-dependent velocity differences establish the mechanical basis for why localized softening facilitates accelerated forward movement. Fig. S23G visualizes this process in a dynamic tumor-like environment. Under WMF actuation alone, the microrobot exhibits minimal displacement over time due to uniformly stiff surroundings. In contrast, when NK-secreted factors gradually soften the microenvironment, a propagating low-stiffness corridor forms behind and around the swarm, enabling progressively deeper wavefront penetration as the softening region expands. The spatiotemporal pattern in the simulation mirrors our *in vivo* observations, demonstrating how biologically driven ECM softening creates a permissive mechanical pathway that amplifies penetration depth.

Taken together, although real-time intravital stiffness mapping during locomotion is currently technically infeasible, our temporally aligned multimodal data (showing SWE-defined localized softening followed by MRI-verified deeper penetration) and stiffness-dependent motion simulations provide coherent dynamic evidence supporting the softening-penetration feedback loop *in vivo*.

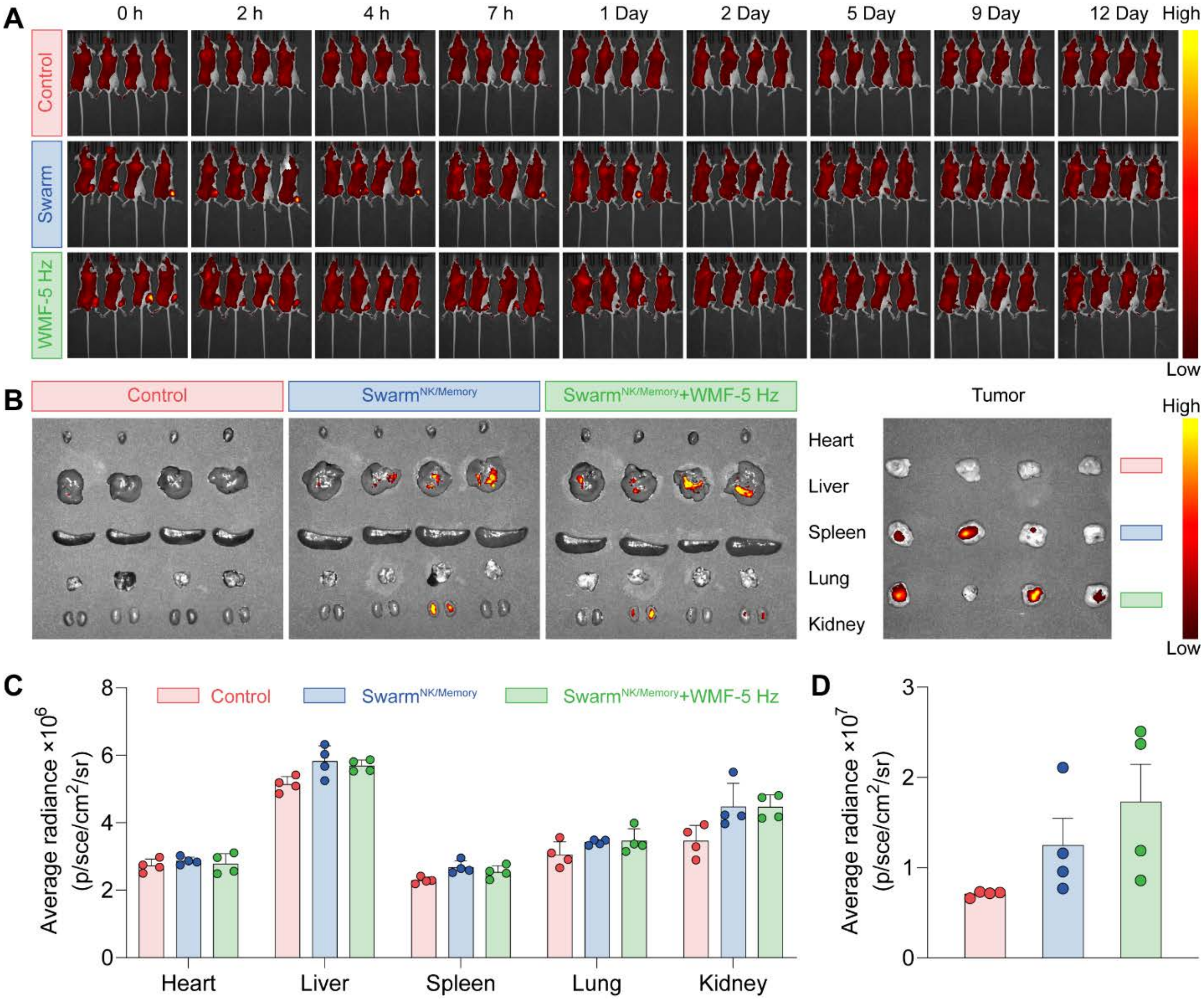

Fig. S24. ***In vivo*** **fluorescence imaging of 4T1 tumor-bearing mice at different time points (0 h, 2 h, 4 h, 7 h, 1 day, 2 days, 5 days, 9 days, and 12 days) after receiving intratumoral injection of probiotic microrobots.** (**A**) *In vivo* fluorescence images of 4T1 tumor-bearing mice at the indicated time points after intratumoral injection of probiotic microrobots with or without WMF actuation (n = 4 per group). (**B**) Fluorescent images of tumors and major organs collected from treated tumor-bearing mice 12 days post-injection (n = 4 per group). (**C**) Quantitative fluorescence intensity of mCherry in major organs 12 days post-injection (n = 4, mean ± SEM). (D) Quantitative fluorescence intensity of mCherry in tumors 12 days post-injection (n = 4, mean ± SEM).

To further demonstrate its significance in the therapeutic loop, we performed real-time dynamic monitoring of mCherry fluorescence in 4T1 tumor-bearing mice using an *in vivo* imaging system.

Following probiotic microrobots administration, mCherry fluorescence levels in the tumors remained high during the first 24 h and showed a moderate decline by day 2 (fig. S24A). Importantly, detectable mCherry signals persisted within the tumor region throughout the subsequent 10-day monitoring period. On day 12, *ex vivo* fluorescence imaging and quantitative analysis of tumors confirmed sustained mCherry expression (fig. S24B), verifying the *in vivo* imaging results. Compared with the control group, the Swarm$^{NK/Memory}$ + WMF-5 Hz group also exhibited mCherry fluorescence in the liver and kidney, with increases of approximately 1.10-fold and 1.29-fold, respectively. By contrast, the other organs, such as the heart, spleen, and lung showed no significant enrichment (fig. S24, B to D). Collectively, these findings demonstrate that the mCherry module provides meaningful real-time imaging feedback both *in vitro* and *in vivo*, and that the genetic memory remains active throughout the 12-day observation period.

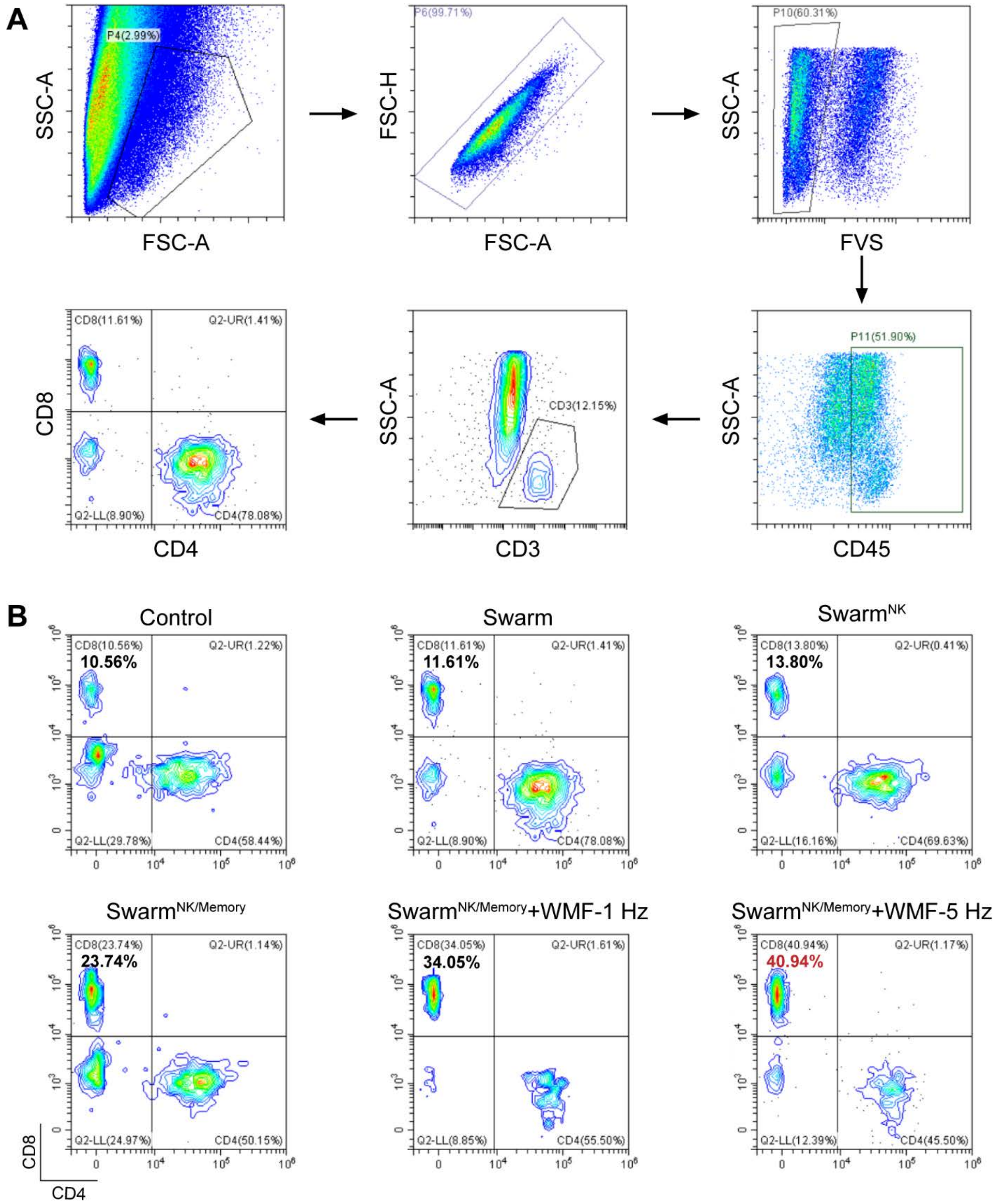

Fig. S25. Analysis of the antitumor immune responses triggered by probiotic microrobots. (**A**) Gating strategy for the detection of $CD8^+$ and $CD4^+$ T cells. (**B**) Flow analysis statistical results of $CD8^+$ T cells and $CD4^+$ T cells in tumor tissue in six treatment groups.

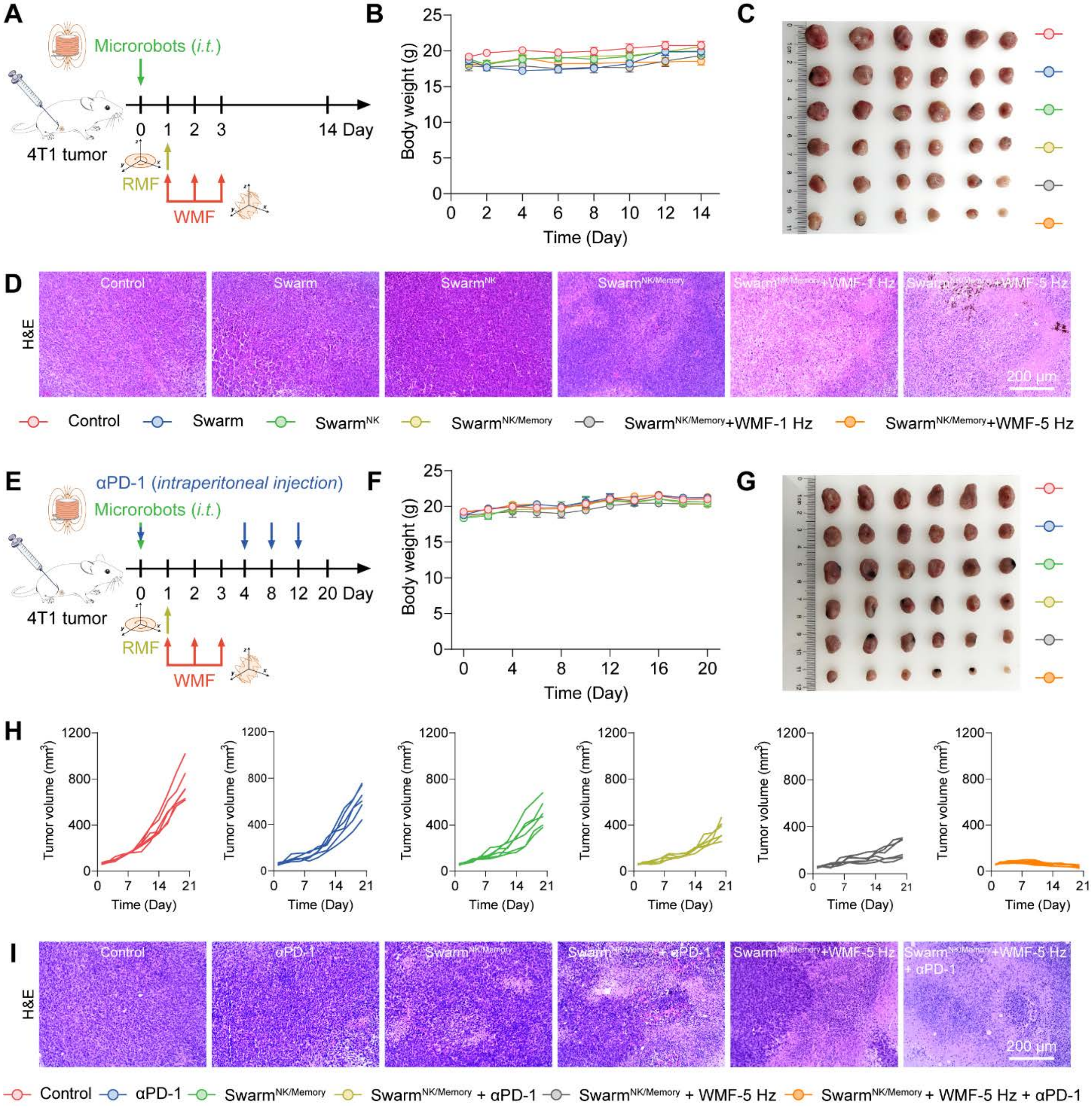

Fig. S26. Tumor regulation effect of probiotic microrobots. (**A**) Timeline of probiotic microrobots treatments. (**B**) Body weight after different treatments (n = 6; means ± SEM). (**C**) Image of tumors for every BALB/c mouse in different treatment groups (n = 6). (**D**) H&E staining of tumor slices in different treatment groups. (**E**) Timeline of probiotic microrobots treatments under magnetic field. The αPD-1 was injected intraperitoneally at a dose of 100 μg per mouse every 4 days, for a total of 4 injections. (**F**) Body weight after different treatments (n = 6; means

± SEM). (**G**) Image of tumors for every BALB/c mouse in different treatment groups (*n* = 6). (**H**) Growth curve of tumor volumes for every BALB/c mouse in different treatment groups (*n* = 6; means ± SEM). (**I**) H&E staining of tumor slices in different treatment groups.

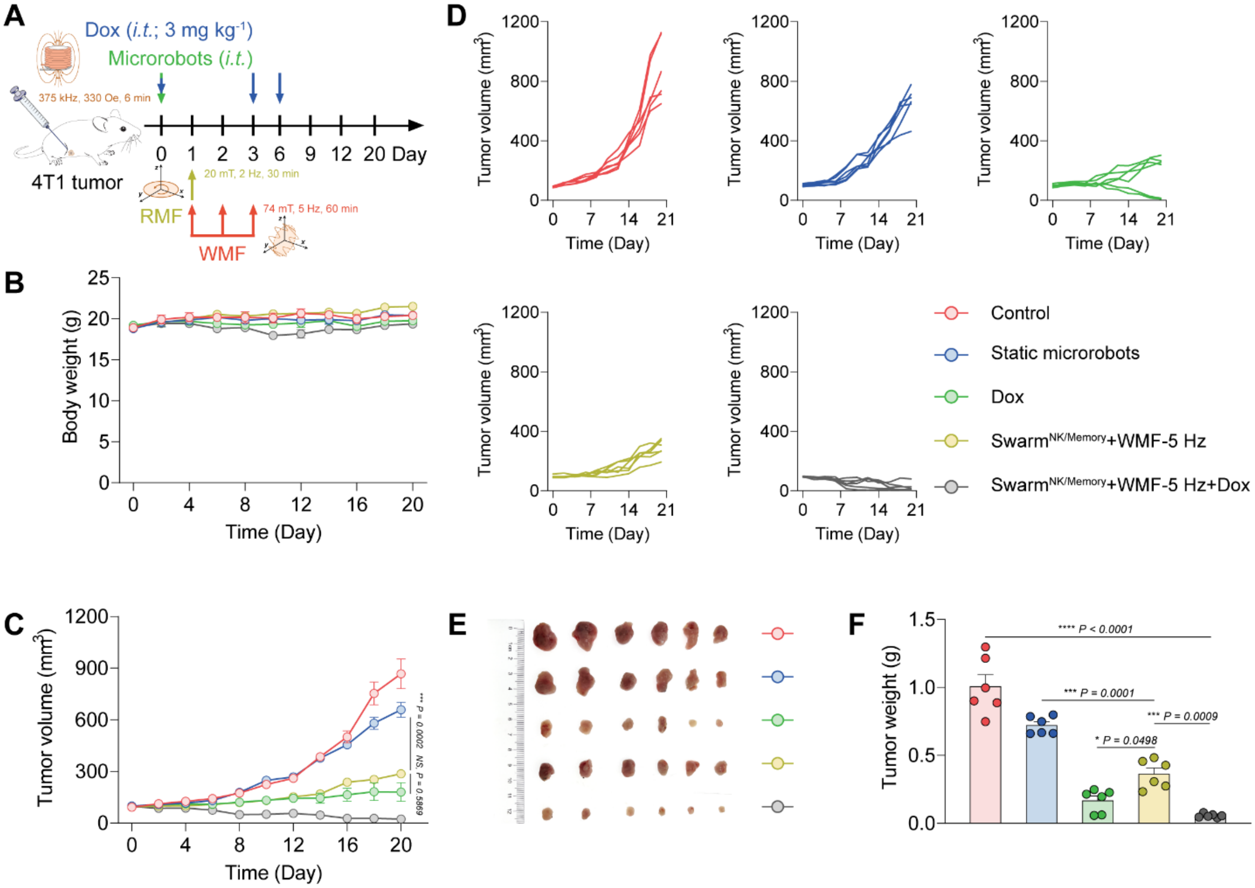

Fig. S27. Antitumor efficacy of the memory-encoded microrobot swarm compared to the therapeutic drug Dox and static microrobots in the 4T1 tumor model. (**A**) Timeline of probiotic microrobot swarm treatments in 4T1 tumor model. Dox treatment was administered intratumorally at 3 mg kg^{-1} per mouse on day 0, 3, and 6. Static microrobots were prepared by removing bacterial flagella using 0.5 M acetic acid, followed by MNPs conjugation. (**B**) Body weight changes after different treatments (*n* = 6; means ± SEM). (**C**) Tumor volumes of 4T1 tumor-bearing mice treated in different groups (*n* = 6; means ± SEM). (**D**) Tumor growth curve for each BALB/c mouse in different treatment groups (*n* = 6). (**E**) Image of 4T1 tumors from each BALB/c mouse with different treatment groups (*n* = 6). (**F**) Tumor weight of every BALB/c mouse with

different treatment strategies ($n = 6$; means ± SEM). Statistical analysis was performed using One-way ANOVA with Tukey's post hoc test for (**C**) and (**F**). NS: $P > 0.05$.

The microrobot swarms actuated at WMF-5 Hz achieved 66.94% tumor growth inhibition, which was higher compared to static microrobots (24.10% tumor growth inhibition). Microrobot swarms exhibited antitumor efficacy comparable to that of free Dox treatment (79.13% tumor growth inhibition). The combination of Swarm$^{NK/Memory}$ + WMF therapy with Dox demonstrated the most potent antitumor efficacy (97.29% tumor growth inhibition) by day 20. Moreover, no significant body weight loss was observed in the static microrobots and Swarm$^{NK/Memory}$ + WMF therapy groups. In contrast, a transient decline in body weight was evident during the first 8 days of free Dox treatment and Swarm$^{NK/Memory}$ + WMF therapy with Dox, indicating the presence of drug toxicity.

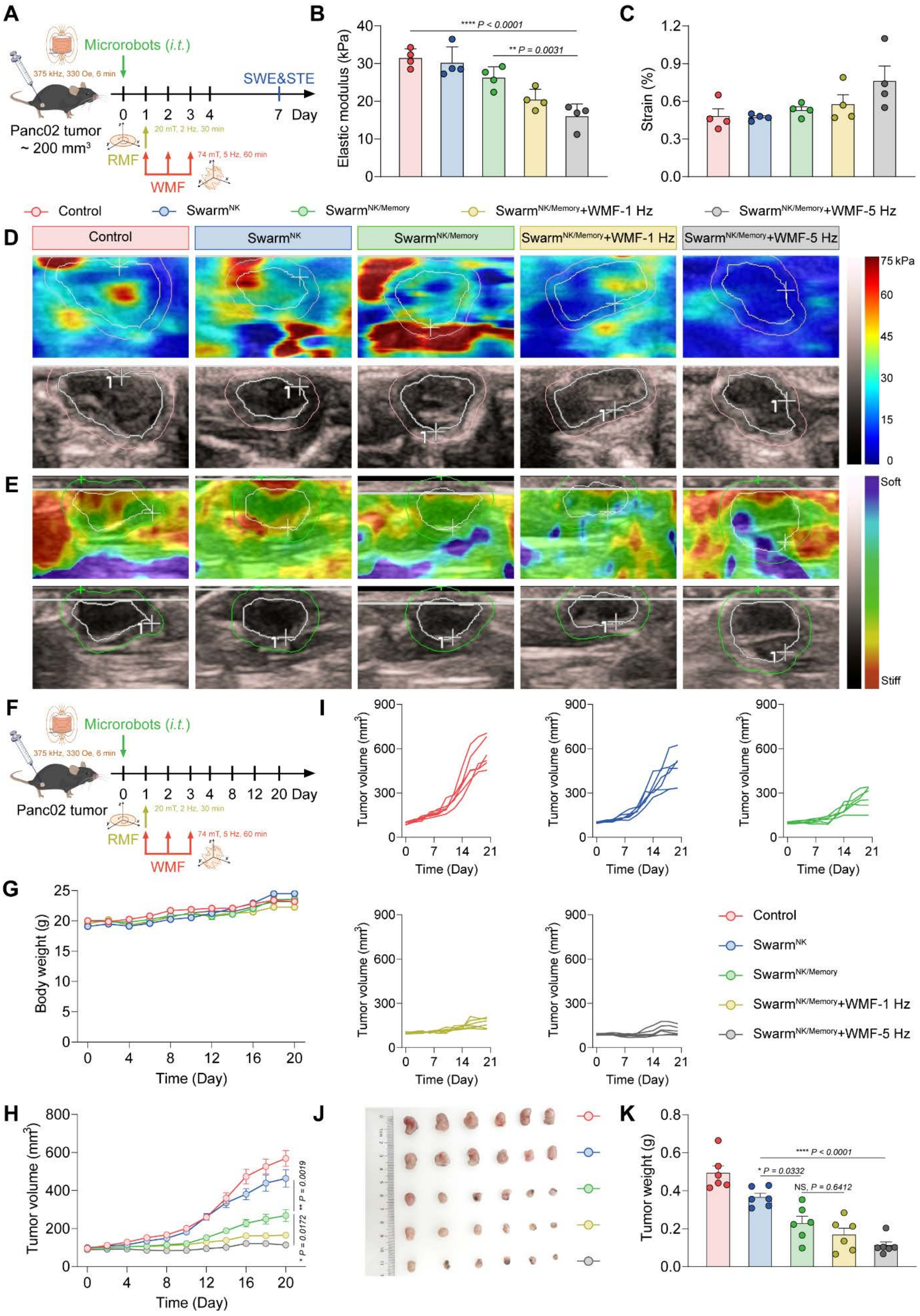
A
Microrobots (i.t.)
375 kHz, 330 Oe, 6 min
SWE&STE
0 1 2 3 4 7 Day
Panc02 tumor
~ 200 mm³
20 mT, 2 Hz, 30 min
RMF
74 mT, 5 Hz, 60 min
WMF
B
Elastic modulus (kPa)
**** P < 0.0001
** P = 0.0031
C
Strain (%)
Control
Swarm^NK
Swarm^NK/Memory
Swarm^NK/Memory+WMF-1 Hz
Swarm^NK/Memory+WMF-5 Hz
D
75 kPa
E
Soft
Stiff
F
Microrobots (i.t.)
375 kHz, 330 Oe, 6 min
0 1 2 3 4 8 12 20 Day
Panc02 tumor
20 mT, 2 Hz, 30 min
RMF
74 mT, 5 Hz, 60 min
WMF
G
Body weight (g)
Time (Day)
H
Tumor volume (mm³)
Time (Day)
* P = 0.0172
** P = 0.0019
I
Tumor volume (mm³)
Time (Day)
J
K
Tumor weight (g)
* P = 0.0332
NS, P = 0.6412
**** P < 0.0001

Fig. S28. Antitumor efficacy of the memory-encoded microrobot swarm in the Panc02 tumor model. (**A**) Schematic illustration of ultrasound shear wave elastography (SWE) and strain elastography (STE) examinations after probiotic microrobot swarm therapy. (**B**) Elastic modulus of the Panc02 tumors in different treatment groups (n = 4; means ± SEM). (**C**) Quantitative results of strain from strain elastography images of Panc02 tumors in different groups (n = 4; means ± SEM). (**D**) and (**E**) Representative SWE and STE images of the Panc02 tumors from different treatment groups. The area marked by the real coil represents the tumor target area detected by ultrasound elastography. (**F**) Timeline of probiotic microrobot swarm treatments in Panc02 tumor model. (**G**) Body weight after different treatments (n = 6; means ± SEM). (**H**) Tumor volumes of Panc02 tumor-bearing mice treated in different groups (n = 6; means ± SEM). (**I**) Growth curve of tumor volumes for every C57 mouse in different treatment groups (n = 6). (**J**) Image of Panc02 tumors for every C57 mouse in different treatment groups (n = 6). (**K**) Tumor weight of every C57 mouse with different treatment strategies (n = 6; means ± SEM). Statistical analysis was performed using One-way ANOVA with Tukey's post hoc test for (**B**), (**H**) and (**K**). NS: $P > 0.05$.

Accordingly, we extended our study to a pancreatic cancer (Panc02) model, which features a dense and fibrotic ECM environment. The stiffness of Panc02 tumor tissue was assessed using ultrasound SWE and ultrasound strain elastography (STE) examinations at 7 days post-treatment (fig. S28A). Compared to the control group, the elastic moduli in the Swarm$^{NK/Memory}$ + WMF-5 Hz group were significantly decreased by 49.08% (fig. S28, B and D). Additionally, the strain in the Swarm$^{NK/Memory}$ + WMF-5 Hz group increased 1.58-fold compared to the control group (fig. S28, C and E). The antitumor efficacy of the microrobot swarm in the Panc02 pancreatic model was further studied as illustrated in fig. S28F. The body weights of mice in each group remained stable throughout the treatment period (fig. S28G). Compared to the control groups, the Swarm$^{NK/Memory}$ under the WMF treatment demonstrated an efficient antitumor effect (fig. S28, H to K). Microrobot swarms actuated at WMF-5 Hz achieved 80% tumor growth inhibition, which was higher compared to those at WMF-1 Hz (70.98% tumor growth inhibition). Collectively, these results demonstrate that Swarm$^{NK/Memory}$ microrobots remain effective in stiff, ECM-rich pancreatic tumors.

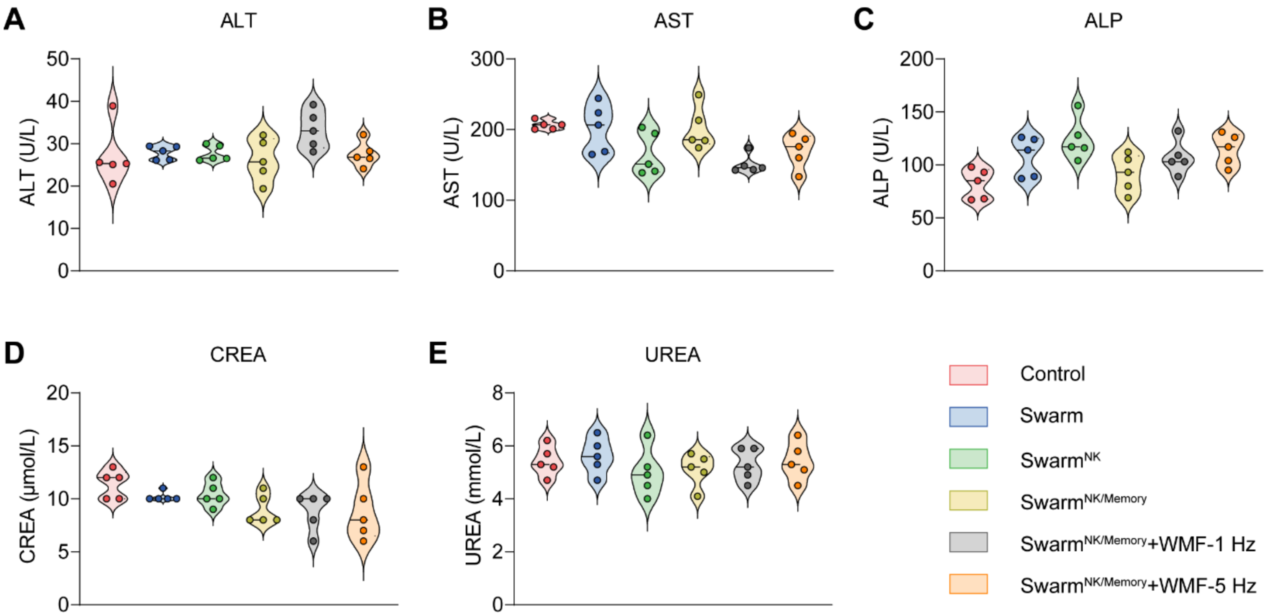

Fig. S29. The blood chemistry panel on day 14 after administration. Liver function markers: (alanine aminotransferase (ALT), aspartate aminotransferase (AST), alkaline phosphatase (ALP); kidney function markers: creatinine (CREA), urea nitrogen (UREA); (n = 5; means ± SD).

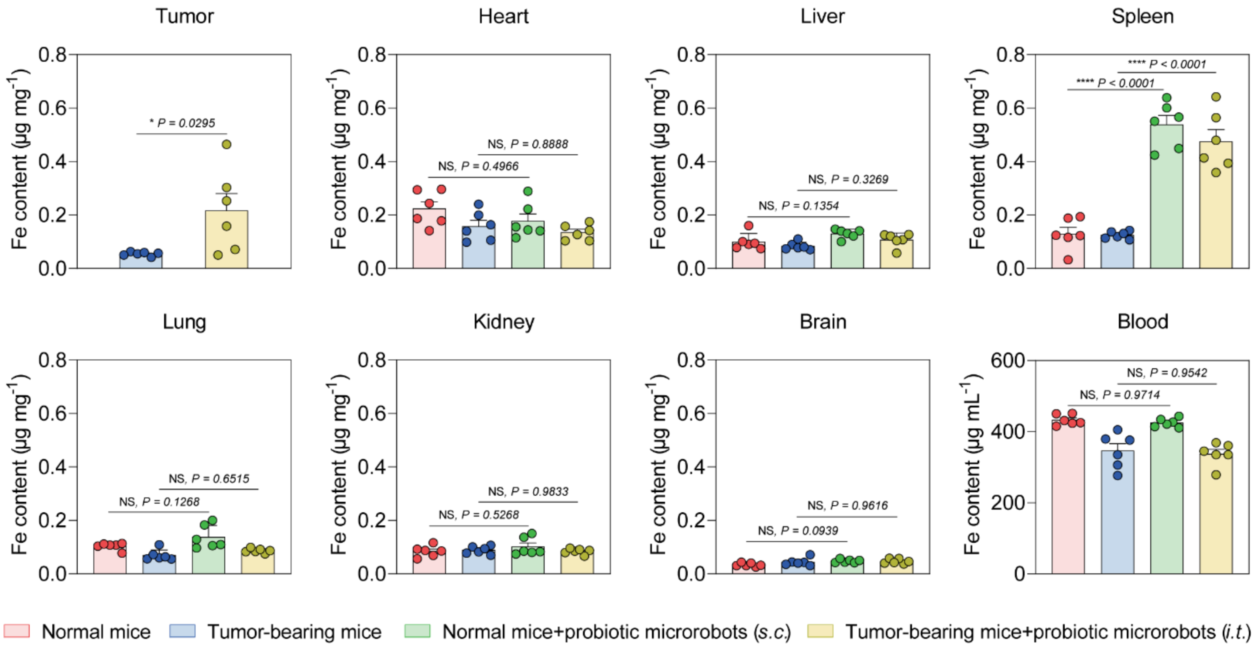

Fig. S30. Iron content in the heart, liver, spleen, lung, kidney, brain, tumor tissue and blood in different treatment groups after 21 days (n = 6; means ± SEM). subcutaneous injection (*s.c.*),

intratumoral injection (*i.t.*). Statistical analysis was performed using One-way ANOVA with Tukey's post hoc test. NS: $P > 0.05$.

To investigate the long-term fate of the magnetic nanoparticles, probiotic microrobots were administered either subcutaneously to healthy mice or intratumorally to 4T1 tumor-bearing mice. On day 21 post-treatment, iron ion distribution in tumors, major organs (heart, liver, spleen, lung, kidney, and brain), and blood were quantified via ICP-MS. The results showed that intratumoral injection led to predominant accumulation of iron particles within the tumor. Moreover, no significant differences in iron content were observed in major organs (heart, liver, lung, kidney, and brain) and blood among healthy mice, tumor-bearing mice, or mice receiving either subcutaneous or intratumoral microrobot administration (fig. S30). These findings indicate that the magnetic nanoparticles were not metabolized through these organs and blood. However, iron levels in the spleen increased by approximately 4.15-fold in healthy mice receiving subcutaneous injection and 3.80-fold in tumor-bearing mice receiving intratumoral injection on day 21, demonstrating that the magnetic nanoparticles primarily accumulate in the spleen. As a key metabolic organ, the spleen appears to play a dominant role in processing and clearing magnetic nanoparticles in mice.

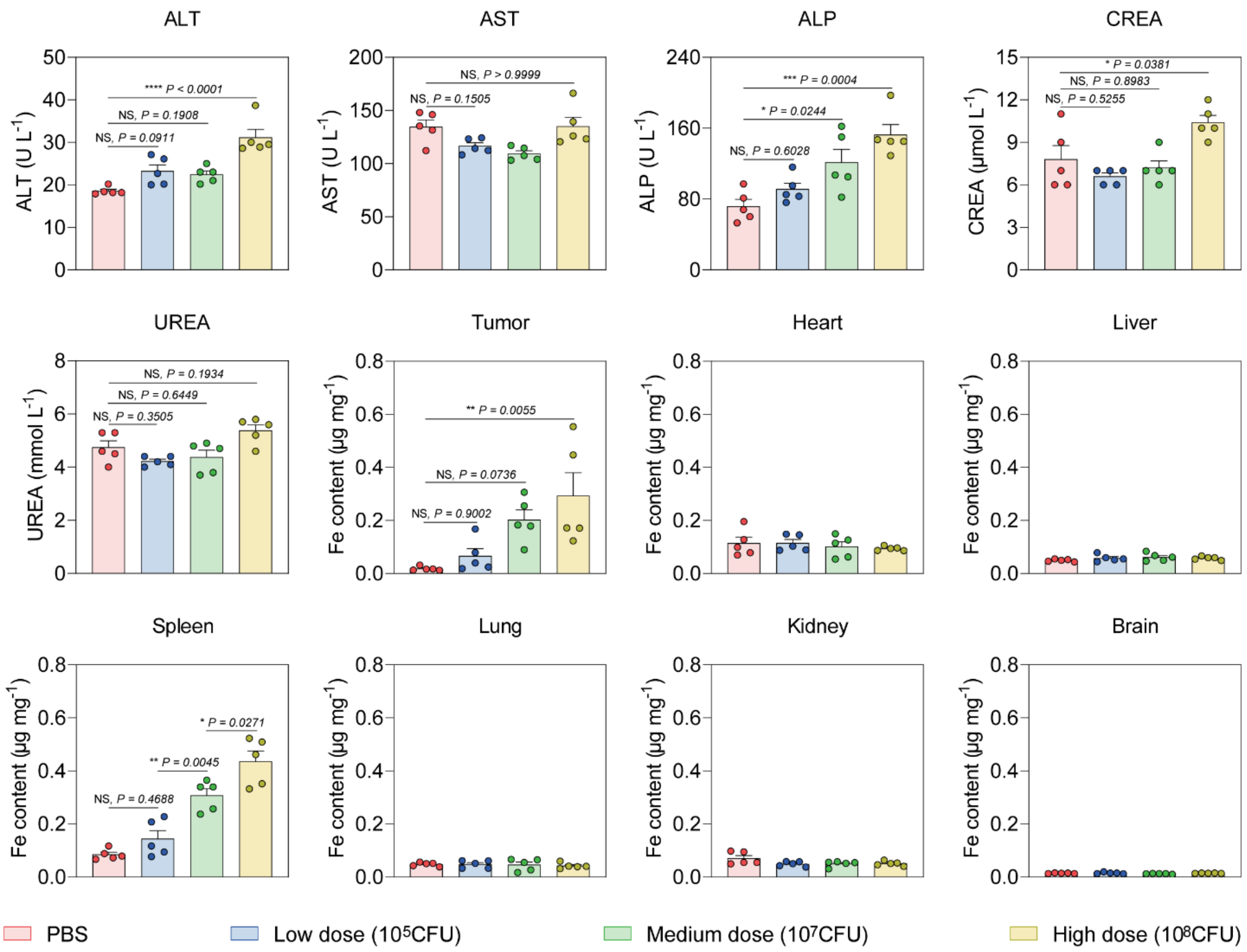

Fig. S31. *In vivo* safety evaluation of intratumoral injection of different doses of probiotic microrobots (Low dose: 10^5 CFU, Medium dose: 10^7 CFU, High dose: 10^8 CFU) followed by WMF treatment. The blood chemistry panel on day 21 after administration. Liver function markers: (alanine aminotransferase (ALT), aspartate aminotransferase (AST), alkaline phosphatase (ALP); kidney function markers: creatinine (CREA), urea nitrogen (UREA); (n = 5; means ± SEM). Iron content in heart, liver, spleen, lung, kidney, brain, and tumor tissue in different treatment groups after 21 days (n = 5; means ± SEM). Statistical analysis was performed using One-way ANOVA with Tukey's post hoc test. NS: P ＞ 0.05.

Additionally, a dose-escalation study (low dose: 10^5 CFU, medium dose: 10^7 CFU, high dose: 10^8 CFU) was conducted in 4T1 tumor-bearing mice. After 20 days of treatment, blood chemistry analysis showed that the liver and kidney function markers, including ALT, AST, ALP, CREA, and UREA, remained within normal physiological ranges across all doses. The medium group (10^7 CFU), which was the dose we utilized in the study, showed the good biocompatibility according

to the blood chemistry analysis. Additionally, body weight also remained stable in both the Panc02 and 4T1 tumor models (fig. S31). To evaluate the biodistribution of magnetic nanoparticles in the dose-escalation study, iron content in major organs (heart, liver, spleen, lung, kidney, and brain), blood, and tumors were quantified on day 21 post-treatment. Intratumoral injection resulted in predominant retention of MNPs within the tumor, with no significant differences in iron levels in major organs (heart, liver, lung, kidney, and brain) and blood across different microrobot doses (fig. S31). These results further demonstrate that the magnetic nanoparticles were not metabolized through these organs. However, the iron accumulation in the spleen increased in a dose-dependent manner, consistent with its role as a primary metabolic organ (fig. S31). Overall, the stable body weight, normal blood biochemical profiles, and controlled biodistribution of magnetic nanoparticles collectively confirm that the microrobot doses used in this study fall within a safe range.

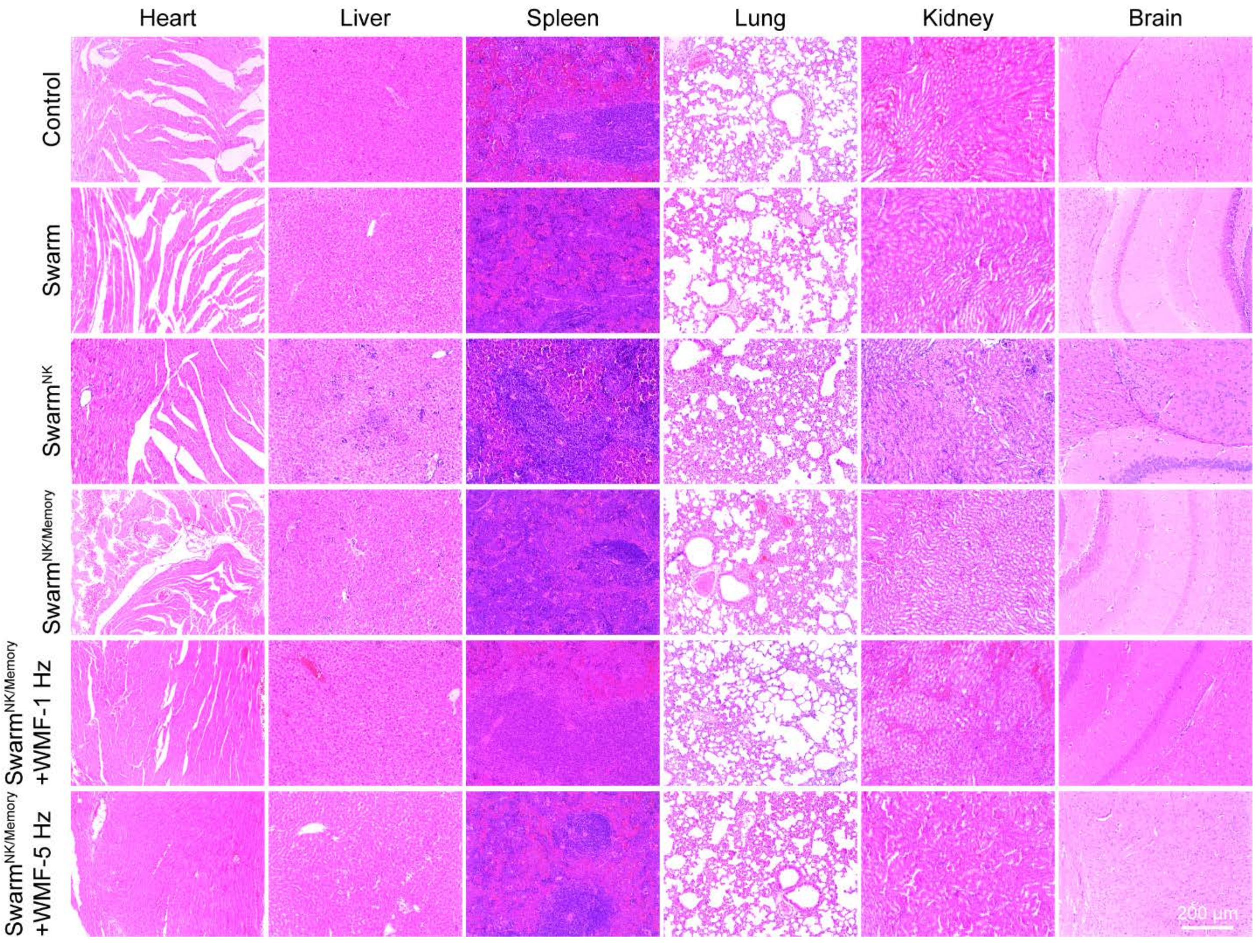

Fig. S32. H&E-stained histological sections of major organs, including the heart, liver, spleen, lung, kidney, and brain from PBS (1×) treated 4T1 tumor-bearing mice and 4T1 tumor-bearing mice administered intratumorally with probiotic microrobots on day 14. The tissue samples were collected at the end of the study.

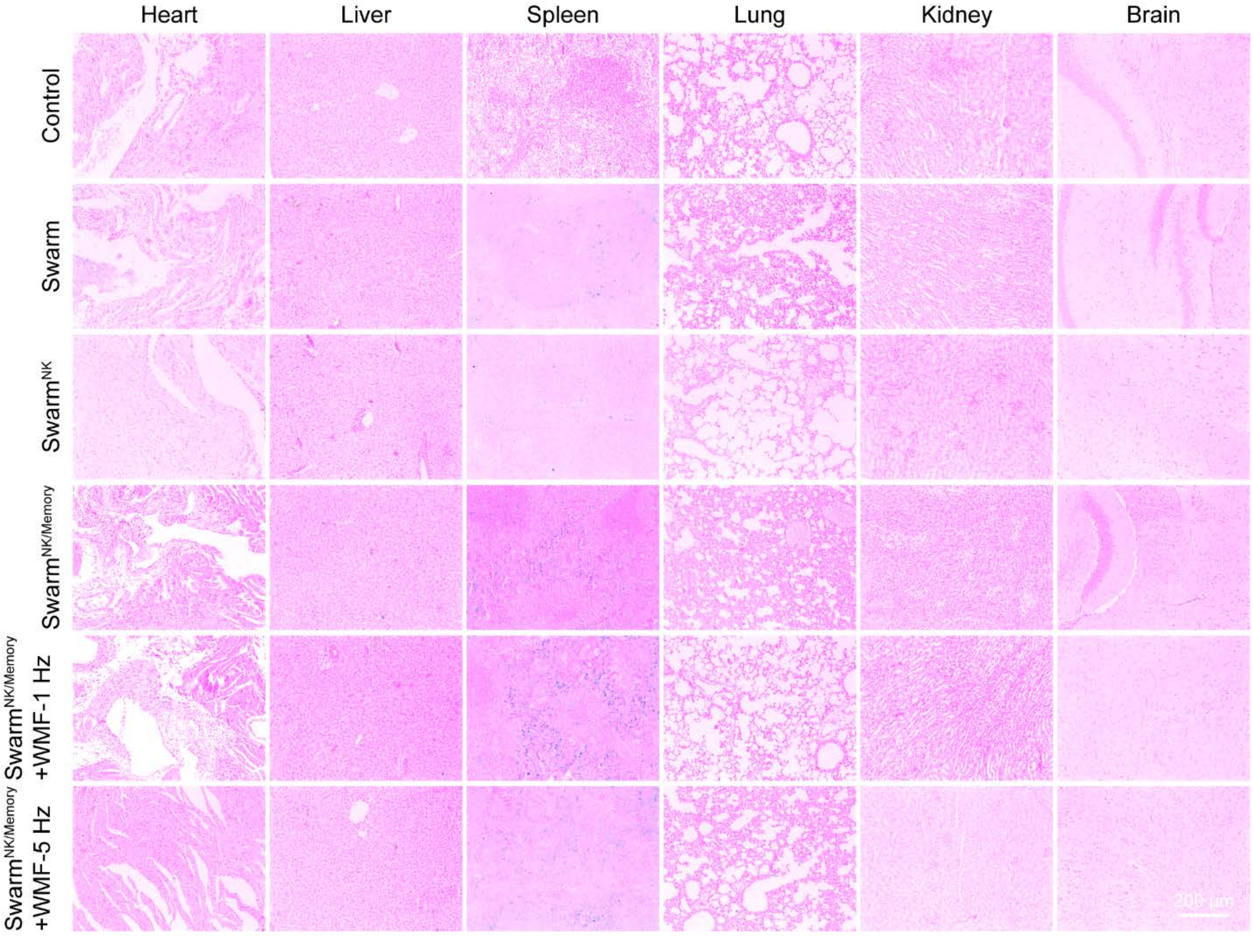

Fig. S33. Prussian blue-stained histological sections of major organs, including the heart, liver, spleen, lung, kidney, and brain from PBS (1×) treated 4T1 tumor-bearing mice and 4T1 tumor-bearing mice administered intratumorally with probiotic microrobots on day 14. The tissue samples were collected at the end of the study.

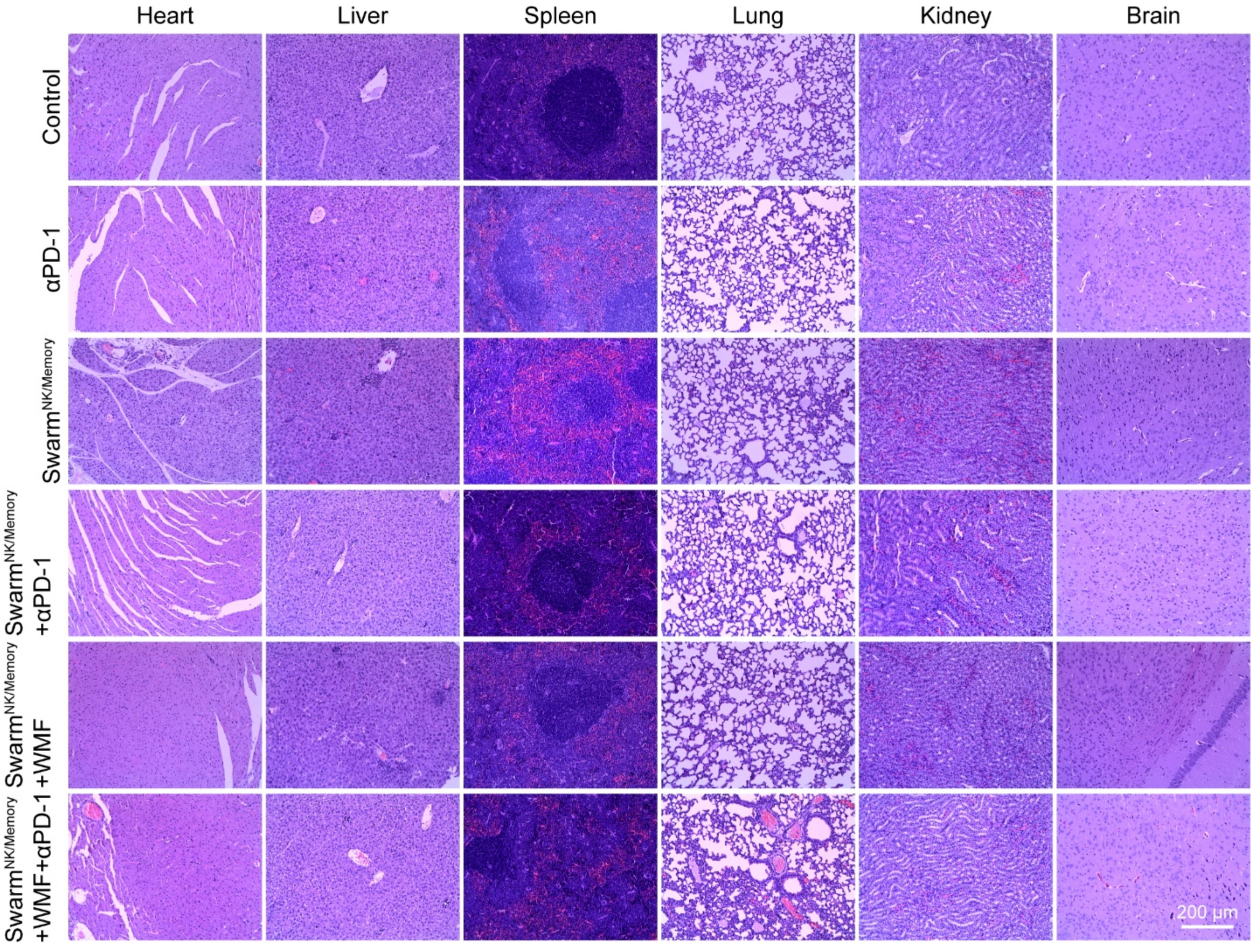

Fig. S34. H&E-stained histological sections of major organs, including the heart, liver, spleen, lung, kidney, and brain from PBS (1×) treated 4T1 tumor-bearing mice and 4T1 tumor-bearing mice administered intratumorally with probiotic microrobots on day 20. The tissue samples were collected at the end of the study.

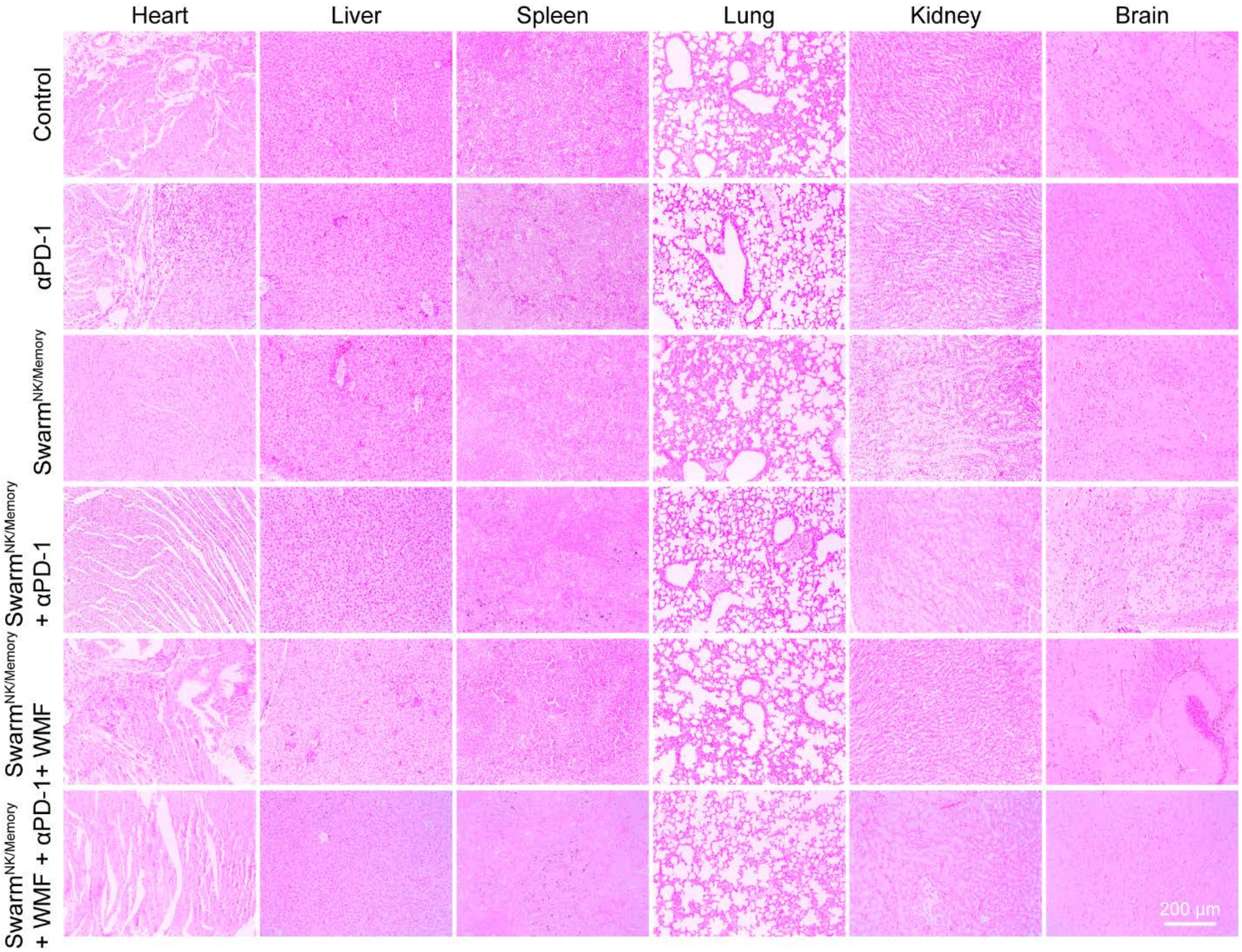

Fig. S35. Prussian blue-stained histological sections of major organs, including the heart, liver, spleen, lung, kidney, and brain from PBS (1×) treated 4T1 tumor-bearing mice and 4T1 tumor-bearing mice administered intratumorally with probiotic microrobots on day 20. The tissue samples were collected at the end of the study.

Supplementary Tables

Table S1. Comparison of previously reported memory modules

Memory modules	Size	Materials	Control	*In Vitro*	*In Vivo*	References
Resistive switching memory devices	Thickness 200 nm; Area 400 µm × 400 µm	$Ag/SiO_2/Au$	Applied pressures (20-10^3 Pa)	✓	–	Wang et al., 2013 (*19*)
Shape memory	2 mm^3	Hydrogen-bonded liquid crystal elastomers	Light (460 nm)	✓	–	Guo et al., 2024 (*24*)
Shape-memory alloy	Length 3 µm, thickness 1 µm	NiTi	Nanosecond UV laser source (35 mW)	✓	–	Kim et al., 2023 (*23*)
Threshold selector	200 nm	Ag/TiO_2	Electric field (0.2 V s^{-1})	✓	–	Song et al., 2015 (*21*)
Gold nanoparticles	13 nm	pH-sensitive gold nanoparticles	Visible light (455 + 635 nm)	✓	–	Zhang et al., 2019 (*22*)
Probiotic biohybrid microrobots	1.5 µm	Bxb1-attB-P7-attP	Alternating magnetic field (375 kHz)	✓	✓	**This work**

Table S2. Gene sequences of elements

Element	Gene Name	Sequence
**Sensor**	TcI	TCAGCCAAACGTCTCTTCAGGCCACTGACTAGCGATAACTTTCCCCACAACGGAACAAC TCTCATTGCATGGGATCATTGGGTACTGTGGGTTTAGTGGTTGTAAAAACACCTGACCGC TATCCCTGATCAGTTTCTTGAAGGTAAACTCATCACCCCCAAGTCTGGCTATGCAGAAAT CACCTGGCTCAACAGCCTGCTCAGGGTCAACGAGAATTAACATTCCGTCAGGAAAGCTT GGCTTGGAGCCTGTTGGTGCGGTCATGGAATTACCTTCAACCTCAAGCCAGAATGCAGA ATCACTGGCTTTTTTGGTTGTGCTTACCCATCTCTCCGCATCACCTTTGGTAAAGGTTCTA AGCTTAGGTGAGAACATCCCTGCCTGAACATGAGAAAAAACAGGGTACTCATACTCACT TCTAAGTGACGGCTGCATACTAACCGCTTCATACATCTCGTAGATTTCTCTGGCGATTGAA GGGCTAAATTCTTCAACGCTAACTTTGAGAATTTTTGTAAGCAATGCGGCGTTATAAGCA TTTAATGCATTGATGCCATTAAATAAAGCACCAACGCCTGACTGCCCCATCCCCATCTTGT CTGCGACAGATTCCTGGGATAAGCCAAGTTCATTTTTCTTTTTTTCATAAATTGCTTTAAG GCGACGTGCGTCCTCAAGCTGCTCTTGTGTTAATGGTTTCTTTTTTGTGCTCAT
**Actuator**	pR-pL	TTGACTATTTTACCTCTGGCGGTGATAATGGTTGCATGTACTAAGGAGGTTGTATGGAACA ACGCATAACCCTGAAAGATTATGCAATGCGCTTTGGGCAAACCAAGACAGCTAAAGATC TCTCACCTACCAAACAATGCCCCCCTGCAAAAAATAAATTCATATAAAAAACATACAGAT AACCATCTGCGGTGATAAATTATCTCTGGCGGTGTTGACATAAATACCACTGGCGGTGAT ACTG
	RBS	TCACACAGGAAACC
	attB Bxb1	TCGGCCGGCTTGTCGACGACGGCGGTCTCCGTCGTCAGGATCATCCGGGC

**Memory**	attP Bxb1	GGGTTTGTACCGTACACCACTGAGACCGCGGTGGTTGACCAGACAAACCACGA
	P7	AAGCTTTCCACACATTATAGGTACAAAAAGATGCGAAAGCAAATAAATTTTTT
	ssrA	GCAGCAAACGACGAAAACTACGCTTTAGCAGCTTAA
	Bxb1	AGAGCCCTGGTAGTCATCCGCCTGTCCCGCGTCACCGATGCTACGACTTCACCGGAGCG<br>TCAGCTGGAGTCTTGCCAGCAGCTCTGCGCCCAGCGCGGCTGGGACGTCGTCGGGGTA<br>GCGGAGGATCTGGACGTCTCCGGGGCGGTCGATCCGTTCGACCGGAAGCGCAGACCGA<br>ACCTGGCCCGGTGGCTAGCGTTCGAGGAGCAACCGTTTGACGTGATCGTGGCGTACCGG<br>GTAGATCGGTTGACCCGATCGATCCGGCATCTTCAGCAGCTGGTCCACTGGGCCGAGGA<br>CCACAAGAAGCTGGTCGTCTCCGCGACCGAAGCGCACTTCGATACGACGACGCCGTTTG<br>CGGCGGTCGTCATCGCGCTTATGGGAACGGTGGCGCAGATGGAATTAGAAGCGATCAAA<br>GAGCGGAACCGTTCGGCTGCGCATTTCAATATCCGCGCCGGGAAATACCGAGGATCCCT<br>GCCGCCGTGGGGATACCTGCCTACGCGCGTGGACGGGGAGTGGCGGCTGGTGCCGGAC<br>CCTGTGCAGCGAGAGCGCATCCTCGAGGTGTATCACCGCGTCGTCGACAACCACGAGCC<br>GCTGCATCTGGTGGCCCACGACCTGAACCGGCGTGGTGTCCTGTCGCCGAAGGACTACT<br>TCGCGCAGCTGCAAGGCCGCGAGCCGCAGGGCCGGGAGTGGTCGGCTACCGCGCTGAA<br>GCGATCGATGATCTCCGAGGCGATGCTCGGGTACGCGACTCTGAACGGTAAGACCGTCC<br>GAGACGACGACGGAGCCCCGCTGGTGCGGGCTGAGCCGATCCTGACCCGTGAGCAGCT<br>GGAGGCGCTGCGCGCCGAGCTCGTGAAGACCTCCCGGGCGAAGCCCGCGGTGTCTACC<br>CCGTCGCTGCTGCTGCGGGTGTTGTTCTGCGCGGTGTGCGGGGAGCCCGCGTACAAGTT<br>CGCCGGGGGAGGACGTAAGCACCCGCGCTACCGCTGCCGCTCGATGGGGTTCCCGAAG<br>CACTGCGGGAACGGCACGGTGGCGATGGCCGAGTGGGACGCGTTCTGCGAGGAGCAGG<br>TACTGGATCTGCTCGGGGACGCGGAGCGTCTGGAGAAAGTCTGGGTAGCGGGCTCGGA<br>CTCCGCGGTCGAACTCGCGGAGGTGAACGCGGAGCTGGTGGACCTGACGTCGCTGATC<br>GGCTCCCCGGCCTACCGGGCGGGCTCTCCGCAGCGAGAAGCACTGGATGCCCGTATTGC<br>GGCGCTGGCCGCGCGGCAAGAGGAGCTGGAGGGCCTGGAGGCTCGCCCGTCTGGCTGG<br>GAGTGGCGCGAGACCGGGCAGCGGTTCGGGGACTGGTGGCGGGAGCAGGACACCGCG<br>GCAAAGAACACCTGGCTTCGGTCGATGAACGTTCGGCTGACGTTCGACGTCCGCGGCG<br>GGCTGACTCGCACGATCGACTTCGGGGATCTTCAGGAGTACGAGCAGCATCTCAGGCTC<br>GGCAGCGTGGTCGAACGGCTACACACCGGGATGTCGACCGGC
**Therapy**	Nattokinase<br>(aprN,<br>pre-pro-nk)	GTATGAAAATAGTTATTTCGAGTCTCTACGGAAATAGCGAGAGATGATATACCTAAATAGA<br>GATAAAATCATCTCAAAAAAATGGGTCTACTAAAATATTATTCCATCTATTACAATAAATGC<br>ACAGAATAGTCTTTTAAGTAAGTCTACTCTGAATTTTTTAAAAGGAGAGGGTAAAGAGTG<br>AGAAGCAAAAAATTGTGGATCAGCTTGTTGTTTGCGTTAACGTTAATCTTTACGATGGCG<br>TTCAGCAACATGTCTGCGCAGGCTGCCGGAAAAAGCAGTACAGAAAAGAAATACATTGT<br>CGGATTTAAGCAGACAATGAGTGCCATGAGTTCCGCCAAGAAAAAGGATGTTATTTCTG<br>AAAAAGGCGGAAAGGTTCAAAAGCAATTTAAGTATGTTAACGCGGCCGCAGCAACATTG<br>GATGAAAAAGCTGTAAAAGAATTGAAAAAAGATCCGAGCGTTGCATATGTGGAAGAAG<br>ATCATATTGCACATGAATATGCGCAATCTGTTCCTTATGGCATTTCTCAAATTAAAGCGCC<br>GGCTCTTCACTCTCAAGGCTACACAGGCTCTAACGTAAAAGTAGCTGTTATCGACAGCG<br>GAATTGACTCTTCTCATCCTGACTTAAACGTCAGAGGCGGAGCAAGCTTCGTTCCTTCTG<br>AAACAAACCCATACCAGGACGGCAGTTCTCACGGTACGCATGTCGCCGGTACGATTGCC<br>GCTCTTAATAACTCAATCGGTGTTCTGGGCGTAGCGCCAAGCGCATCATTATATGCAGTAA<br>AAGTGCTTGATTCAACAGGAAGCGGCCAATATAGCTGGATTATTAACGGCATTGAGTGGG<br>CCATTTCCAACAATATGGATGTTATCAACATGAGCCTTGGCGGACCTACTGGTTCTACAGC<br>GCTGAAAACAGTAGTTGATAAAGCGGTTTCCAGCGGTATCGTCGTTGCTGCCGCAGCCG<br>GAAACGAAGGTTCATCCGGAAGCACAAGCACAGTCGGCTACCCTGCAAAATATCCTTCT<br>ACTATTGCAGTAGGTGCGGTAAACAGCAGCAACCAAAGAGCTTCATTCTCCAGCGTAGG<br>TTCTGAGCTTGATGTAATGGCTCCTGGCGTGTCCATCCAAAGCACACTTCCTGGAGGCAC<br>TTACGGCGCTTATAACGGAACGTCCATGGCGACTCCTCACGTTGCCGGAGCAGCAGCGC<br>TAATTCTTTCTAAGCACCCGACTTGGACAAACGCGCAAGTCCGTGATCGTTTAGAAAGC<br>ACTGCAACATATCTTGGAAACTCTTTCTACTATGGAAAAGGGTTAATCAACGTACAAGCA<br>GCTGCACAATAATAGTAAAAAGAAGCAGGTTCCTCCATACCTGCTTCTTTTTATTTGTCAG<br>CATCCTGATGTTCCGGCGCATTCTCTTCTTTCTCCGCATGTTGAATCCGTTCCATGATCGA<br>AGGATGGCTGCCTCTGAAAATCTTCACAAGCACCGGA
**Feedback**	mCherry	ATGGTGAGCAAGGGCGAGGAGGATAACATGGCCATCATCAAGGAGTTCATGCGCTTCA<br>AGGTGCACATGGAGGGCTCCGTGAACGGCCACGAGTTCGAGATCGAGGGCGAGGGCG<br>AGGGCCGCCCCTACGAGGGCACCCAGACCGCCAAGCTGAAGGTGACCAAGGGTGGCCC<br>CCTGCCCTTCGCCTGGGACATCCTGTCCCCTCAGTTCATGTACGGCTCCAAGGCCTACG<br>TGAAGCACCCCGCCGACATCCCCGACTACTTGAAGCTGTCCTTCCCCGAGGGCTTCAAG<br>TGGGAGCGCGTGATGAACTTCGAGGACGGCGGCGTGGTGACCGTGACCCAGGACTCCT<br>CCCTGCAGGACGGCGAGTTCATCTACAAGGTGAAGCTGCGCGGCACCAACTTCCCCTCC<br>GACGGCCCCGTAATGCAGAAGAAGACCATGGGCTGGGAGGCCTCCTCCGAGCGGATGT<br>ACCCCGAGGACGGCGCCCTGAAGGGCGAGATCAAGCAGAGGCTGAAGCTGAAGGACG<br>GCGGCCACTACGACGCTGAGGTCAAGACCACCTACAAGGCCAAGAAGCCCGTGCAGCT<br>GCCCGGCGCCTACAACGTCAACATCAAGTTGGACATCACCTCCCACAACGAGGACTAC<br>ACCATCGTGGAACAGTACGAACGCGCCGAGGGCCGCCACTCCACCGGCGGCATGGACG<br>AGCTGTACAAGTAA

Table S3. A comparative analysis of the degree of on-board integration (Ni/Lc) and yield of microrobots reported in the literature

Yield (Number/h)	On-board integration (Ni)	Characteristic length, Lc (μm)	Degree of on-board integration (Ni/Lc)	References
		Mechanical microrobots		
0.1-1	Sensor, Actuator, Actuation	500	0.006	Zhang et al., 2021 (*45*)
0.5	Sensor, Actuator, Actuation, Imaging, Therapy	500, 800, 1000	0.01, 0.00625, 0.005	Liu et al., 2024 (*46*)
0.00588	Sensor, Actuation, Imaging, Therapy	5003.92	0.000799	Gu et al., 2022 (*47*)
		Stem cell microrobots		
0.0416	Sensor, Actuator, Actuation, Output, Imaging, Therapy	293, 726, 550,450, 319.23	0.02, 0.008, 0.01, 0.013, 0.018	Shen et al., 2025 (*48*)
0.0416	Sensor, Actuator, Actuation, Imaging	519, 1292, 938, 653, 438	0.0077, 0.003, 0.0042, 0.006, 0.0091	Wang et al., 2021 (*49*)
2.08	Sensor, Actuation, Imaging, Therapy	161.22	0.0248	Wang et al., 2024 (*50*)
		Immune cell microrobots		
9.77×10^5	Sensor, Actuator, Actuation, Imaging, Therapy	7.42	0.67	Zhang et al., 2021 (*16*)
5×10^5	Sensor, Actuation, Imaging, Therapy	7.14	0.56	Gao et al., 2025 (*51*)
		Algae microrobots		
1.918×10^7	Sensor, Actuation, Imaging	8.4	0.35	Akolpoglu et al., 2025 (*52*)
3.125×10^7	Sensor, Actuation, Imaging, Therapy	2.64	1.51	Li et al., 2025 (*53*)
2×10^5	Sensor, Actuation, Imaging, Therapy	2.18	1.83	Zhang et al., 2022 (*54*)
2.78×10^6	Sensor, Actuation, Imaging, Therapy	5.68	0.7	Zhang et al., 2024 (*55*)
3.03×10^6	Sensor, Actuation, Imaging, Therapy	6.67	0.59	Zhang et al., 2022 (*56*)
		Bacterial microrobots		

1.38×10^{8}	Sensor, Actuation, Imaging, Therapy	2.41	1.65	Akolpoglu et al., 2022 (*18*)
1.66×10^{8}	Sensor, Actuator, Actuation, Output, Imaging, Therapy	1.5	4	Chen et al., 2022 (*33*) **Our previous work**
3.32×10^{7}	Sensor, Actuator, Actuation, Memory, Suppressor, Output, Imaging, Therapy	1.5	5.33	**This work**

Table S4. Comparison of previously reported magnetic field setups

**Magnet type**	**Magnetic field parameters**	**Working place**	**Motion form**	**Application scenarios (Penetration depth)**	**Reference**
Electromagnetic coils and permanent magnet	12-20 mT 14-24 Hz	80-250 μm (Penetration depth)	Rotation and Translation	Cell and animal (3-4 cm)	Gwisai et al., 2022 (*17*)
Electromagnetic coils and permanent magnet	5-26 mT	Micron-scale channels	Rotation and Translation	Microfluidic chips, porous gels, tumor spheroids (2-5 mm)	Akolpoglu et al., 2022 (*18*)
Electromagnetic coils	30 mT Gradients: 1 T m^{-1}	20 × 20 × 20 cm^3	Translation and Rotation	Animal-porcine model (~2.5 cm)	Landers et al., 2025 (*62*)
Electromagnetic coils	5-50 mT Gradients: 250 mT m^{-1}	Height 40 cm	Translation and Rotation	Animal-porcine model (~2.5 cm)	Gervasoni et al., 2024 (*63*)
Electromagnetic coils and NdFeB permanent magnet	29.9 mT 0-15 Hz 100 mT	50 cm^3	Translation and Rotation	Silicone vascular model (1-4 cm)	Du et al., 2025(*64*)
Electromagnetic coils and spherical permanent magnet	15 mT/ 300 mT 200 Hz	25 cm ×25 cm ×10 cm	Translation and Rotation	Centi-milli-micro (1-3 mm)	Fan et al., 2022 (*65*)

Electromagnetic coils and permanent magnet	15 mT<br>12 Hz	Diameter 5.5 cm<br>Length 8.5 cm	Rotation<br>Oscillation<br>Translation	Cell and animal (3-4 cm)	Mirkhani et al., 2024 (*66*)

Table S5. Clinical administration routes for bacteria-mediated cancer therapy

Bacterial strain	Administration route	Trial phase	Tumor type (number of patients)	References
E. coli Nissle 1917	intratumoral	I	Refractory advanced cancers (melanoma, sarcoma and esophageal cancer) (17)	Luke et al., 2023 (*29*)
C. novyi-NT	intratumoral	I	Advanced-stage solid tumors (24)	Janku et al., 2021 (*71*)
C. novyi-NT	intratumoral	I	Advanced-stage solid tumors (10)	Nelson et al., 2023 (*72*)
L. monocytogenes	intratumoral	II	Metastatic pancreatic ductal adenocarcinoma (PDAC) (90)	Le et al., 2015 (*73*)
S. Typhimurium	intravenous	I	Metastatic melanoma (25)	Toso et al., 2002 (*74*)
L. monocytogenes	intravenous	II	Advanced-stage cervical carcinomas (50)	Huh et al., 2020 (*75*)
L. monocytogenes	intravenous	I	Metastatic castration resistant prostate cancer (26)	Drake et al., 2022 (*76*)
S. Typhimurium	oral	I	Advanced-stage pancreatic cancer (45)	Schmitz-Winnenthal et al., 2015 (*77*)
S. Typhimurium	oral	I	Liver-metastatic solid tumors (22)	Gniadek et al., 2020 (*78*)

Supplementary Movies

Movie S1. Aggregation of probiotic microrobot swarm under rotating magnetic field in simulation. This video illustrates the dynamic aggregation process of probiotic microrobots under a rotating magnetic field, consistent with the behavior described in Fig. 3A and the main text. Under the RMF, the microrobots initially align into chain-like formations and rotate synchronously with the external field. As rotation continues, these chains progressively destabilize, collapse, and

reorganize into a densely aggregated swarm. The visualization highlights the magnetic field mediated transition from dispersed individual robots to a compact collective structure.

Movie S2. Tumbling mode locomotion of probiotic microrobot swarm in real experiment and simulation. This video shows the tumbling locomotion mode of the microrobot swarm under magnetic actuation, presented in both real experiments and numerical simulations. The swarm exhibits periodic flipping and forward displacement, validating the consistency between simulation and experimental observations.

Movie S3. Oscillating mode locomotion of probiotic microrobot swarm in real experiment and simulation. This video illustrates the oscillating locomotion mode of the microrobot swarm. Driven by an alternating magnetic field, the swarm performs rhythmic back-and-forth oscillations, and the simulation reproduces the key features observed in the real experiment.

Movie S4. Waving mode locomotion of probiotic microrobot swarm in real experiment and simulation. This video demonstrates the waving locomotion mode of the microrobot swarm. Under 3D magnetic field control, the swarm exhibits coordinated wave-like motion, achieving efficient forward propulsion in both experimental and simulated environments.

Movie S5. Waving mode locomotion of individual probiotic microrobot in real experiment and simulation. This video presents the waving locomotion mode of a single probiotic microrobot. The individual robot performs periodic waving cycles that generate directional propulsion, and the simulated trajectory matches the experimentally observed motion pattern.

Movie S6. Positive-feedback softening in waving-driven penetration in theoretical visualization. This video provides a detailed theoretical visualization of the positive-feedback softening process that enables deeper penetration of the probiotic microrobot. The magnetothermal effect initiates enzymatic softening within the microenvironment. As the softening front gradually expands, microrobots advance along this softened path with reduced mechanical resistance. The video sequentially visualizes the evolving stiffness, the propagation of the softening zone, and the resulting increase in penetration depth over time.
